# Manual delineation approaches for direct imaging of the subcortex

**DOI:** 10.1007/s00429-021-02400-x

**Published:** 2021-10-29

**Authors:** Anneke Alkemade, Martijn J. Mulder, Anne C. Trutti, Birte U. Forstmann

**Affiliations:** 1 Integrative Model-Based Cognitive Neuroscience Research Unit, University of Amsterdam, Nieuwe Achtergracht 129B | Room G3.08, Postbus 15926, 1001 NK Amsterdam, The Netherlands; 2 Psychology and Social Sciences, University of Utrecht, Utrecht, The Netherlands; 3 Institute of Psychology, Leiden University, Leiden, The Netherlands

**Keywords:** Ultra-high field 7 Tesla structural MRI, Subcortex, Probabilistic maps, Basal ganglia, Thalamus

## Abstract

The growing interest in the human subcortex is accompanied by an increasing number of parcellation procedures to identify deep brain structures in magnetic resonance imaging (MRI) contrasts. Manual procedures continue to form the gold standard for parcellating brain structures and is used for the validation of automated approaches. Performing manual parcellations is a tedious process which requires a systematic and reproducible approach. For this purpose, we created a series of protocols for the anatomical delineation of 21 individual subcortical structures. The intelligibility of the protocols was assessed by calculating Dice similarity coefficients for ten healthy volunteers. In addition, dilated Dice coefficients showed that manual parcellations created using these protocols can provide high-quality training data for automated algorithms. Here, we share the protocols, together with three example MRI datasets and the created manual delineations. The protocols can be applied to create high-quality training data for automated parcellation procedures, as well as for further validation of existing procedures and are shared without restrictions with the research community.

## Introduction

Anatomical atlases are essential for directing brain research, interpreting and standardizing results, as well as guiding surgery (Devlin and Poldrack 2007; Forstmann et al. 2017). Particularly atlases of the human subcortex continue to gain interest in view of the growing number of structures that could potentially serve as targets for deep brain stimulation surgery (DBS) (Lozano et al. 2019). The golden standard approach for the identification and parcellation of brain structures is the manual delineation on individual magnetic resonance imaging (MRI) contrasts in their native space. This allows the subsequent creation of probabilistic atlases, which capture the interindividual anatomical variation in the brain, via registration to standard MRI-space (Mazziotta et al. 1995).

Creating 3-dimensional (3D) delineations of the approximately 455 subcortical structures using individual MRI contrasts represents a major feat (Alkemade et al. 2013). To perform manual delineation in a consistent and reproducible manner clear anatomical descriptions are indispensable. In view of the many major and minor anatomical differences between people, flexible knowledge of the human brain is crucial for performing delineations. It can be challenging to mentally image the 3D structure together with the relative orientation of individual brain nuclei on MRI contrasts using a histological atlas such as those published by Schaltenbrand and Wahren (1977) and Mai et al. (2015). Familiarization with the 3D structure and relative orientation of individual subcortical brain structures will, therefore, contribute to the development of the flexible knowledge on human neuroanatomy, which is crucial to MRI parcellation efforts.

To facilitate delineation efforts on the human subcortex, we created a series of illustrated parcellation protocols which can be reused to identify and outline a set of 21 individual structures. Each protocol starts with a post mortem blockface image derived from our previous studies for anatomical orientation (Alkemade et al. 2020b). The list of 21 structures is by far not exhaustive and can viewed as a first step that can be extended in the future. Structures included were selected based on their visibility, their potential role as a target structure for deep brain stimulation surgery, or their practical use as an anatomical landmark. We would like to note that these structures include aggregations of anatomical substructures such as the thalamus (Tha), as well as deeper brain structures that are not of subcortical origin, such as the amygdala, and the ventricular system. This work is not intended to determine whether brain structures belong to the cortex or subcortex, but to share delineation protocols that can be reused for the delineation of individual brain structures. 7 Tesla (T) MRI scans, with submillimeter resolution were used to create these descriptions, which has resulted in visual representations of the delineated structures in all anatomical planes (Alkemade et al. 2020a). These manual delineations have also formed the basis for training of the multi-contrast anatomical subcortical structures parcellation (MASSP) algorithm which has been published previously (Bazin et al. 2020). To improve flexible knowledge, we present individual delineation instructions on three separate datasets. The delineation protocols were tested by creation of manual delineations by two independent raters on a training dataset of ten young healthy individuals. Indicators of interrater agreement were used as a measure for the reliability of the resulting parcellations, and the usability of the protocols.

## Materials and methods

All structures were parcellated in FSLview or FSLeyes (fsl.fmrib.ox.ac.uk) using MRI contrasts obtained from a subset of ten healthy volunteers (age 20–49, eight females) from the Amsterdam ultra-high field adult lifespan database (AHEAD) described previously (Alkemade et al. 2020a). All participants were healthy as assessed through self-reports.

Available contrasts included the following: T1-weighted, T1- and R1-maps, T2*-maps, and quantitative susceptibility mapping. Contrasts for the delineations of individual borders were determined experimentally and per structure. Raters varied across structures and included both experienced and novel raters. Novel raters first performed independent test parcellations on which feedback was provided by an experienced anatomist (AA) on additional available scans. Parcellations were performed under standardized conditions in individual space, in which the order of participants as well as the first hemisphere to delineate was randomized. Lighting circumstances were kept stable, and raters had access to high-resolution computer screens and drawing pads for parcellation purposes.

Contrasts were calculated from a single MRI acquisition using the MP2RAGEME sequence described previously (Caan et al. 2019). The MP2RAGEME sequence allows the calculation of T1 and T2* derived contrasts from a single scan acquisition, resulting in intrinsic alignment of the resulting contrasts.

Dice similarity coefficients and dilated Dice coefficients were calculated based on manual delineations of two independent raters, as described previously (Dice and Dice 1945; Bazin et al. 2016, 2020; Alkemade et al. 2020a; Trutti et al. 2021). Protocol layouts were then standardized to create a coherent series of guidelines to delineate 21 Individual structures (see Table 1). Parcellation procedures are presented below and are illustrated using screen shots of the resulting masks in the coronal, axial, and sagittal plane. All procedures contain a concise description of the structure and an image from a human brain cut in serial coronal sections for anatomical orientation (Alkemade et al. 2020b). Additionally, a concise description of the procedure and illustrations of the parcellations at various anatomical levels on T1-weighted and quantitative susceptibility mapping (QSM) contrasts are included. Table 1 indicates the MRI contrast used for the parcellation procedure for each individual structure. We would like to note that the visibility of individual anatomical borders varies across contrasts. This potentially hampers the reuse of the protocols for parcellations on clinical scans, in which not all MR contrasts presented here are included. We share manual parcellations from single raters together with the MRI contrasts of three individual participants. This allows users to inspect the data in detail in the coronal, axial and sagittal plane, on T1-weighted, T1-maps, T2*-maps, R1-maps, R2*-maps, and QSM, and help to determine their usability on other datasets including clinical scans acquired using lower field strengths.

**Table 1:** Dice similarity coefficients reflecting agreement between manual delineations

Structure	Abbreviation	MRI contrast used for parcellations	Dice coefficient left hemisphere (s.e.m.)	Dice coefficient right hemisphere (s.e.m.)	Dice coefficient average (s.e.m.)	Dilated Dice coefficient left hemisphere (s.e.m.)	Dilated Dice coefficient right hemisphere (s.e.m.)	Dilated Dice coefficient average (s.e.m.)
Claustrum	Cl	T1-w	0.73 (0.01)	0.73 (0.01)	**0.73 (0.01)**	0.92 (0.01)	0.91 (0.01)	**0.91 (0.01)**
Amygdala	Amg	T1-w	0.88 (0.01)	0.86 (0.01)	**0.87 (0.01)**	0.98 (0.01)	0.97 (0.01)	**0.98 (0.00)**
Fornix*	fx	T1-w			**0.83 (0.01)**			**0.96 (0.01)**
Globus Pallidus externa	GPe	T1-w/QSM	0.81 (0.01)	0.81 (0.02)	**0.81 (0.01)**	0.97 (0.00)	0.97 (0.01)	**0.97 (0.01)**
Globus Pallidus interna	GPi	T1-w/QSM	0.76 (0.02)	0.76 (0.02)	**0.76 (0.02)**	0.96 (0.01)	0.95 (0.01)	**0.95 (0.01)**
Inferior Colliculus	iCo	T2*-w/R2*-maps	0.83 (0.02)	0.84 (0.01)	**0.83 (0.01)**	0.98 (0.01)	0.99 (0.01)	**0.98 (0.01)**
Superior Colliculus	sCo	T2*-w/R2*-maps	0.72 (0.03)	0.77 (0.03)	**0.74 (0.03)**	0.92 (0.02)	0.95 (0.02)	**0.93 (0.02)**
Internal Capsule	ic	T1-w	0.73 (0.01)	0.74 (0.01)	**0.73 (0.01)**	0.92 (0.01)	0.93 (0.01)	**0.92 (0.01)**
Lateral Habenula	LH	T1-w	0.82 (0.02)	0.79 (0.01)	**0.81 (0.01)**	0.97 (0.02)	0.95 (0.02)	**0.96 (0.02)**
Periaqueductal grey	PAG	T1-w	0.79 (0.02)	0.80 (0.01)	**0.79 (0.01)**	0.96 (0.01)	0.96 (0.00)	**0.96 (0.01)**
Pedunculopontine Nucleus	PPN	T1-map	0.66 (0.02)	0.67 (0.02)	**0.67 (0.02)**	0.92 (0.02)	0.90 (0.02)	**0.91 (0.02)**
Red Nucleus	RN	QSM	0.88 (0.01)	0.87 (0.01)	**0.88 (0.01)**	1.00 (0.00)	0.99 (0.00)	**1.00 (0.00)**
Substantia Nigra	SN	QSM	0.84 (0.01)	0.84 (0.01)	**0.84 (0.01)**	0.98 (0.01)	0.99 (0.00)	**0.98 (0.01)**
Striatum	Str	T1-w/QSM	0.90 (0.00)	0.90 (0.00)	**0.90 (0.00)**	0.99 (0.00)	0.99 (0.00)	**0.99 (0.00)**
Subcallosal Cingulate Gyrus	SCG	T1-map	0.54 (0.04)	0.68 (0.02)	**0.61 (0.02)**	0.78 (0.05)	0.92 (0.02)	**0.85 (0.02)**
Thalamus	Tha	T1-w	0.88 (0.01)	0.88 (0.01)	**0.88 (0.01)**	0.97 (0.00)	0.98 (0.00)	**0.97 (0.00)**
Lateral ventricles	LV	T1-w	0.91 (0.01)	0.92 (0.01)	**0.91 (0.01)**	0.97 (0.01)	0.97 (0.01)	**0.97 (0.01)**
Third Ventricle*	3V	T1-w			**0.80 (0.02)**			**0.94 (0.02)**
Fourth Ventricle*	4V	T1-w			**0.88 (0.02)**			**0.96 (0.01)**
Ventral Tegmental Area	VTA	QSM/midbrain contrast	0.58 (0.01)	0.57 (0.02)	**0.58 (0.01)**	0.82 (0.02)	0.82 (0.02)	**0.82 (0.02)**
Subthalamic Nucleus	STN	QSM	0.80 (0.01)	0.80 (0.01)	**0.80 (0.01)**	0.98 (0.00)	0.99 (0.00)	**0.99 (0.00)**

Averages across hemispheres are presented in bold

*Parcellations of structures crossing the midline were not performed separately for the left and right hemispheres

*s.e.m.* standard error of the mean

### Globus pallidus internal and external segment

**Fig. 1:**
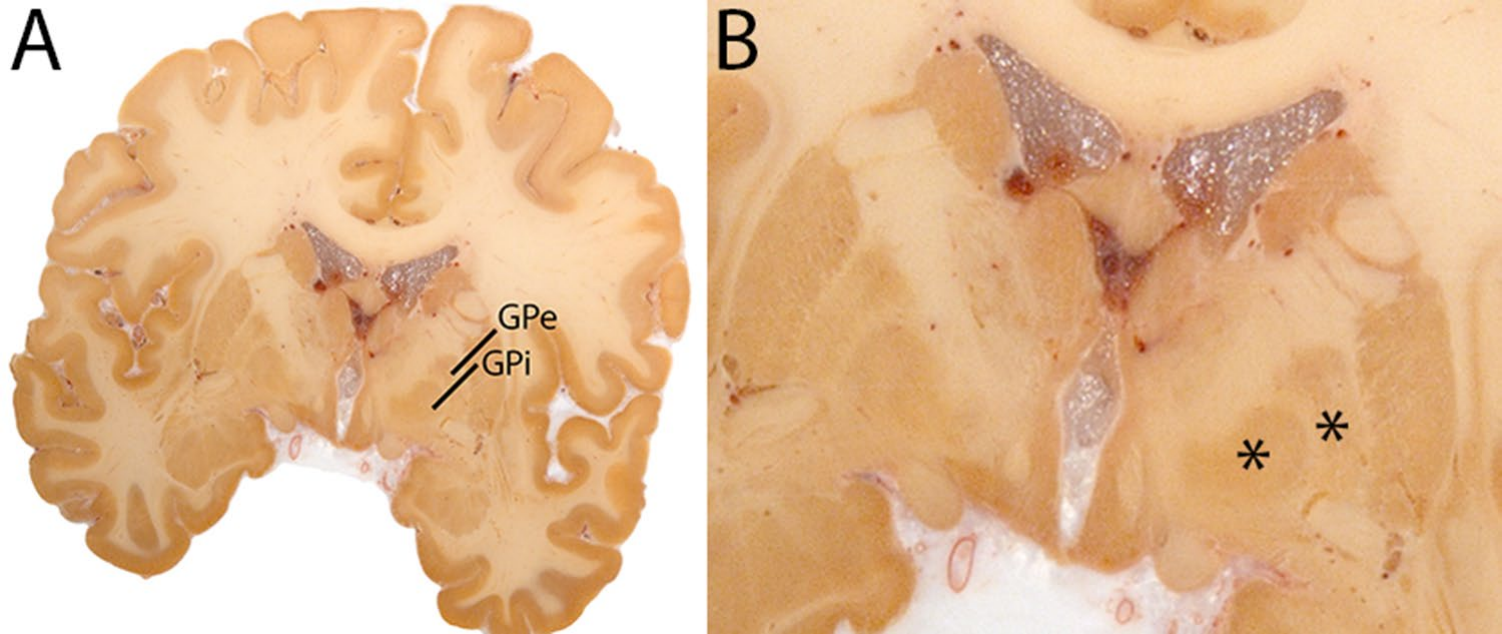
**A** Globus pallidus internal and external segment (GPi and GPe) in a central coronal view. **B** Magnification, asterisks indicate regions of interest

The Globus Pallidus (GP) is a crescent structure with an elongated shaped oriented along the rostrocaudal brain axis. The GP comprises an internal segment (GPi), which is located medial to the external segment (GPe). The segments are separated by the medial medullary lamina (mml), and the GPe is separated from the striatum by the lateral medullary lamina (lml). Surrounding landmarks include the Tha located posterior to the GP, and the caudate nucleus which is located dorsally. Laterally the putamen is found, and medioventral to the GP the subthalamic nucleus (STN) and the substantia nigra (SN). At more central levels, the GP is located superior to the SN and STN, and at the caudal extent to the red nucleus (RN) (Fig. 1).

#### Parcellation

The GPe is first identified in the coronal view. The border between the GPe and the striatum (Str) is delineated on T1-weighted contrasts. This is done on consecutive coronal slices following the structure first in the anterior, and then in the posterior direction. Additionally, the transverse plane can be used to pinpoint the striatal-GPe border if required. The internal capsule (ic) visible on T1-weighted contrasts is used to define the rostral border of the GPe in the axial view. Subsequently, parcellations are continued in the QSM contrast, using the coronal view moving in an anterior direction. In the QSM contrast, the mml can be identified, allowing the separation of GPi and GPe. At the level of the optic chiasm, the anterior part of the GPe has a rounded or triangular shape at levels where the GPi is absent. The size of the GPe decreases in an anterior direction until disappearing rostral to the anterior commissure. Note that the anterior commissure can cross through the GPe; however, given the limited visibility of the subcommissural GPe, this part of the GPe is not included in the delineations. At central levels of the GP, the GPi is located medial to the GPe presenting as a rounded or triangular shape. At more caudal levels, the GPe shifts in a lateral direction assuming a crescent shape over the GPi, which at the same time increases in size. At the level of the SN and RN, the GPe and GPi gradually decrease in size. First, the GPi and eventually also the GPe disappears usually at central levels of the RN.

**Fig. 2:**
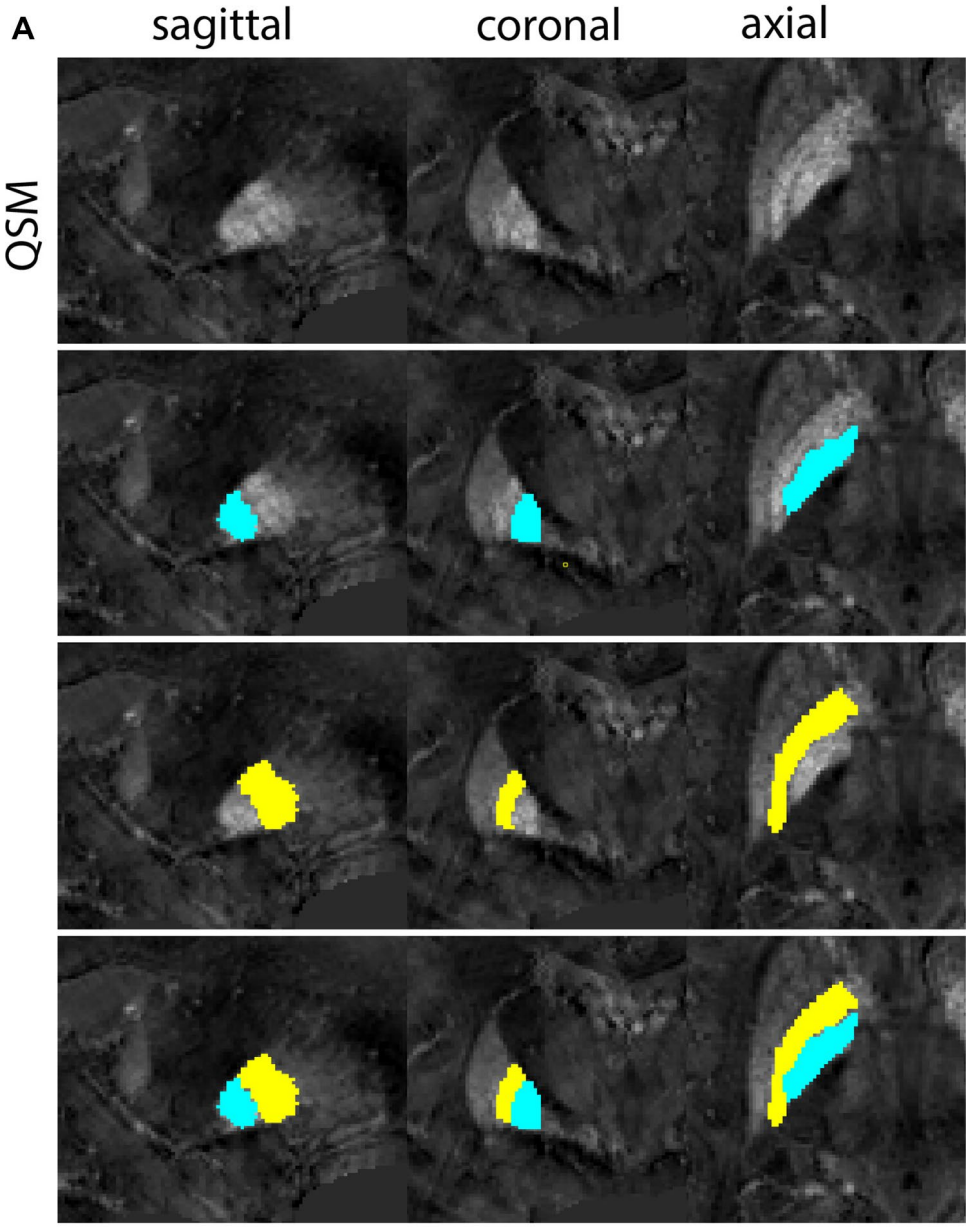

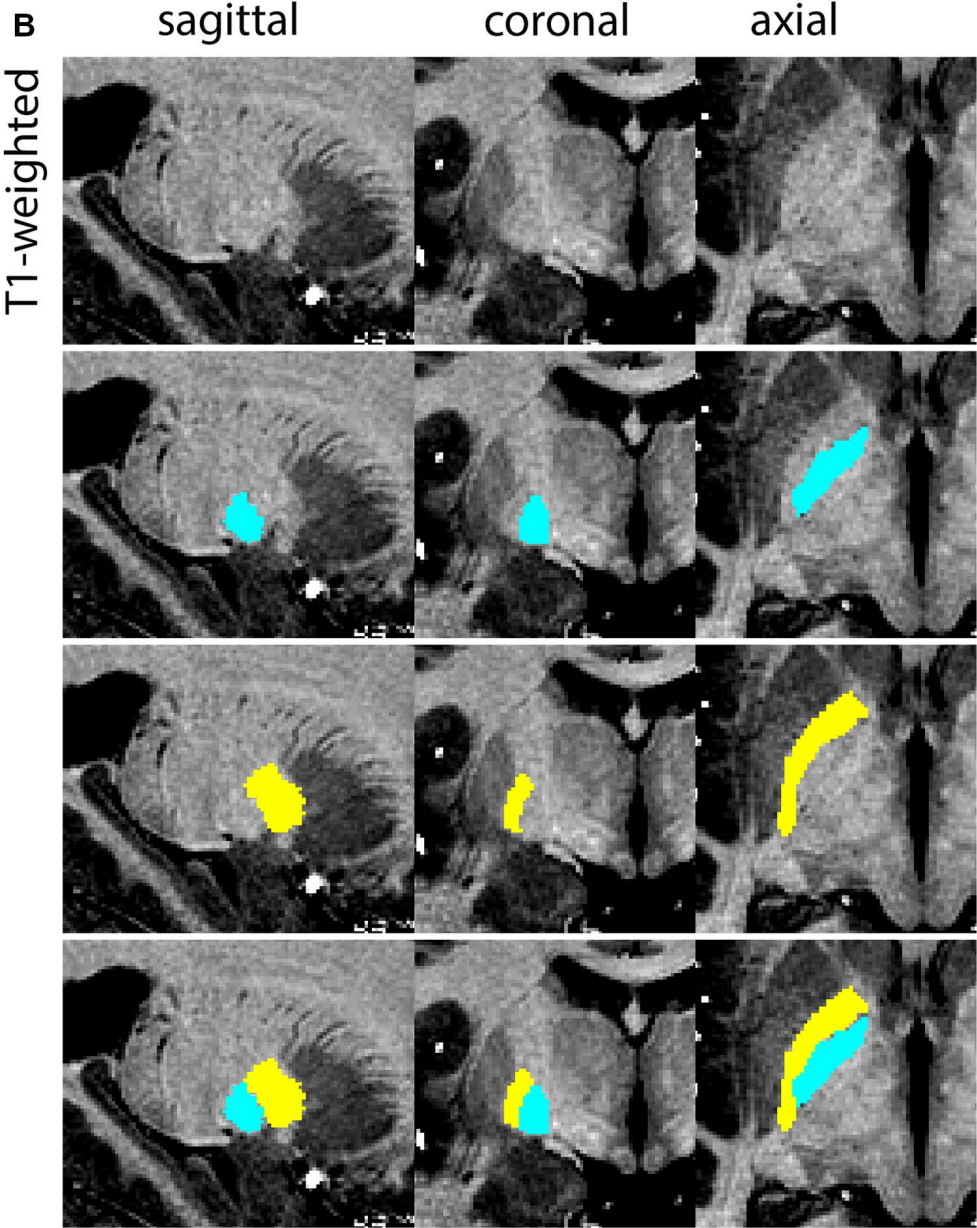

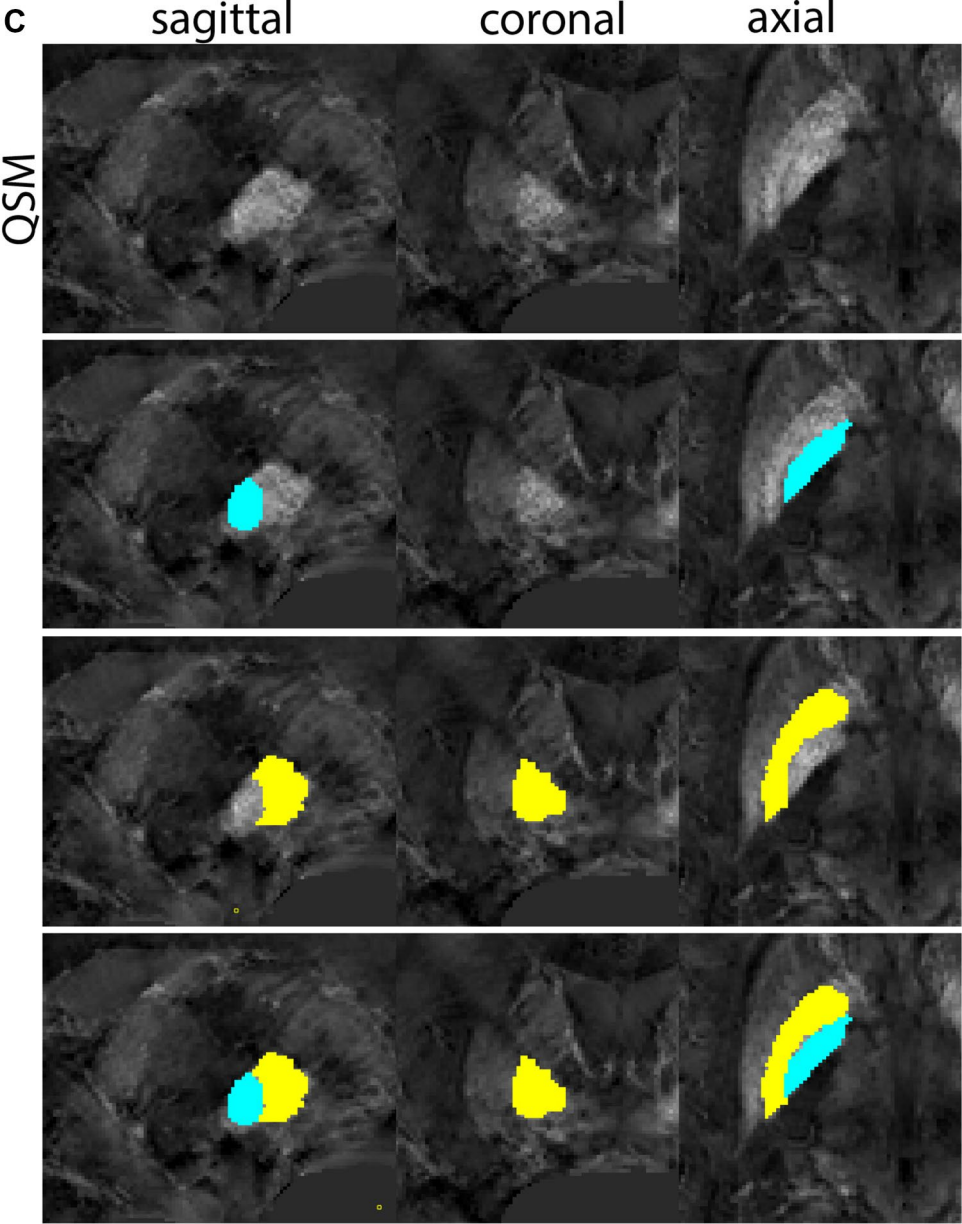

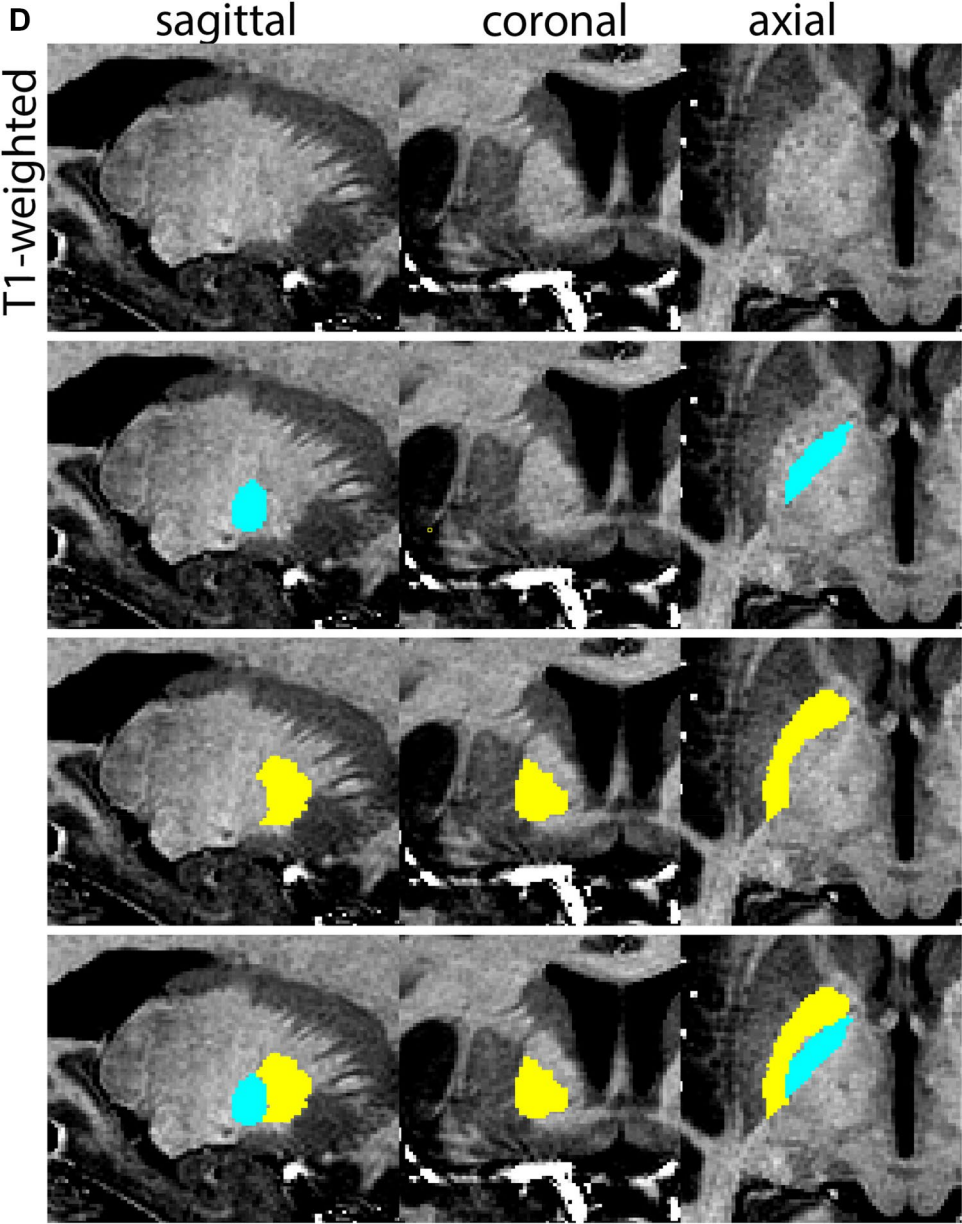

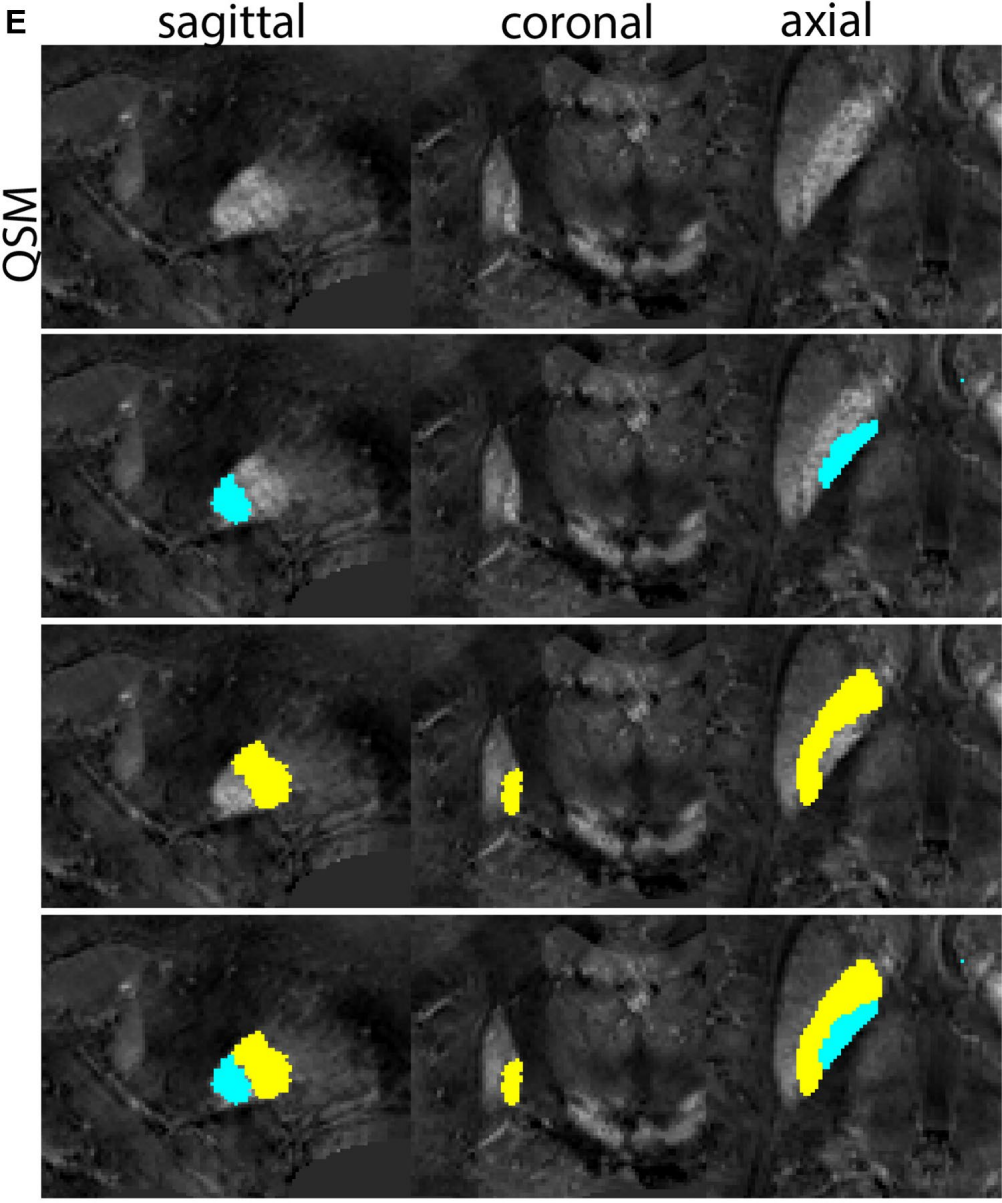

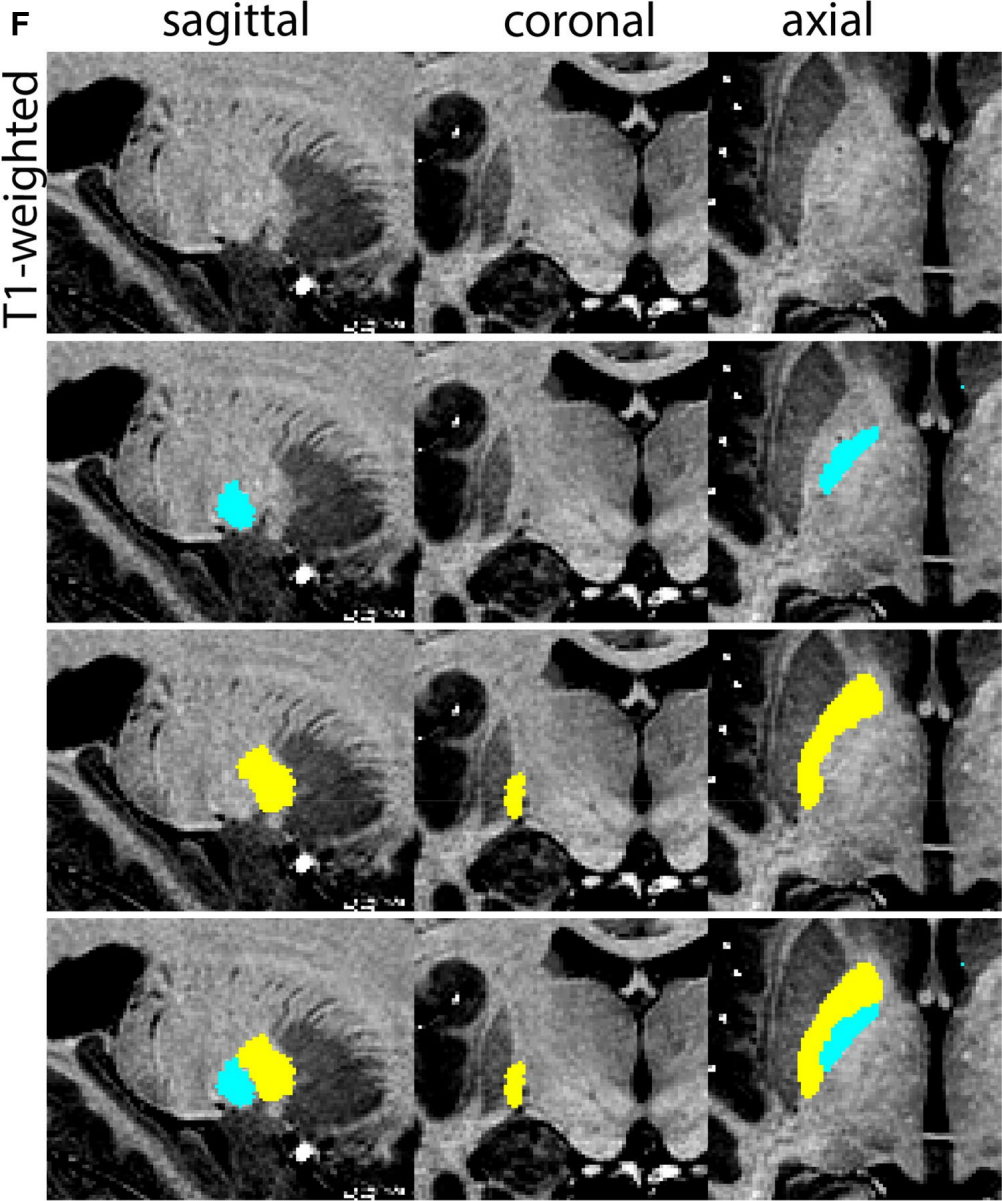
**a** Globus pallidus internal and external segment (GPi and GPe) in a central view on QSM. Top row QSM contrast. Note the separation between the GPi and GPe by the hypointense mml. Yellow = GPe, Light blue = GPi. **b** Globus pallidus internal and external segment (GPi and GPe) at the level of the mamillary bodies on a T1-weighted contrast. Top row T1-weigthed contrast. Note the boundary between the GPe and the hypointense striatum which is located lateral to the GPe, and the lack of contrast between the GPi and in in the T1-w contrast. Yellow = GPe, Light blue = GPi. **c** Globus pallidus internal and external segment (GPi and GPe) in the coronal plane on the QSM contrast at the level where the anterior commissure crosses the 3V (not visible on QSM). Top row QSM contrast. Note the absence of the GPi from the coronal (middle) panel at these rostral levels. Note the increased visibility of the dorsal GPi/e border as a result of the hypointense appearance of the internal capsule. Yellow = GPe, Light blue = GPi. **d** Globus pallidus internal and external segment (GPi and GPe) in a rostral view on T1-weighted contrast at a level in which the anterior commissure crosses the 3V. Top row T1-weigthed contrast. Note the limited contrast between the GPe, the internal capsule and the anterior commissure at this level. Note the limited visibility of the ventral pallidum. Yellow = GPe, Light blue = GPi. **e** Globus pallidus internal and external segment (GPi and GPe) in a caudal coronal view on a QSM contrast. Note the clear visibility of other iron-rich structures including the STN and SN at this level. Top row QSM contrast. Note the limited contrast between the GPe, the internal capsule and the anterior commissure at this level. Yellow = GPe, Light blue = GPi. **f** Globus pallidus internal and external segment (GPi and GPe) in a more caudal view on a T1-weighted contrast. Note the reduced visibility of other iron-rich structures including the STN and SN at this level as compared to the QSM contrast in Fig. 4a. Top row T1-weighted image, note the clear visibility of the border between the Str and the GPe. Yellow = GPe, Light blue = GPi

Next, both the T1-weighted and QSM contrasts are checked for consistency of the delineations, and the 3D view is used for confirmation of the expected anatomical shape. Figure 2 illustrates the visibility of the GPi/e on QSM and T1-weighted contrasts at various anatomical levels.

### Subthalamic nucleus

**Fig. 3:**
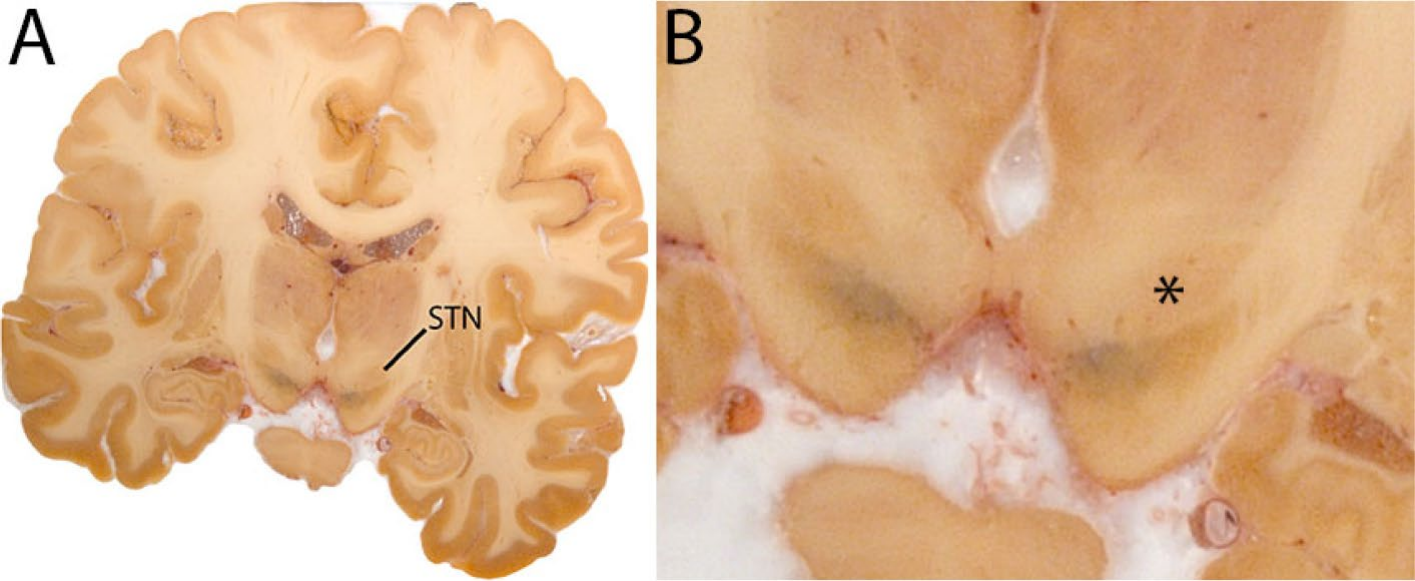
**A** Subthalamic nucleus (STN) in a central coronal view. **B** Magnification, the asterisk indicates the STN

The STN is a biconvex lens-shaped nucleus located between the RN and the comb system at the dorsomedial border of the SN (Fig. 3). The STN is an iron-rich structure which results in a hyperintense appearance in QSM contrasts.

#### Parcellation

**Fig. 4:**
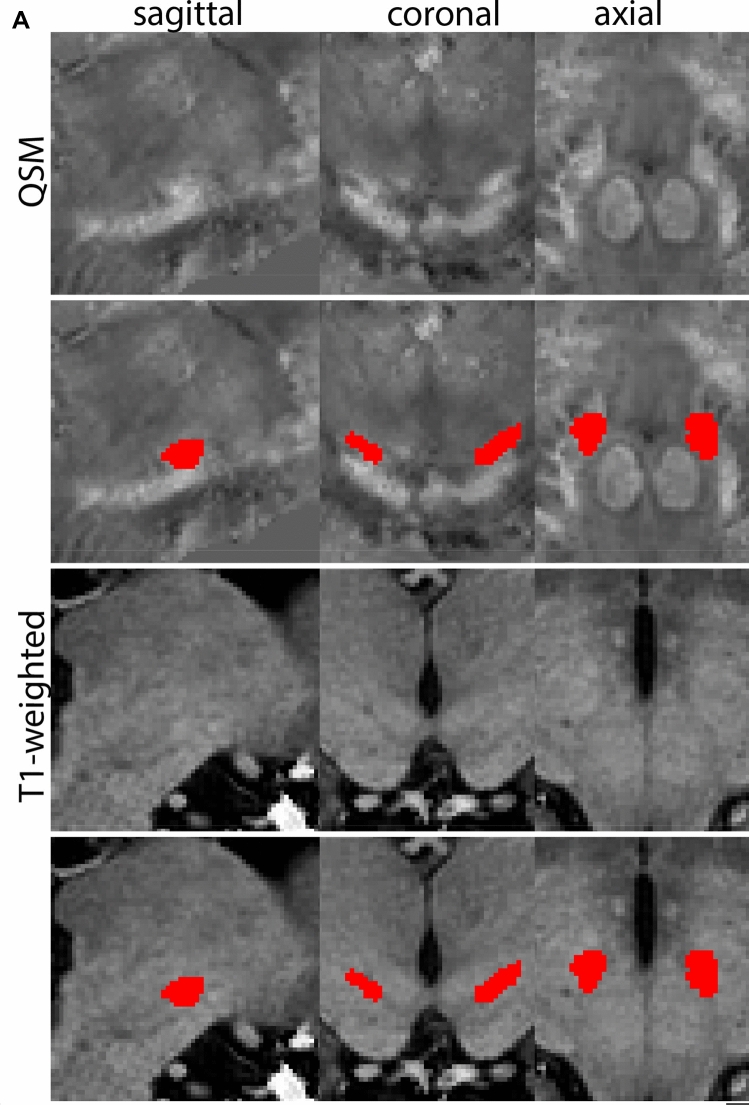

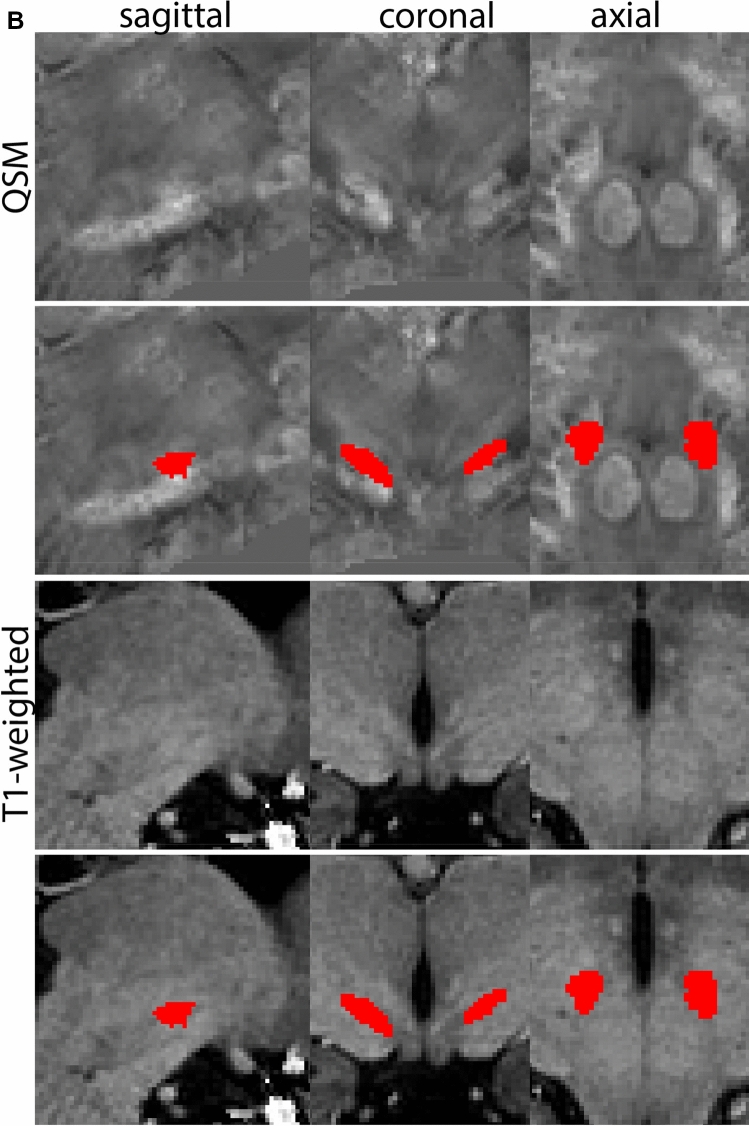

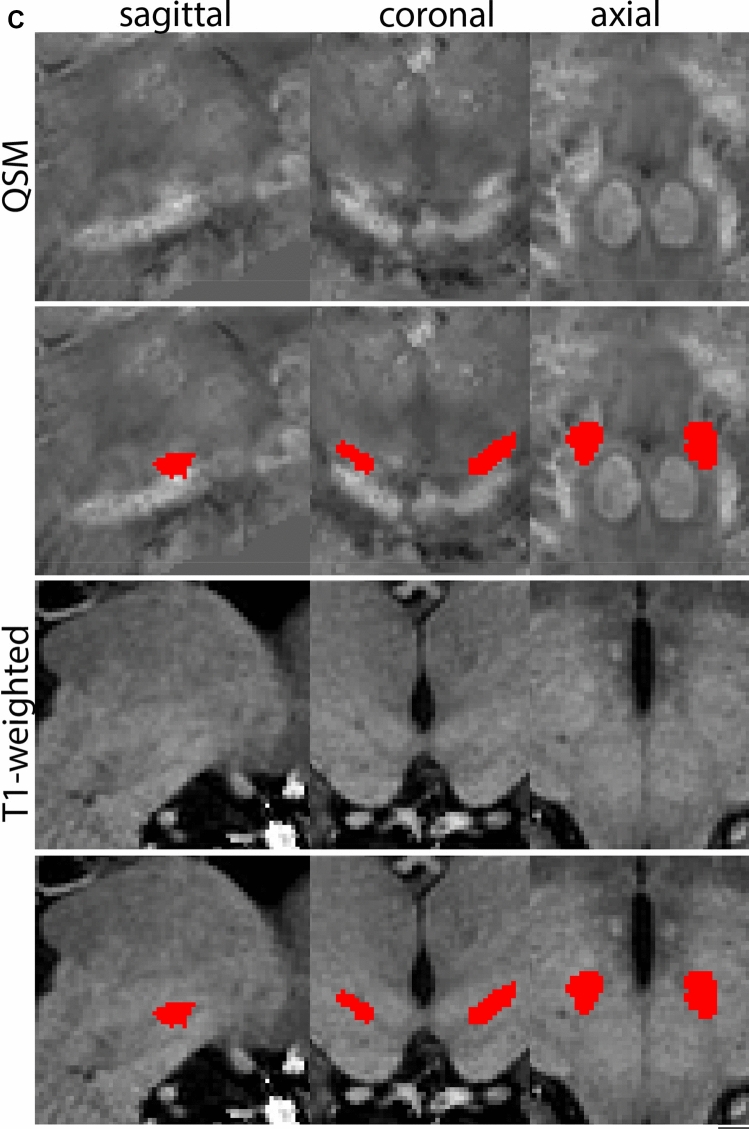
**a** The subthalamic nucleus (STN, red) appears as a hyperintense structure in the QSM contrasts due to its high-iron content. At these levels, the substantia nigra (SN) and red nucleus (RN) also appear hyperintense in the QSM contrasts, and can be used for anatomical orientation. Note the increased visibility of the STN/SN border in the coronal view. Additionally, note the limited visibility of these structures in the T1-weighted contrast. **b** More rostral view of the STN at the level of the mamillary bodies (visible in the T1-weighted contrast). Note the slightly more medial position of the STN at this coronal level. **c** Caudal level of the STN. Note the slightly more lateral position of the STN in the coronal view

The QSM contrast is used for the parcellation of the STN. First, the RN is identified for anatomical orientation in the coronal plane. The RN is located medial to the inferior region of the putamen close to the midline of the brain. At levels where the RN is visible, the SN typically curves around its inferior and lateral edge, with the ventral tegmental area (VTA) located between the SN and the RN. At more caudal levels, the GP disappears. The view is set in a rostral position at which the STN appears at the superior medial edge of the SN in the coronal plane as presented in Fig. 4. Following the structure in rostral direction, the STN increases in size, and the border to the SN becomes increasingly clear. The RN decreases in size at rostral levels, levels at which the STN shows a characteristic ellipsoid shape. Parcellations are started at the center of the STN in the coronal view, where the visibility of the STN/SN border is high. At levels where the location of the STN/SN border cannot be discerned, the delineation is first performed in consecutive slices, and the missing slice will be completed at a later stage when more information from adjacent slides is available. Delineations are continued in the caudal direction. Again, all three viewing planes are used to parcellate, dependent on the visibility of the individual structures. Note that visibility in the different planes can vary across individuals. Posterior to the starting point, the RN is visible medially, as the STN decreases in size. At this point, it can become more difficult to determine the borders of the STN, and all three planes of view should be consulted. Continue in posterior direction and delineate the STN until the RN reaches its maximum diameter. Around this level, the STN disappears. The size of the shape is then confirmed at all levels, and missing voxels are parcellated.

The same procedure is followed in the anterior direction, parcellating on all the slices in which the STN is visible. At more rostral levels, the ic borders the STN laterally. Further rostral, the mamillary bodies (MB) will appear as hyperintense structures in the QSM images. The MB can appear as two individual rounded structures, or as a single structure (due to partial voluming effects). At these levels, the STN and SN decrease in size. The coronal and axial view generally provide the best contrast between the STN and its surrounding structures. Move in anterior direction and parcellate until the STN and SN disappear, typically the MB will be visible at these levels. After completion of the parcellation at the rostral extent, the STN shape is checked and remaining parts are completed. The STN parcellation is then checked in all view planes to confirm consistency. Figure 4 illustrates the parcellation results at a number of anatomical levels across the STN.

### Substantia Nigra

**Fig. 5:**
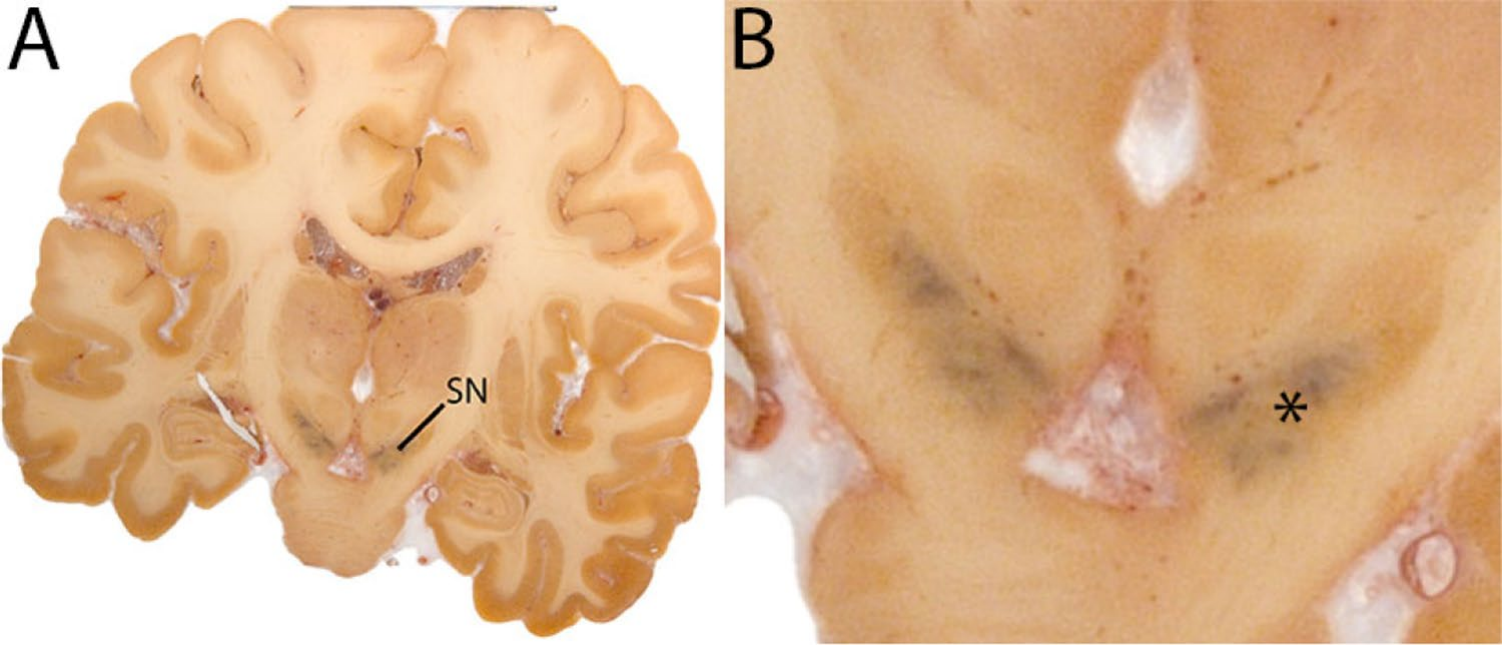
**A** Substantia nigra in a central coronal view. Note location of the SN on the cerebral peduncle. **B** Magnification, asterisk indicates the SN. Note the dark colored neuromelanin

The SN is a curvilinear structure (Fig. 5) and is divided into the substantia nigra pars compacta (SNp) and substantia nigra pars recticulata (SNr). Due to a lack of MRI contrast between the SNp and SNr, the SN is parcellated as a single structure. The SN is located inferiomedial to the globus pallidus (GP), and lateral to the mamillary bodies (MB). The inferior tip of the SN can typically be observed at the level of the MB in the coronal view, with the lateral tip extending slightly above the MB. The SN is oriented at an oblique angle, with its superior tip further lateral to the MB as compared to the inferior extent.

#### Parcellation

The SN is parcellated using the QSM contrast. The MB are used for anatomical orientation. At the level of the MB, the GPe, GPi, and putamen will be visible. The MB are located anterior to the SN, to locate the SN, move towards a more caudal position. When moving in caudal direction through the coronal slices, the STN shifts lateral, and the lateral part extends beyond the lateral tip of the SN. These levels generally provide a better SN/STN separation and provide the starting point for the delineation. All visible borders of the SN are delineated while moving in a more rostral direction. If possible, continue to segment the superior medial border between the SN and STN using the coronal view. The sagittal view can be consulted if the coronal view does not provide the desired SN/STN contrast. If the border is not clearly discernable in these views, it will be identified at a later stage. After disappearance of the SN in rostral slices, move in posterior direction from the starting point. Continue to delineate the SN. Consult all three planes, depending on which displays the border that is delineated best. This will vary between individuals. At levels more caudal to the start point, as the RN increases in size, the SN will decrease in size.

**Fig. 6:**
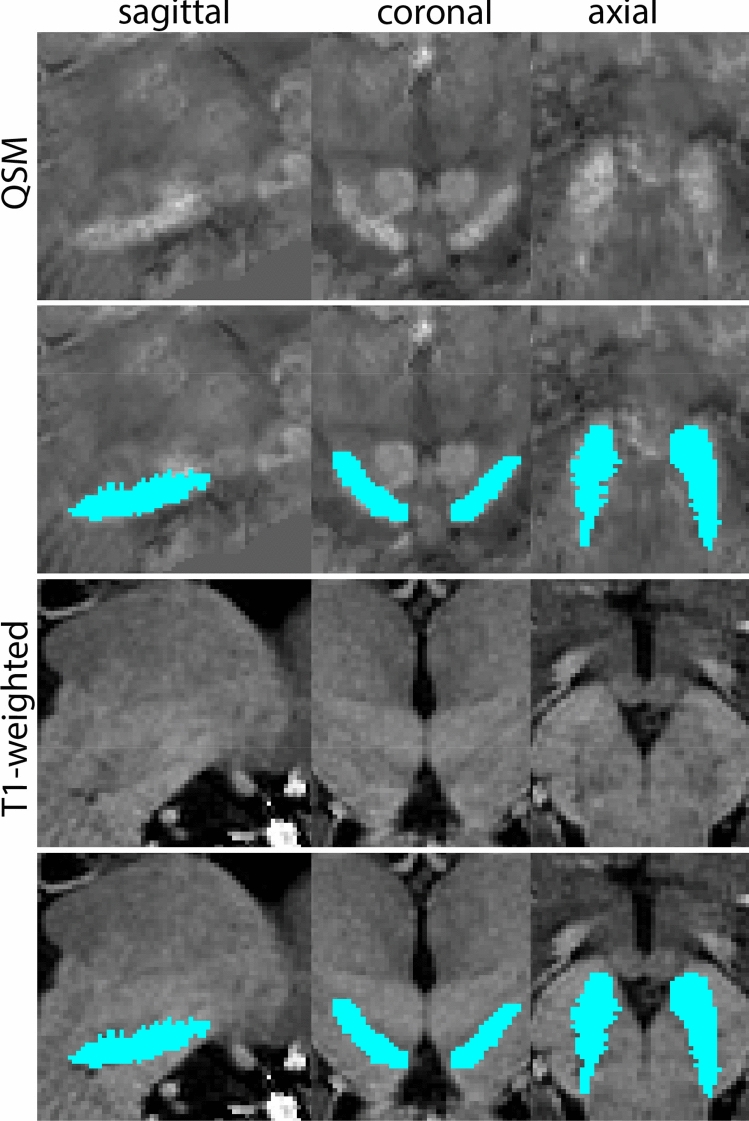

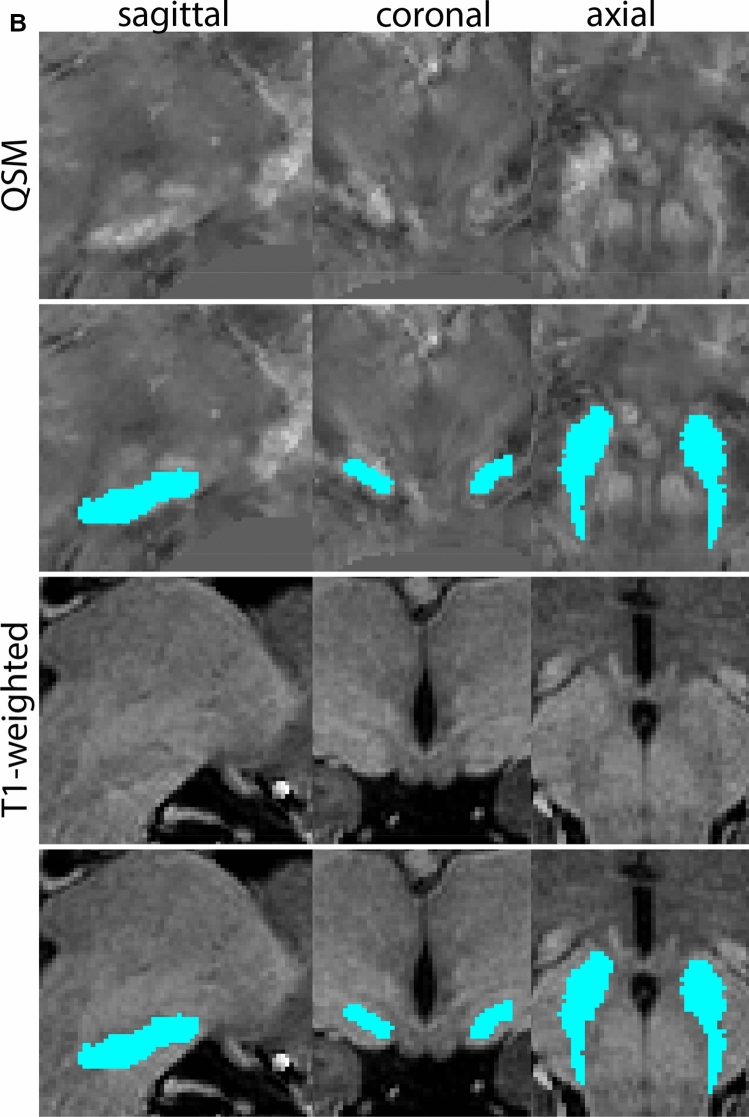

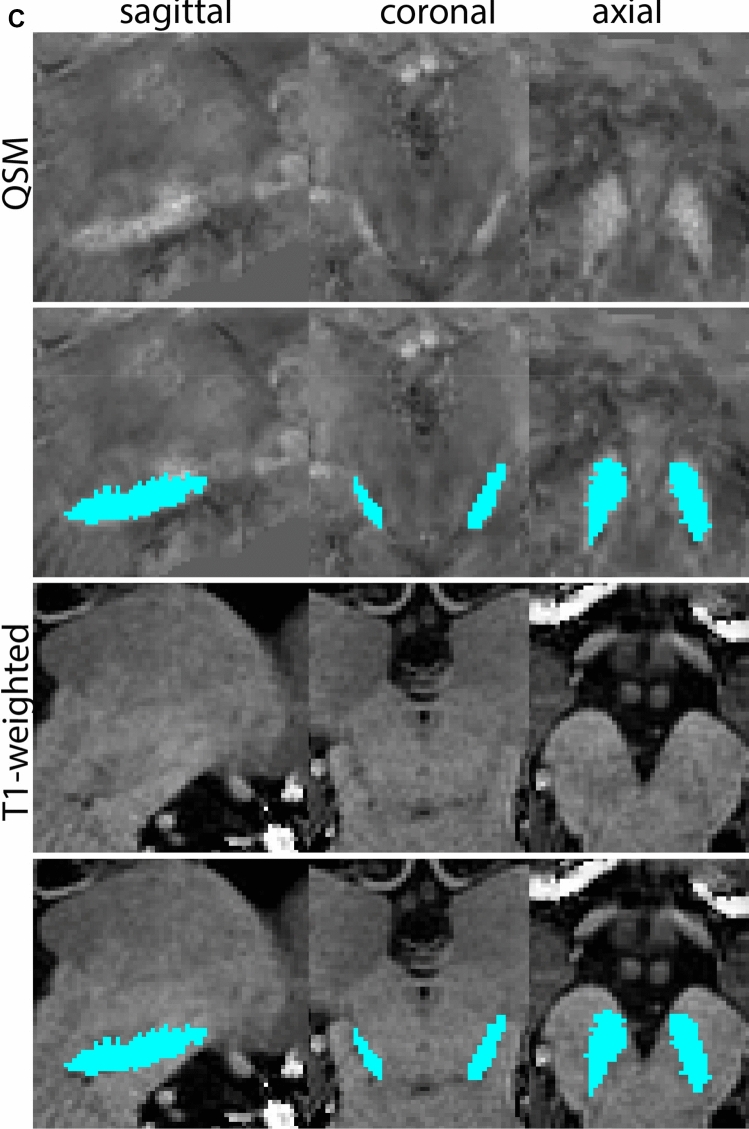
**a** The substantia nigra (SN) appears as a hyperintense structure on the QSM contrast as a result of its high iron content. In coronal levels, the medial part of the SN lies inferior to the lateral parts of the SN. The RN provides a clear anatomical reference point. Note the limited visibility of the SN on the T1-weighted contrast. **b** The substantia nigra (SN) decreases in size in more rostral levels. Note the clear visibility of the mamillary bodies at these levels in the T1-weighted contrast. **c** The substantia nigra (SN) decreases in size and appears in a larger angle in more caudal levels

At the level where the RN reaches its maximum diameter, the STN has typically disappeared. At this point, towards the posterior extent of the SN, some of the voxels within the SN can show lower intensity, seeming splitting the SN. In the axial view this may resemble a swallow tail. The hypointensity has been attributed to nigrosome 1 (Blazejewska et al. 2013). These hypointense voxels are included as part of the SN. Delineation in adjacent slices will help resolve remaining borders. Delineation is then continued in the caudal direction, and the SN and RN will decrease in size. Continue to move caudal until the RN disappears. At this point, a small part of the posterior region of the SN will still be visible. More caudally the SN will disappear. Delineation of the SN is then completed. Figure 6 illustrated the parcellation results at various levels in the SN.

### Red nucleus

**Fig. 7:**
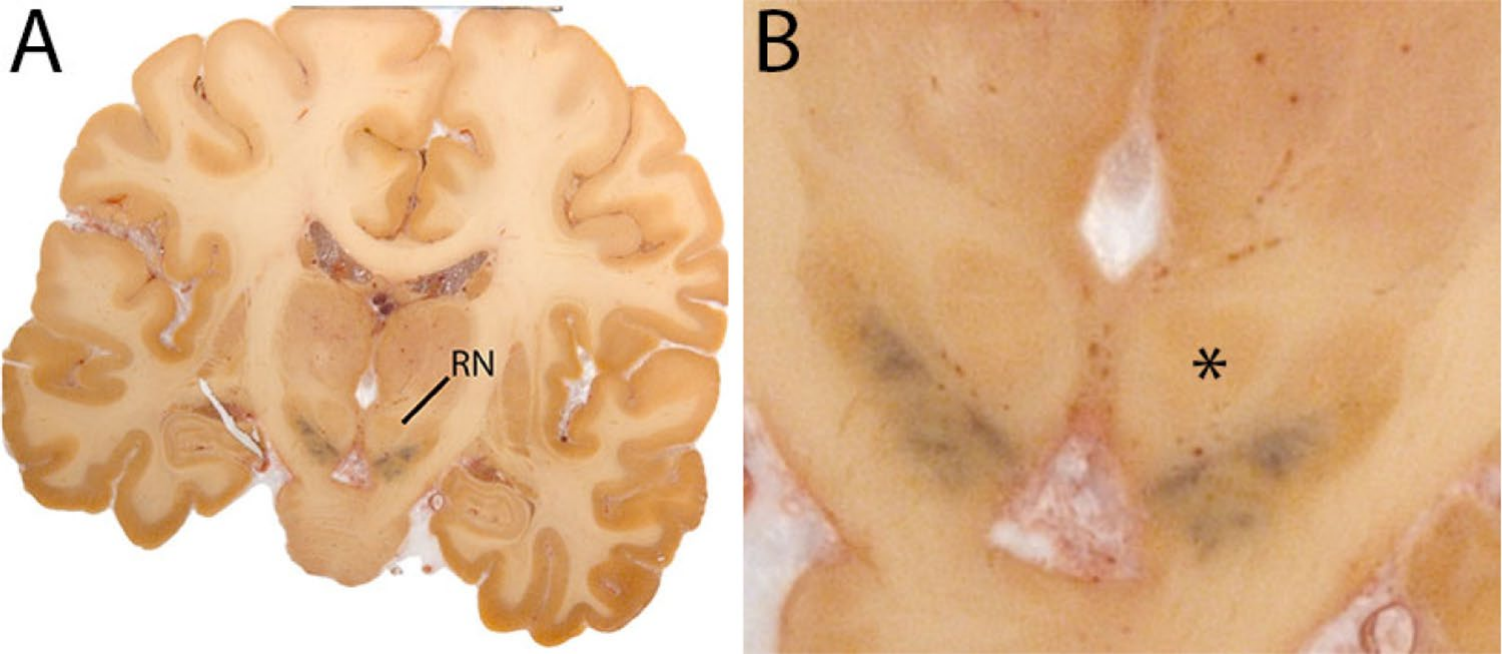
**A** Red nucleus (RN) in a central coronal view. **B** Magnification, asterisk indicates the RN. Note the typical rounded shape of the RN

The RN is an iron-rich nucleus in the tegmentum of the rostral midbrain. It has a distinct oval-shape and is located close to the midline of the brain. A clear landmark for the location of the RN is the SN. In the sagittal plane, the RN and SN can be roughly located inferior to the Tha and superior to the pons. In the coronal plane, the RN lies medial to the STN and SN, and inferior to the third ventricle (3V). The RN consists of a more caudal magnocellular part and a more rostral parvocellular part that relay functionally different motor fibers systems. On MRI, these segments cannot be distinguished. Therefore, the RN is parcellated as a single structure (Fig. 7).

#### Parcellation

**Fig. 8:**
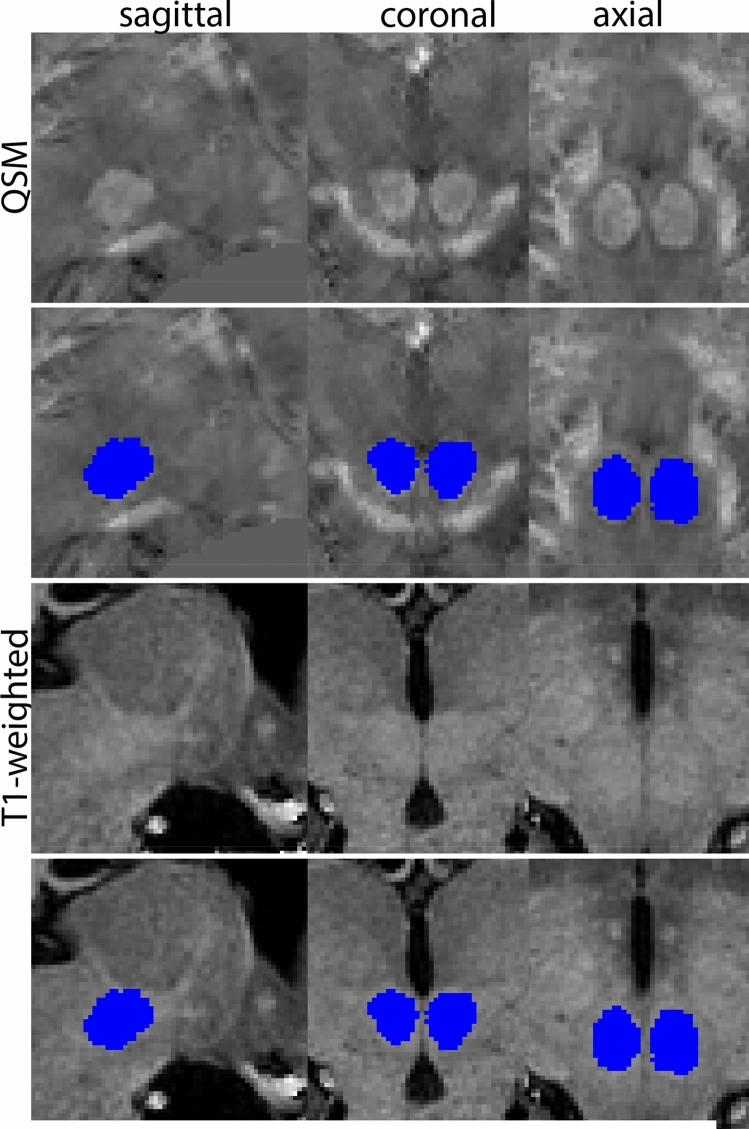

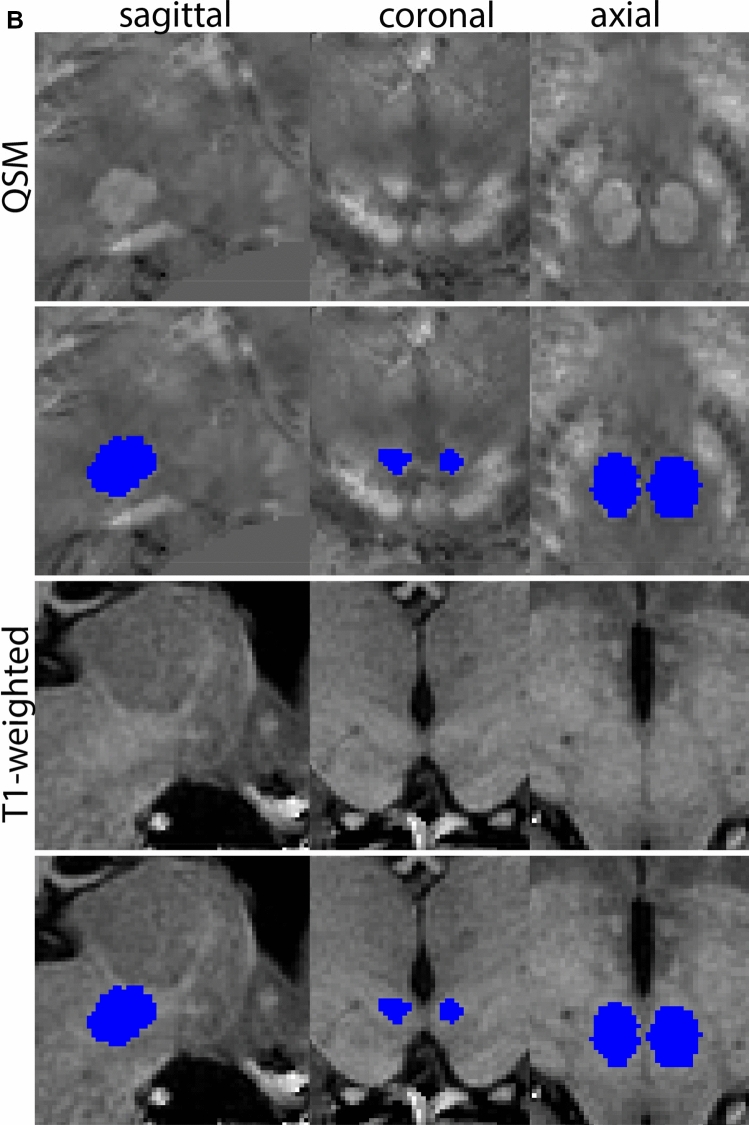

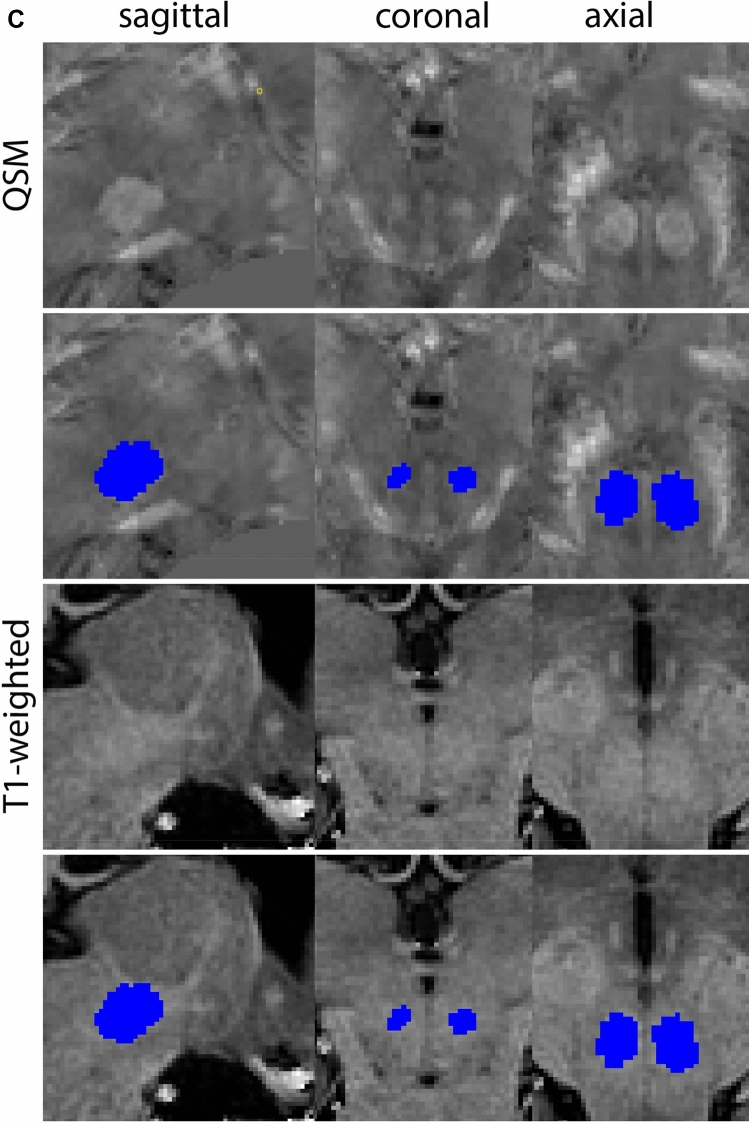
**a** The red nucleus (RN) at a central level is clearly visible as a rounded structure in all three viewing planes in the QSM contrast. Note the limited visibility of the RN in the T1-weighted contrast. **b** The red nucleus (RN) at a rostral level can still clearly be identified as is decreased in size. Note the limited visibility of the RN in the T1-weighted contrast. **c** The red nucleus (RN) at a caudal level can still clearly be identified as is decreased in size. Note the limited visibility of the RN in the T1-weighted contrast

The RN is identified as a hyperintense structure in the coronal view on the QSM contrast. Moving from rostral to caudal, the RN will appear after disappearance of the putamen and pallidum and emergence of the SN and STN. The STN will decrease in size and eventually disappear as the RN and SN increase and their relative distance will become smaller. After the RN reaches maximum diameter, the decussating fibers from the superior cerebral peduncles will appear inferomedial to the RN. From there, the RN will decrease in size until it disappears typically around the same level as the SN. In the coronal view, parcellation can pose challenges at the ventrolateral and inferomedial borders, and on sagittal views, the RN can appear somewhat triangular, showing more interference from crossing fibers tracts. Typically, the other planes provide additional information which allows detailed parcellation of the RN. The 3D mask should be generally ovoid and not have any significant abnormalities such as large protrusions or indentations on the surface. Delineation results of the RN are illustrated at various anatomical levels in Fig. 8.

### Amygdala

**Fig. 9:**
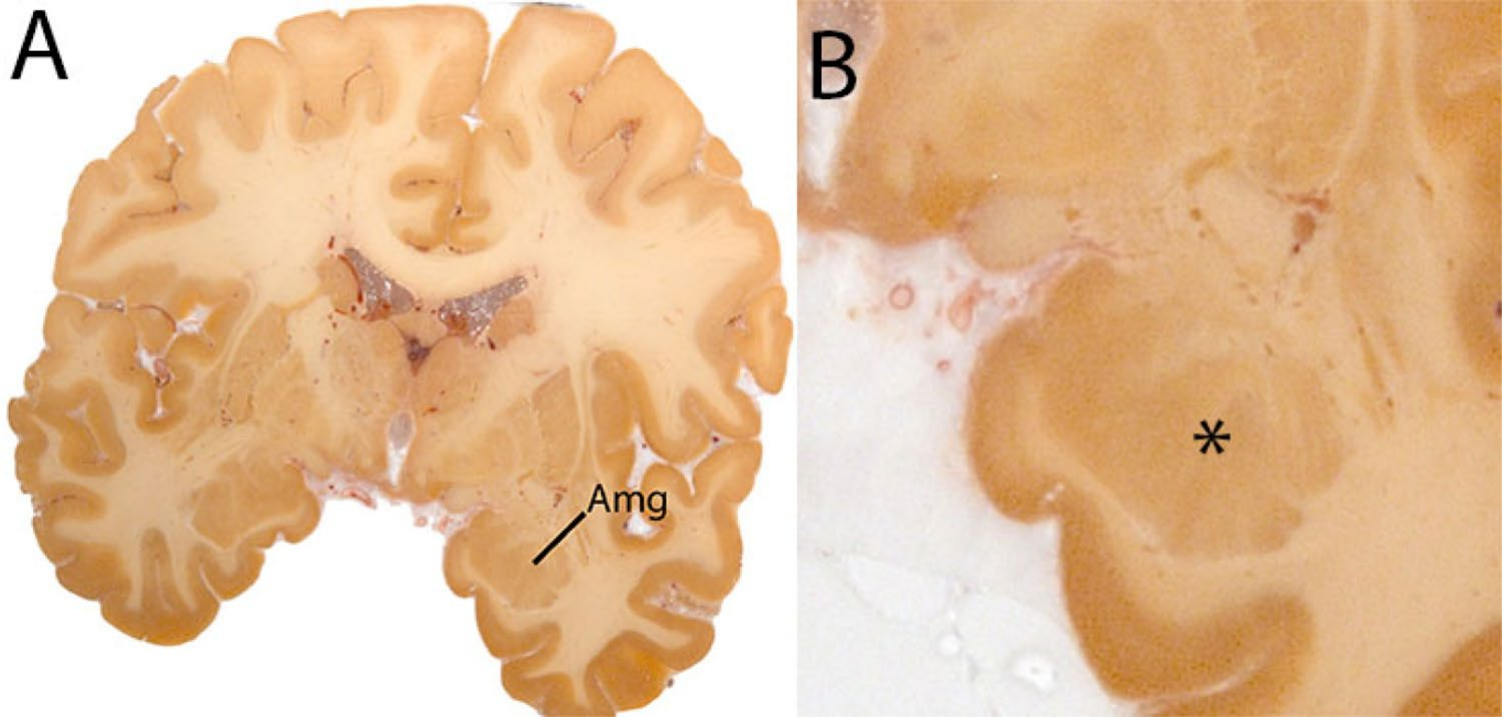
**A** The amygdala (Amg) in a central coronal view. **B** Magnification, the asterisk indicates the location of the Amg

The amygdala (Amg) is a large structure that lies deep within the temporal lobe, anterosuperior to the hippocampus. Two layers of nuclei are distinguished; the deep and superficial layer. The deep layer includes the basolateral, basomedial, and lateral nuclei. The superficial layer is allocortex, phylogenetically older and has been highly conserved. It is composed of the anterior and posterior cortical nuclei, medial nucleus, and the periamygdaloid cortex. Additional areas belonging to the amygdala include the following: the anterior amygdaloid area, the amygdalo-striatal area and the intercalated cell area (deCampo and Fudge 2012). The Amg is delineated as a single structure (Fig. 9).

#### Parcellation

Parcellation of the Amg is started at ventral levels of the structure on the T1-weighted contrasts. The hippocampus, the temporal horn of the lateral ventricle (LV) and surrounding white matter (WM) are used as anatomical landmarks. Delineations are started at the most inferior slice of the Amg in the axial plane, to allow identification of the inferior border the coronal and sagittal planes. The most inferior slice is the slice in which both the LV and the V-shaped WM are visible. The temporal horn of the LV forms the posterior border of the Amg. The other borders are defined by surrounding WM. The Amg appears as an ovoid shape in the axial plane. Delineation is performed in the axial plane for five slices. While moving up, the WM boundaries will become more difficult to discern. At more superior levels, no WM separation between the subiculum and Amg will be present. At this point use the remaining WM to determine the visible borders. The Amg is then delineated in the sagittal view. First the most lateral slice containing the Amg is identified. The Amg is located between WM anterior to the hippocampus and the temporal horn of the LV. Note that the Amg can be crossed by a thin layer of WM in this view. The Amg is parcellated in the five most lateral slides, and increases in size as parcellation moves medially.

Parcellation is then continued in the coronal view. First, the most caudal slice is identified using the optic tract, the temporal horn of the LV, the striatum and hippocampus as landmarks. The ventral border is already parcellated. Subsequently, locate the posterior Amg border using the coronal and the sagittal view.

In the coronal view the Amg is located at the same height and lateral to the optic tract, inferior to the AMY are the stria terminalis and the hippocampus, superior and lateral WM demarcate the border of the Amg. In the most coronal slices, the Amg will appear small, and will increase in size rapidly. In some cases, the Amg will appear around the hippocampus. At these levels, the dorsal and dorsolateral border of the Amg are demarcated by WM and the putamen. Since the border between the ventral part of the Amg and the hippocampus is not easily discernable in the coronal plane, the parcellations are continued in the axial view. As the hippocampus starts to disappear it will become easier to discern the Amg. The Amg is bordered dorsomedially by the optic tract (ot), entorhinal sulcus (ES), and ventromedially by the entorhinal cortex (EC). The ventrolateral border is formed by the temporal horn of the LV, which can be thin at these levels, and axial and sagittal images should be used for guidance. Dorsolaterally, WM borders the Amg. Parcellations should not include the putamen. The shape of the Amg becomes more rectangular at this point. The structure then remains stable for a number of slices at which the ES disappears and the periamygdaloid cortex (PAC) starts to appear bordering the Amg at the dorsomedial extent. Axial views can be used for additional guidance. In most rostral slices, the PAC and EC will border the Amg, and the axial and sagittal views are used to assist the identification of the Amg borders. After the disappearance of the Amg, the structure is checked for consistency in all three planes.

**Fig. 10:**
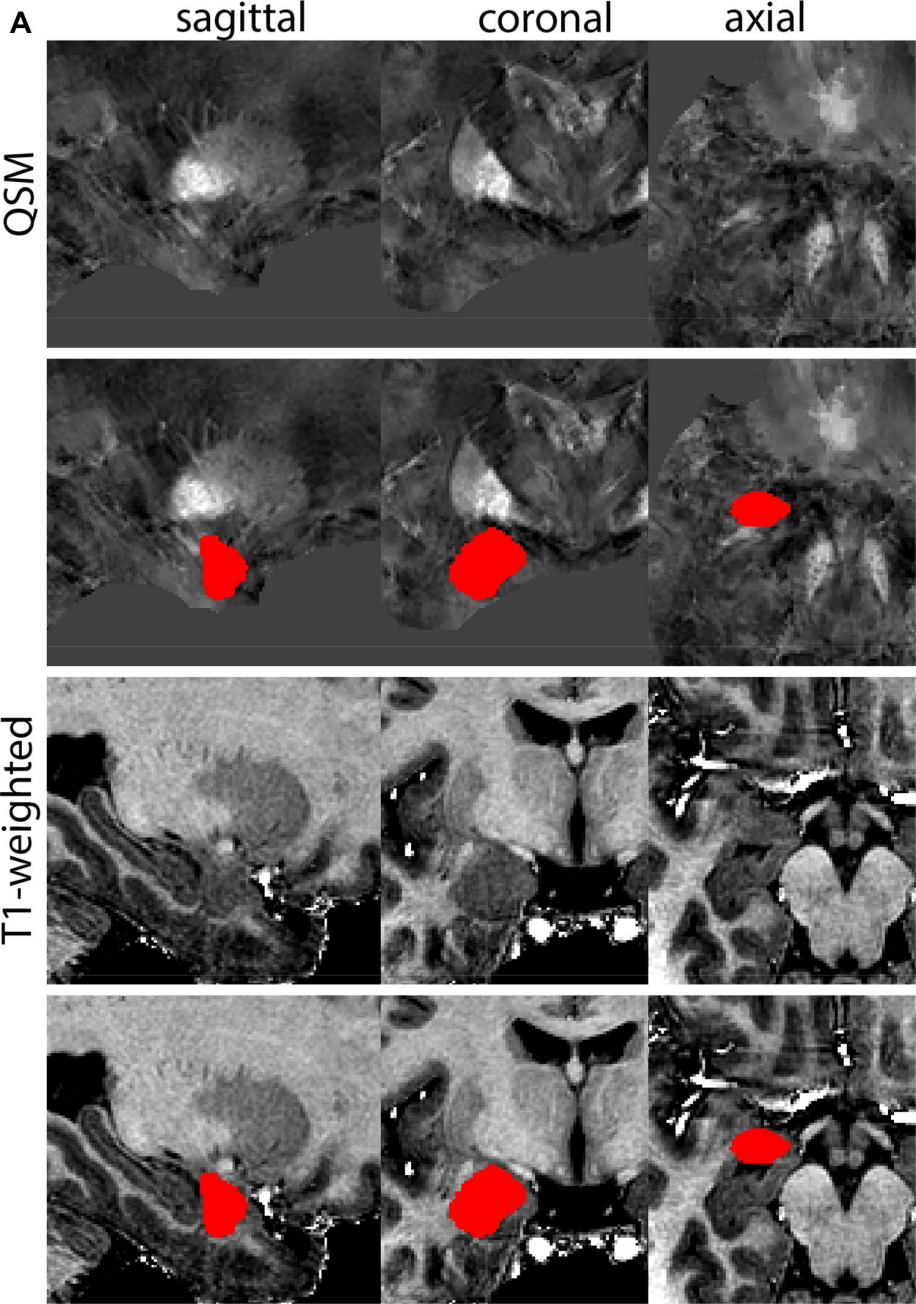

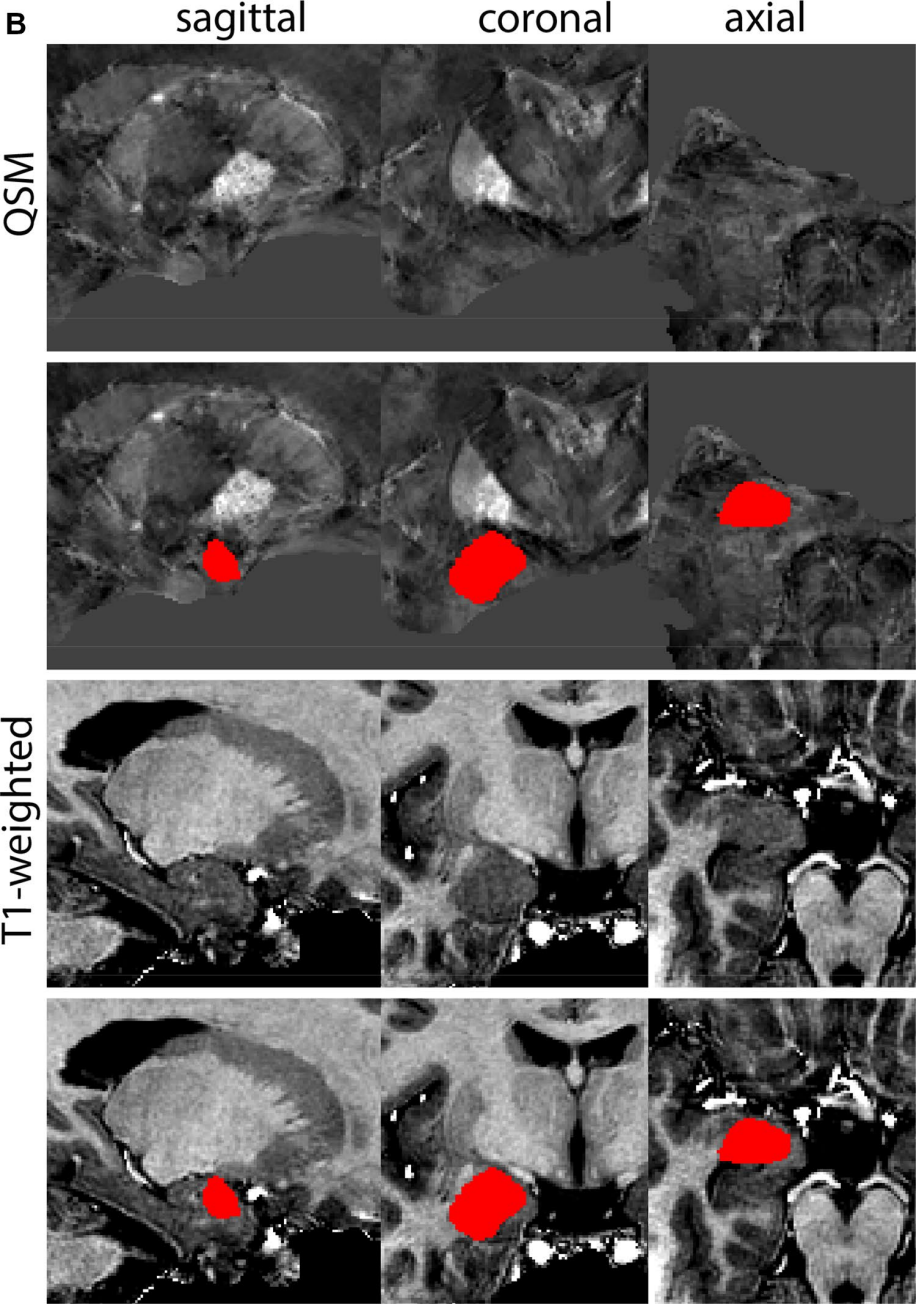

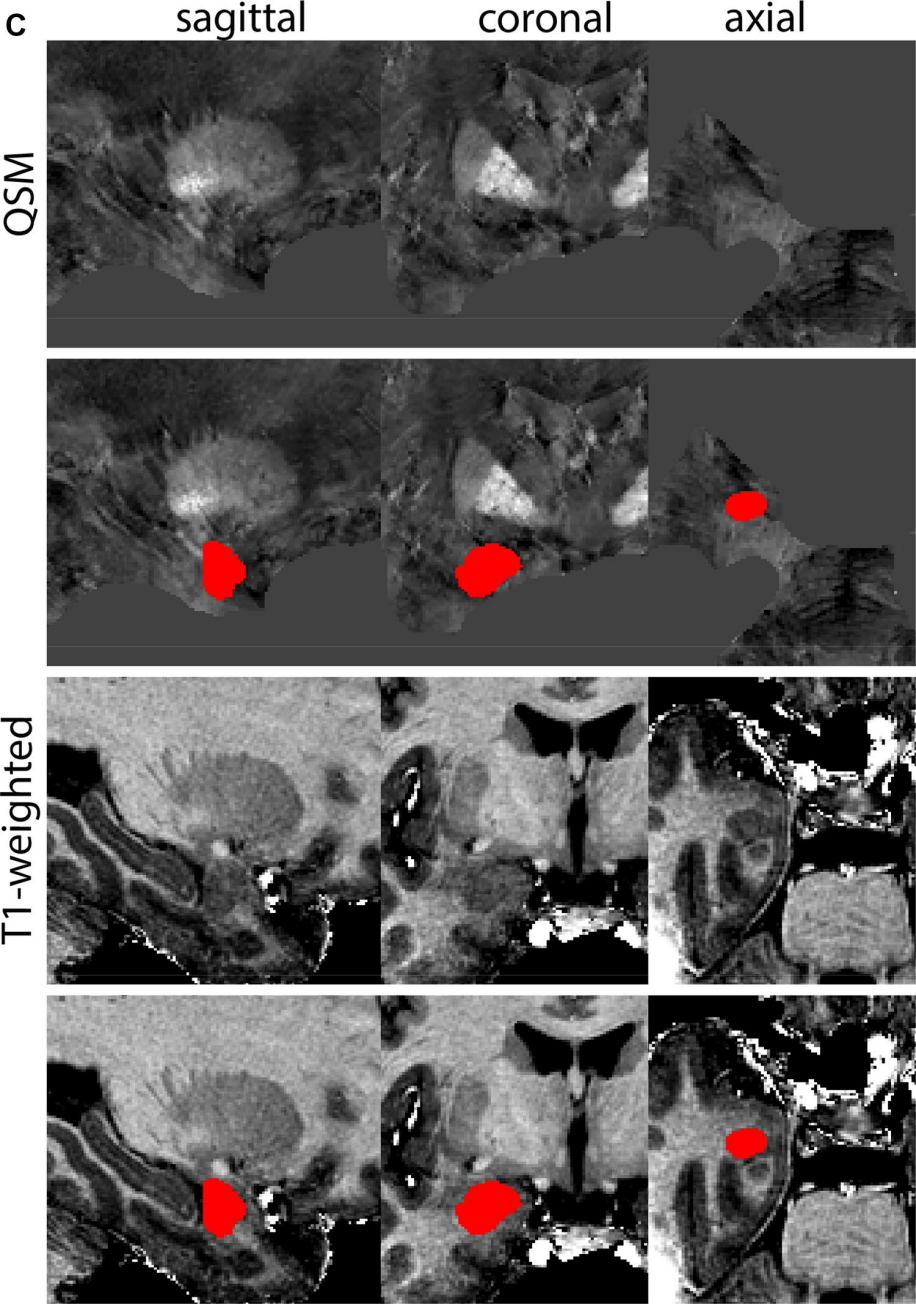
**a** The Amygdala (AMG) is a larger structure in the inferomedial temporal lobe. Note the clipping in the QSM images. **b** The Amygdala (AMG) is a larger structure in the inferomedial temporal lobe at more inferior levels (axial view). Note the clipping in the QSM images. **c** The Amygdala (AMG) is a larger structure in the inferomedial temporal lobe at the most inferior levels (axial view). Note the clipping in the QSM images

The Amg borders other gray matter structures, without a (visible) layer of white matter to discern the grey matter structures. This is the case in the superior part of the amygdala at the level of the central nucleus. The Amg closely borders the globus pallidus, and in caudal coronal slices the Amg is close to the ventral putamen, with only a small strand of white matter to separate them, which can be difficult to discern due to partial voluming. If parcellations of the globus pallidus and the striatum are available, these can be used for reference. Delineation results of the Amg are illustrated at various anatomical levels in Fig. 10.

### Claustrum

**Fig. 11:**
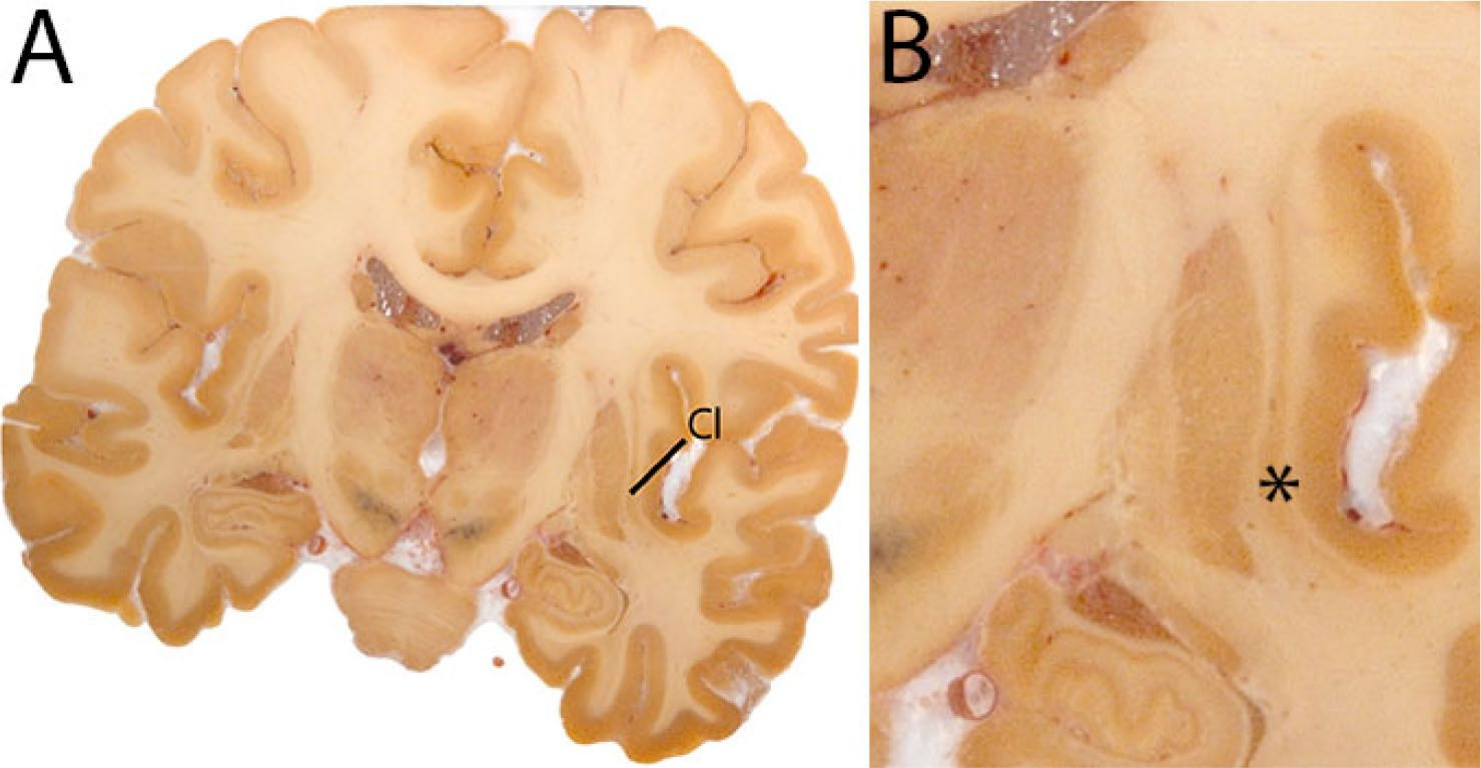
**A** The claustrum (Cl) shown in a central coronal view, is a thin grey matter structure between the external and extreme capsule. **B** Magnification, the astersisk indicates the location of the Cl

The claustrum (Cl) is a thin gray matter sheet structure, located medial to the insula, and is bordered by the extreme capsule, and the external capsule. Rostrally, the Cl can appear anterior to the internal capsule. The caudal end of the Cl is approximately at the central level of the RN (Fig. 11).

#### Parcellation

The Cl is bordered by WM tracts. The surroundings of the Cl will be hypointense, whereas the Cl itself will be relatively hyperintense in T1-weighted contrasts. The Cl can be discerned between the putamen and the insula in the coronal view. The most efficient way to delineate the Cl is in the axial plane. First, perform the delineations in the superior direction, then continue parcellation in the inferior direction.

**Fig. 12:**
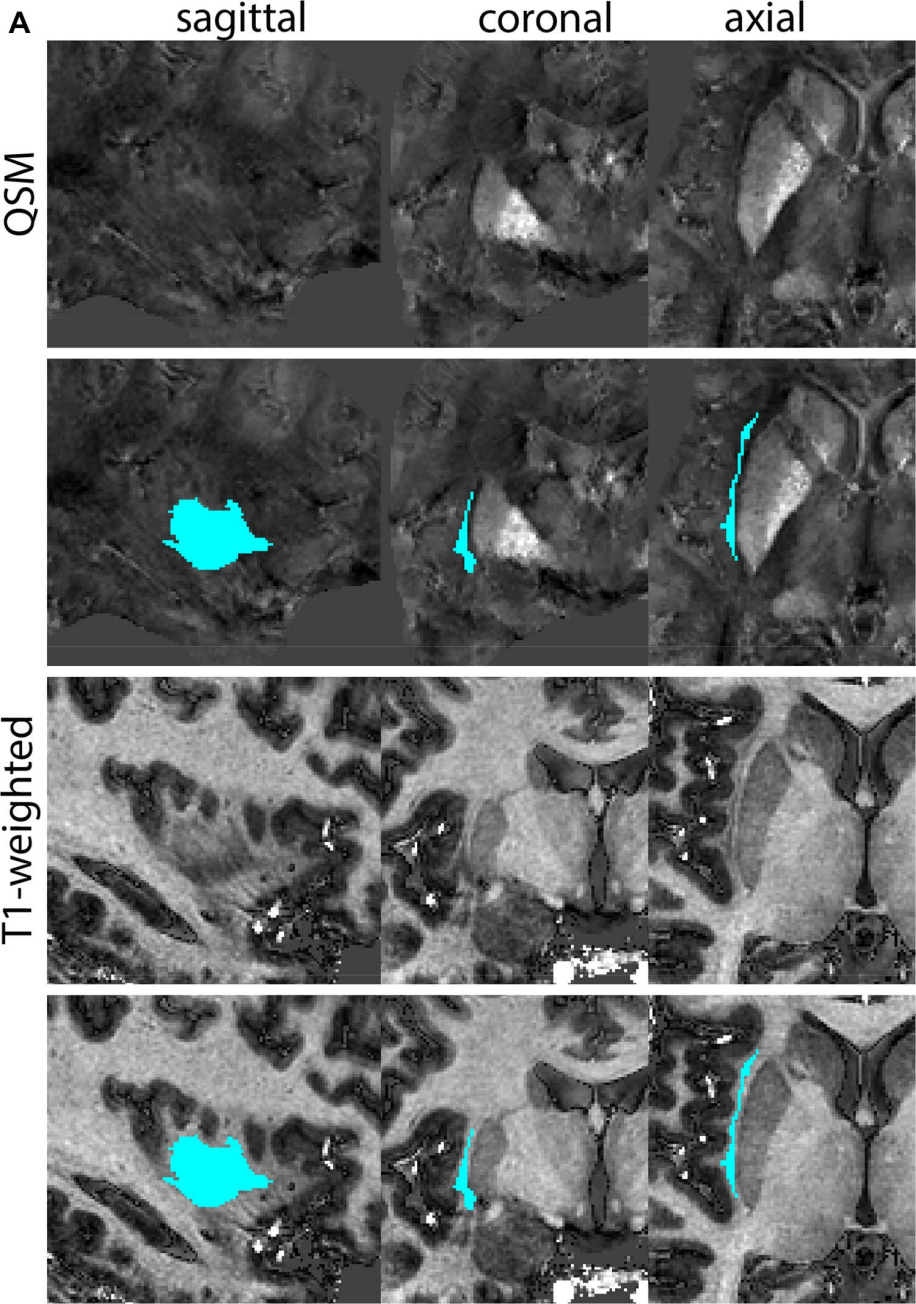

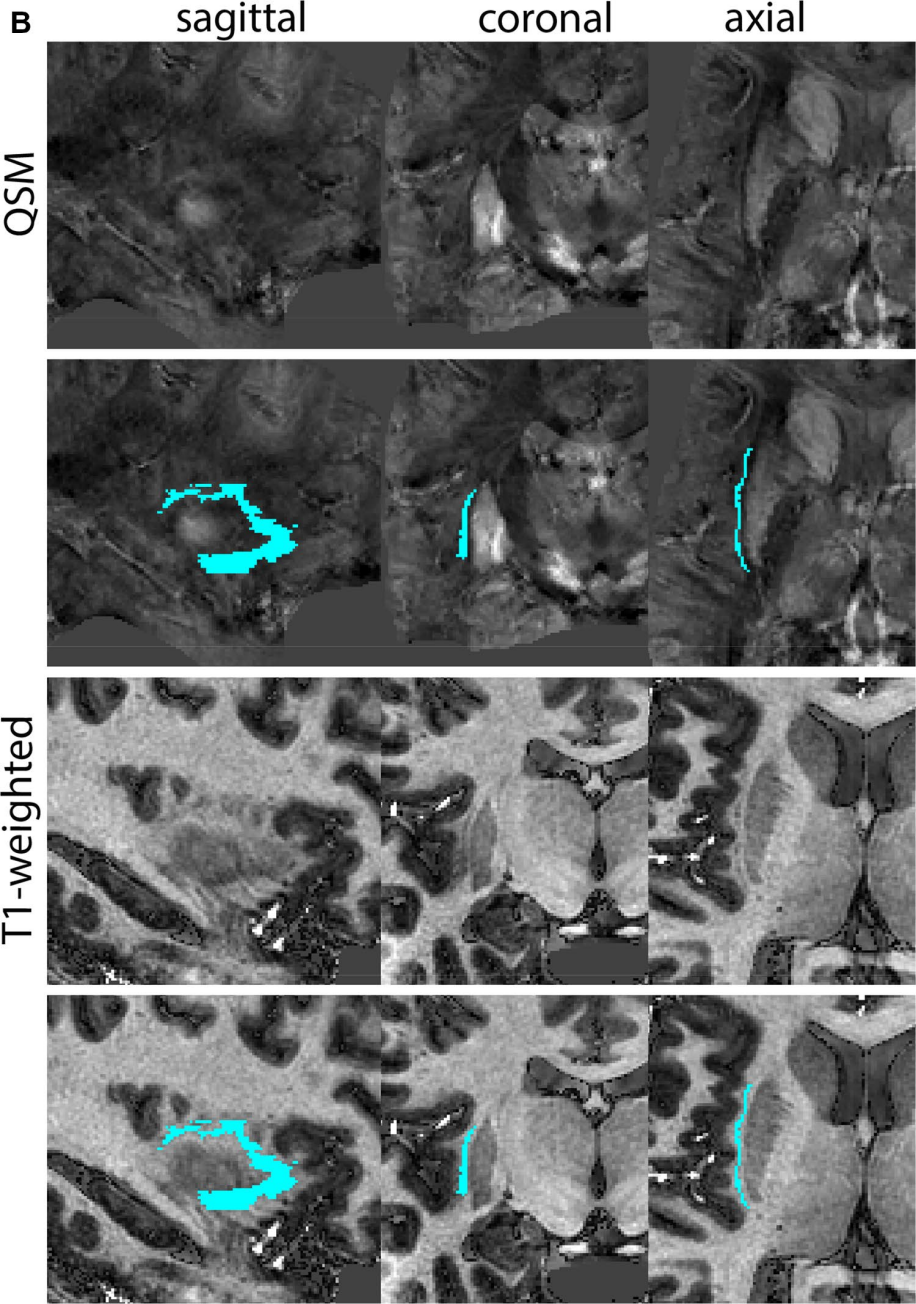
**a** The Claustrum is a thin grey matter sheet located between the external and extreme capsule. **b** The Claustrum is a thin grey matter sheet located between the external and extreme capsule. Note the peculiar appearance in the sagittal view, which results from the folding of the sheet

Since the Cl is a thin sheet of gray matter it is particularly important to check the delineations in the coronal and sagittal plane. Make sure the insula is not included in the parcellations and that the parcellation is continuous. This is particularly clear in the coronal view. Check the 3D structure of the Cl in all planes. The appearance of the claustrum is illustrated at various anatomical levels in Fig. 12.

### Fornix

**Fig. 13:**
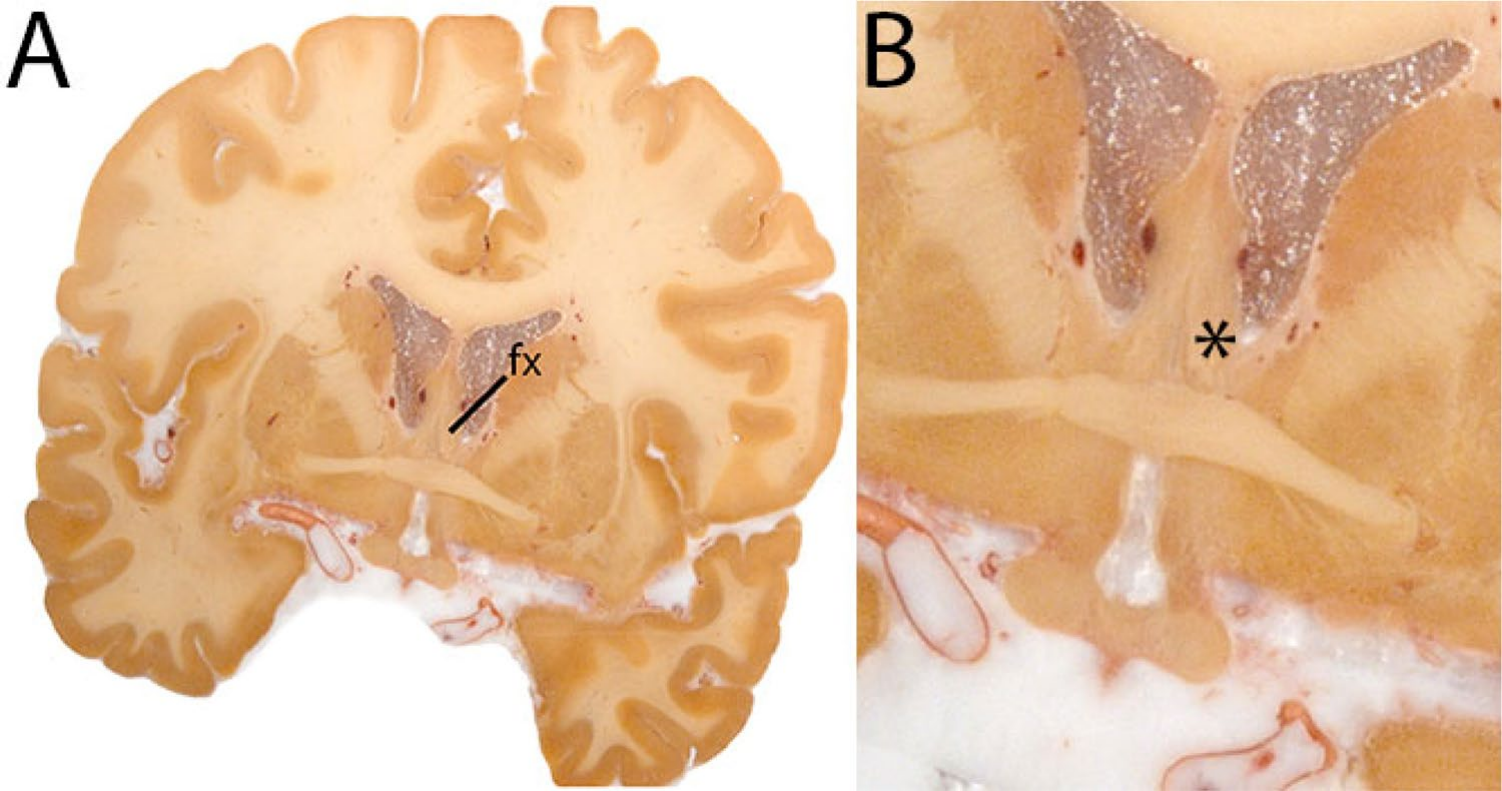
**A** The fornix is a large WM structure that connects the hippocampus and the mamillary bodies (MB), and shown here at the level of the anterior commissure. Due to its curvature, in the hypothalamus it can be identified as the fornical columns that move down to the MB as well as a midline structure located inferior to the corpus callosum. **B** Magnification, asterisk indicates the fornix. Note the red appearance of the septal veins due to remaining red blood cells in the tissue

The fx is a C-shaped white matter structure which connects the hippocampus and the MB. Its fimbriae emerge from the hippocampus, which thicken to form the crura of the fx. The fx curves in the rostral direction inferior to the splenium of the corpus callosum. The crura merge to form the body of the fx, approximately at the level of the hippocampal commissure. The body of the fx then continues to curve in the anterior direction at the level of the midline, inferior to the corpus callosum. Near the level of the foramen of Monro, the fornical body moves down and splits into two columns. The anterior commissure is used as a landmark to demarcate the rostral border of the fornical columns. It is important to note that the precommissural fibers of the fx are not included in this delineation, since they are commonly not visible on our MRI contrasts. The postcommissural fibers which project to the MB are visible and included in the delineations (Fig. 13).

#### Parcellation

The fx appears hyperintense on T1-weighted contrasts. Since the fornix merges at the midline of the brain, it is parcellated as a single structure and not separated for the individual hemispheres. Delineation is performed in the coronal plane, and the sagittal and axial planes are used to confirm the results. Additionally, the sagittal plane is used to confirm the location of the border between the fornix and the septum pellucidum.

The body of the fx is identified in the center of the brain inferior to the body and splenium (caudal extent) of the corpus callosum. The septum pellucidum bridges between the corpus callosum and the fx, but has a less hyperintense signal, which can at least partially be ascribed to partial voluming effects since the septum pellucidum is a small structure surrounded by CSF. At this level, the fx borders the lateral ventricles. Ventrally the fx borders the 3V. Delineation is performed in the coronal plane moving anterior from the starting point. The septum and adjoining gray matter structures are not included in the parcellation. As the fx curves down and its columns form, the axial view is used to confirm the location of the fornical border. The anterior commissure demarks the rostral border of the MRI delineation. At this level the fx forms two columns. From the level of the anterior commissure, the fornical columns curve back to the MB.

After completion of the delineation of the rostral extent of the fx, the body of the fx is delineated in the caudal direction. At the caudal border of the fornical body, the fornix splits into the two crura. The curve of the corpus callosum is followed to determine the border between these two structures. Delineations are continued to include the caudal extent of the fx. The crura of the fx curve and connects to the Tha through afferent and efferent fibers (fimbriae). The fimbriae are included in the delineation, and the grey matter of the Tha is excluded. After completion of the delineation of the fimbriae, the sagittal plane is used to confirm the accuracy of the delineations. The white matter anterior to the curve of the CA3 region of the hippocampus is part of the alveus and should not be included. The grey/white matter contrast is used to distinguish the white matter of the fimbriae from the grey matter of the hippocampus, and delineation is completed.

**Fig. 14:**
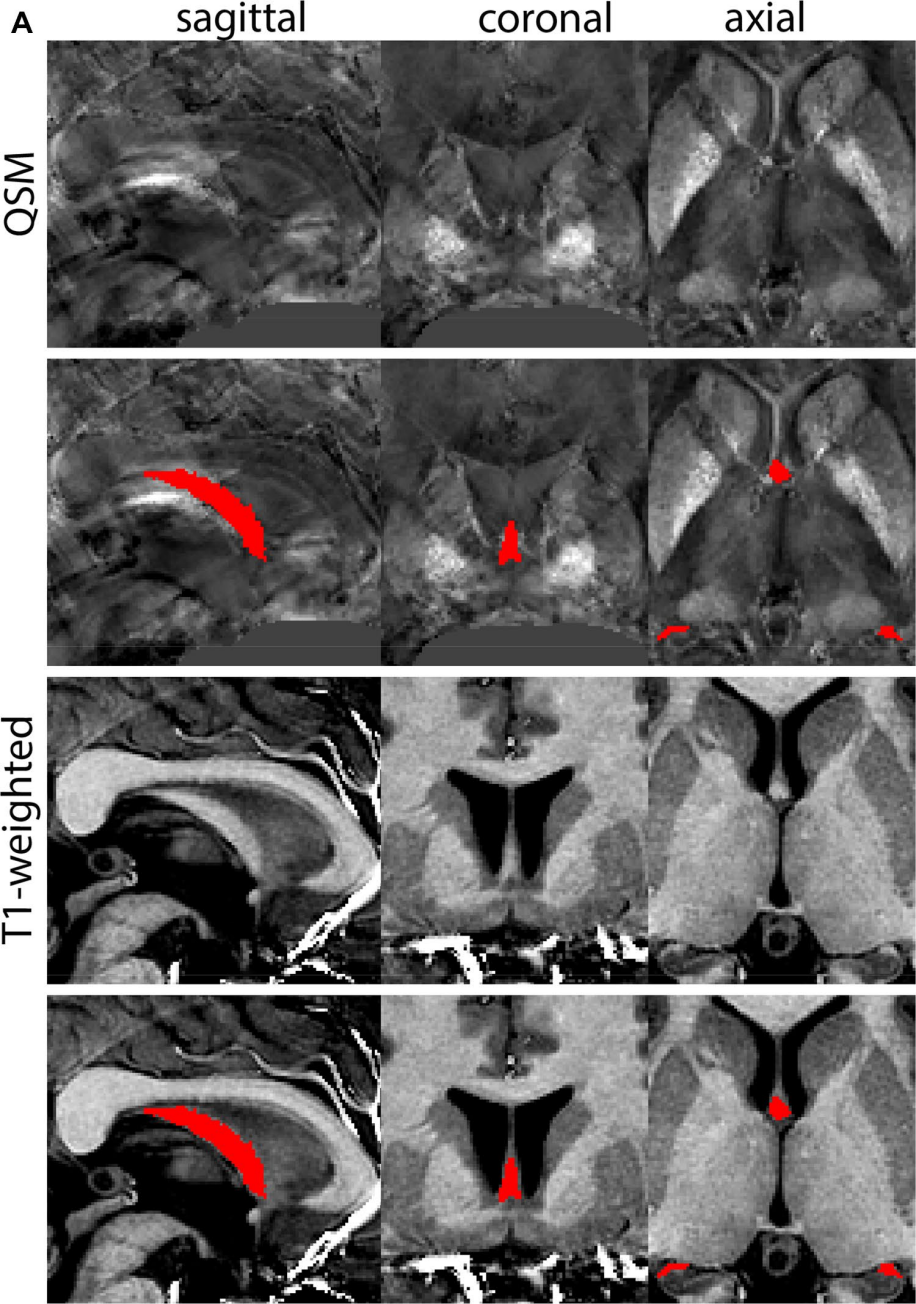

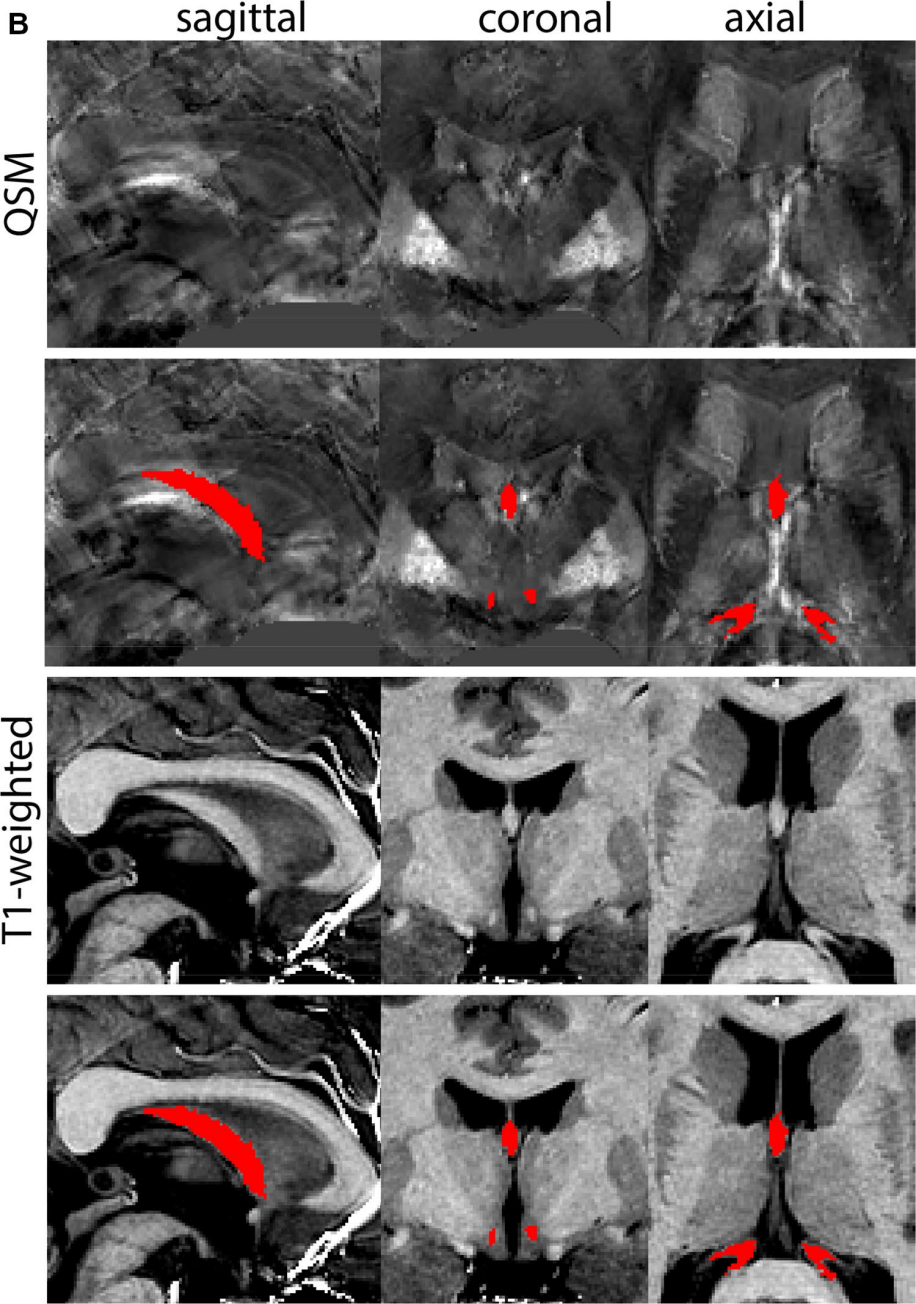

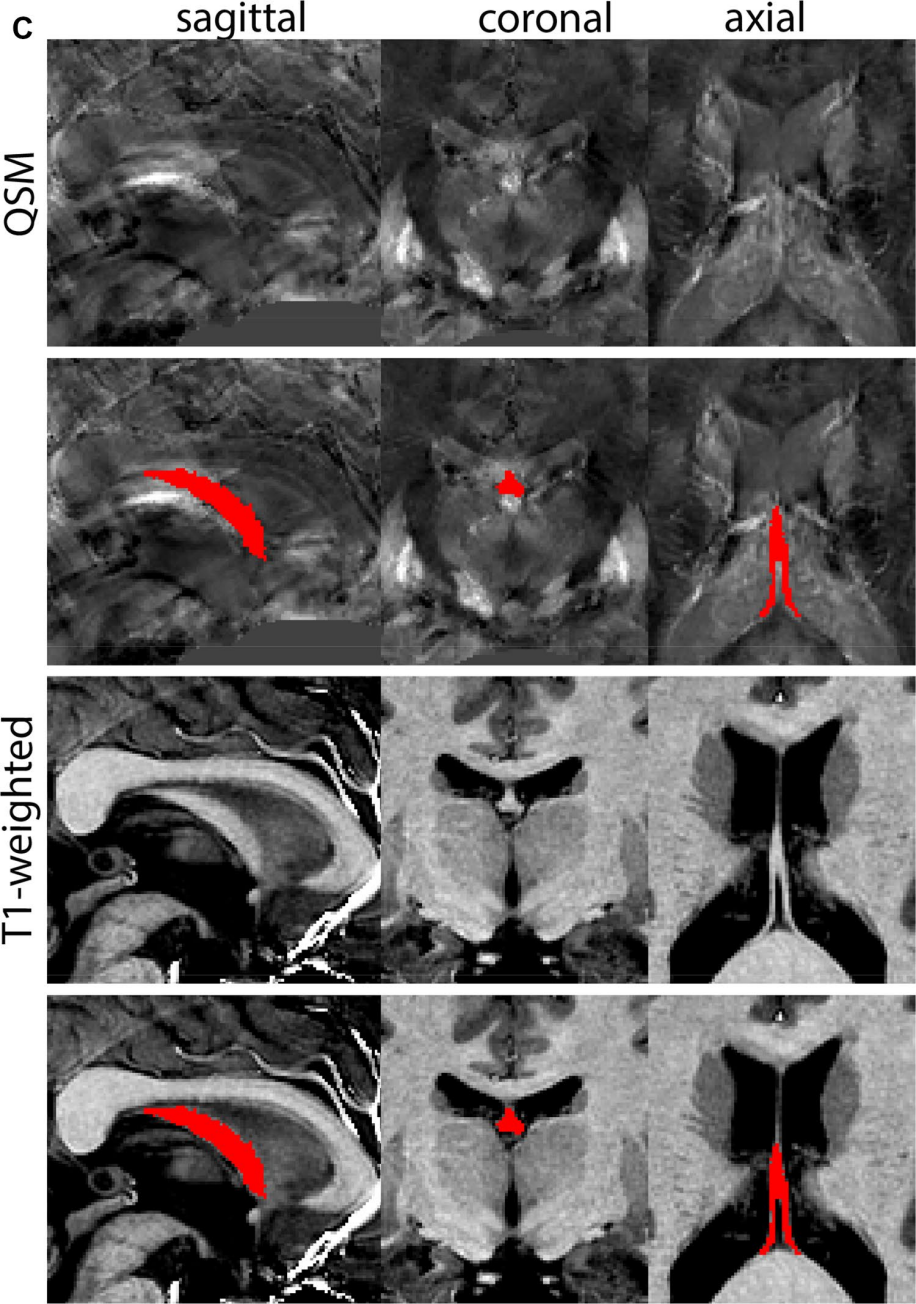
**a** The fornix (fx) is a large C-shaped white matter structure shown here at the level of the anterior commissure. **b** The fornix (fx) is a large C-shaped white matter structure. Note that at the level of the hypothalamus the fx has formed two columns that will connect to the mamillary bodies (MB). **c** The fornix (fx) is a large C-shaped white matter structure. Note the disappearance of the fornical columns posterior to the mamillary bodies

A final check is performed by reviewing all three planes. The fx should appear as bilateral curved structures, which are merged in the middle. At this stage it is confirmed that neither the anterior commissure nor the corpus callosum is included. Delineation results of the fx at various anatomical levels are illustrated in Fig. 14.

### Internal capsule

**Fig. 15:**
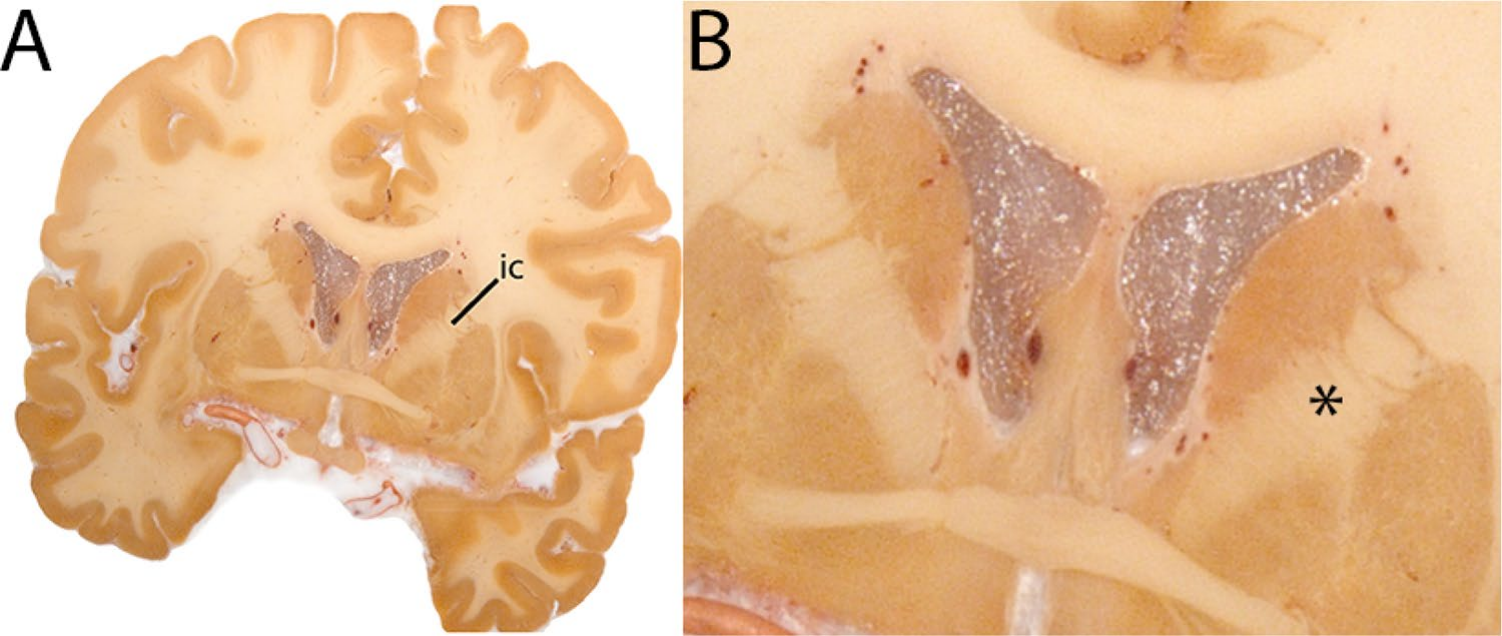
**A** The internal capsule (ic) shown at a central coronal level. **B** Magnification, ic is indicated by the asterisk. Note its white appearance

The ic is a white matter structure that connects the cortex and the basal ganglia. The Str is traversed by the ic, which separates the caudate from the putamen. The ic includes fibers of the anterior thalamic radiation and frontopontine fibers (Chowdhury et al. 2010) (Fig. 15).

#### Parcellation

The T1-weighted contrast is used in combination with the QSM contrast to delineate the ic. The ic is identified in the coronal plane traversing the Str. Parcellation is started in the coronal plane, when it first becomes visible in the Str and starts to separate the caudate nucleus from the putamen. Parcellation is continued in the coronal plane moving in the posterior direction. Only white matter should be included, and at the level of the GP, the QSM contrast can be used to distinguish between the GP and the ic.

The dorsolateral border of the ic is created by drawing a line between the dorsolateral tip of the caudate nucleus and the dorsomedial tip of the putamen, and following the contour of the Str. At rostral levels the contour of the caudate nucleus and the putamen is also used to define the medial border together with the radiation of the corpus callosum. At more caudal levels, a line is drawn following the contours of the Tha and the putamen to create the medial border.

**Fig. 16:**
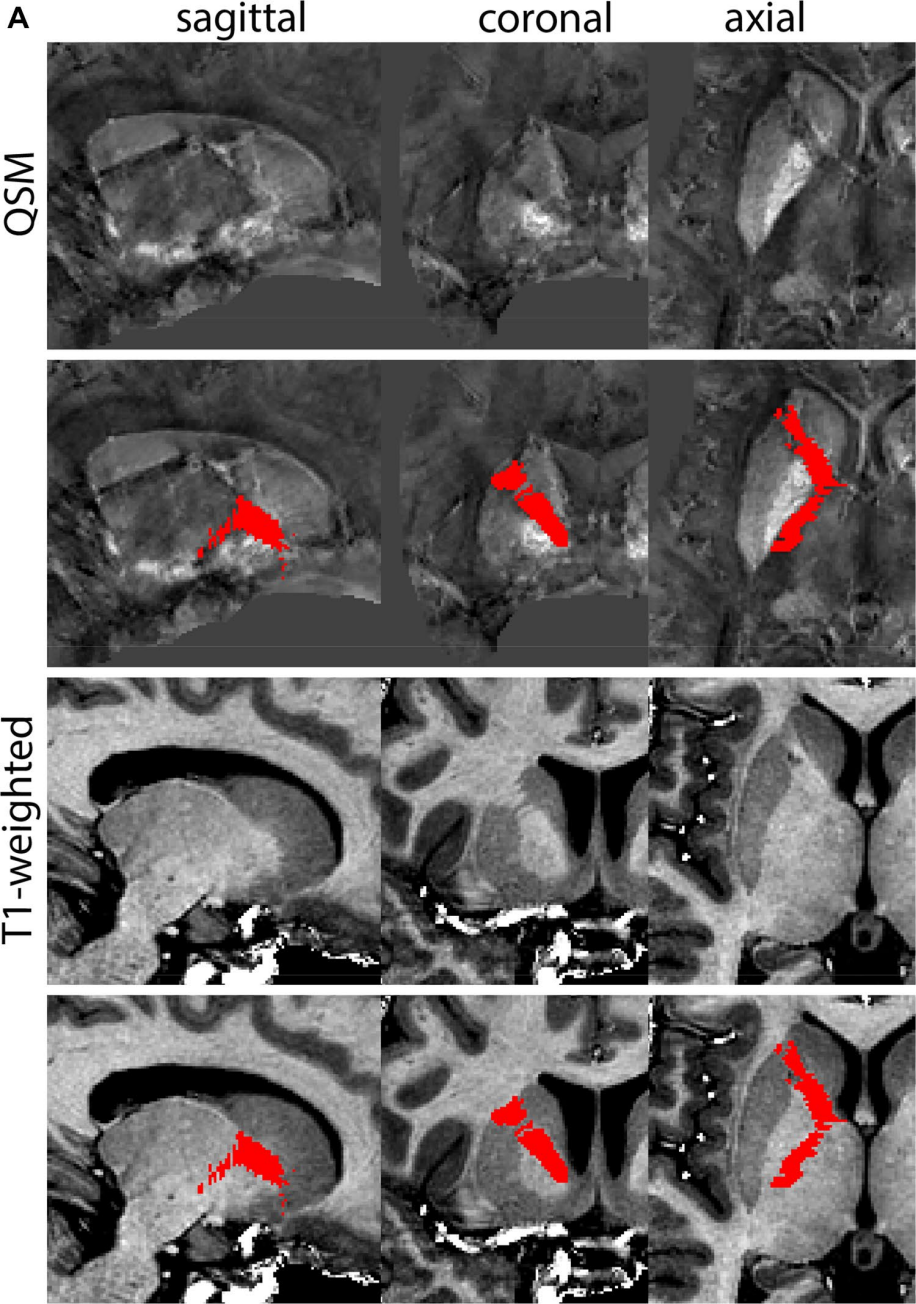

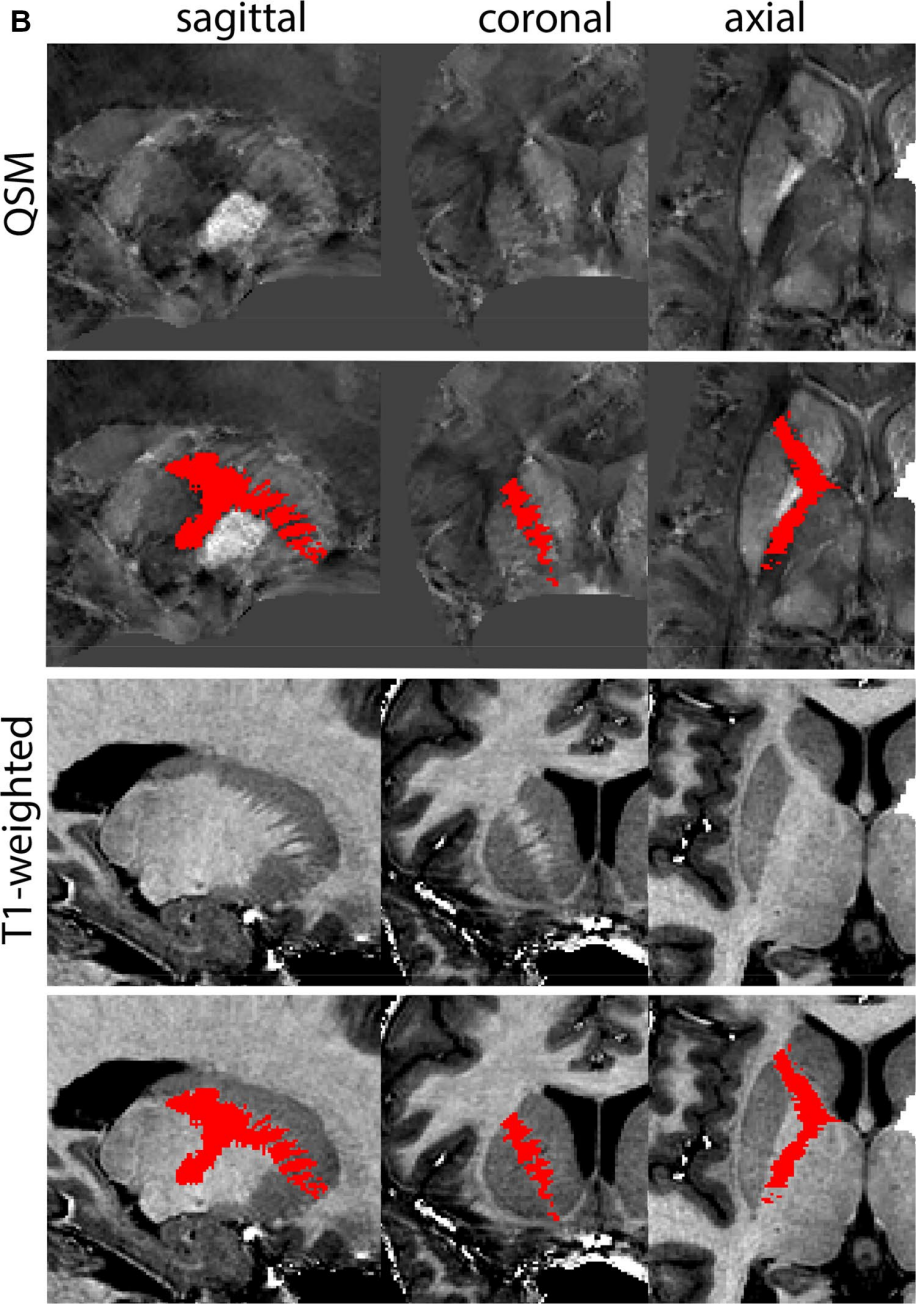

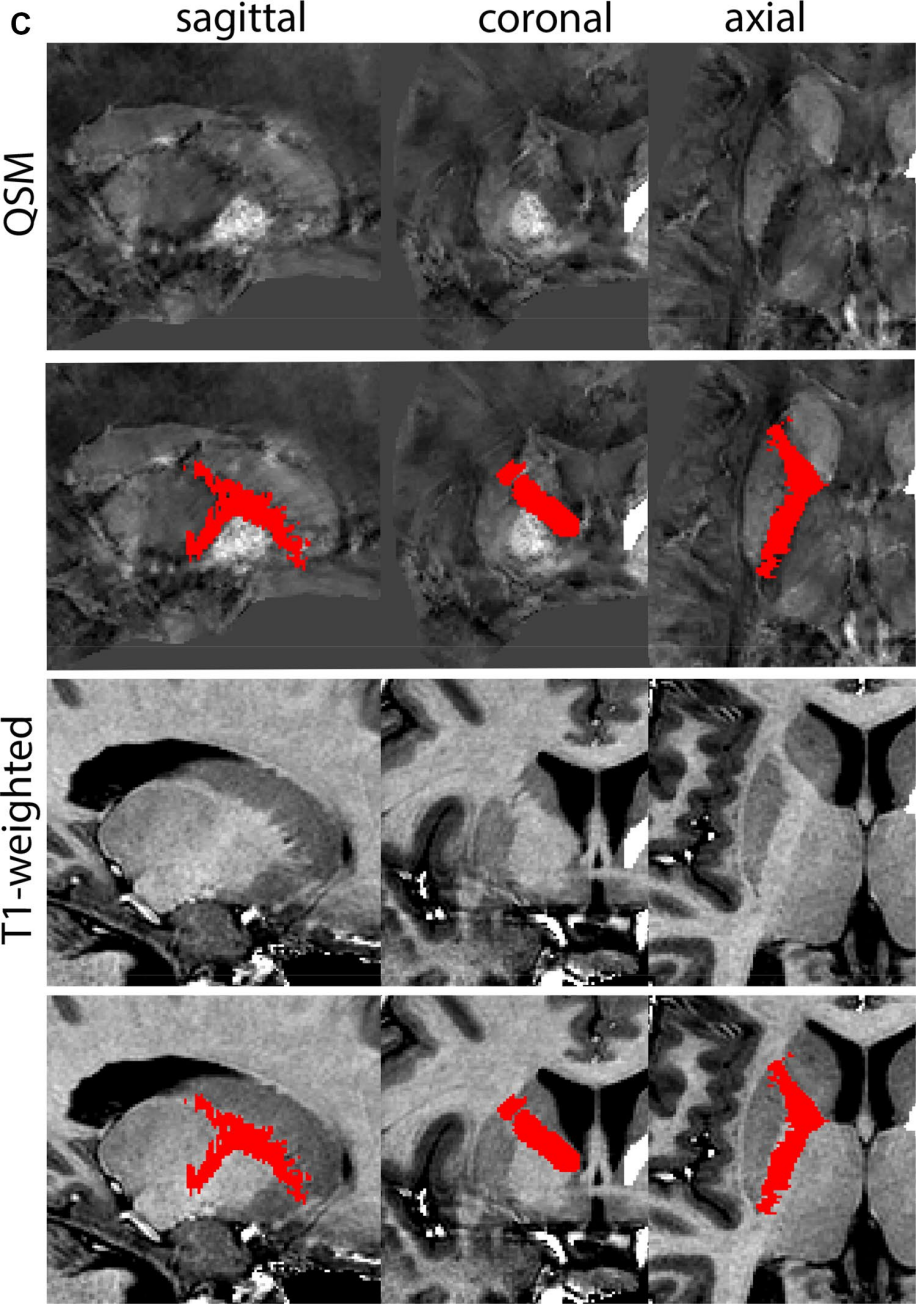

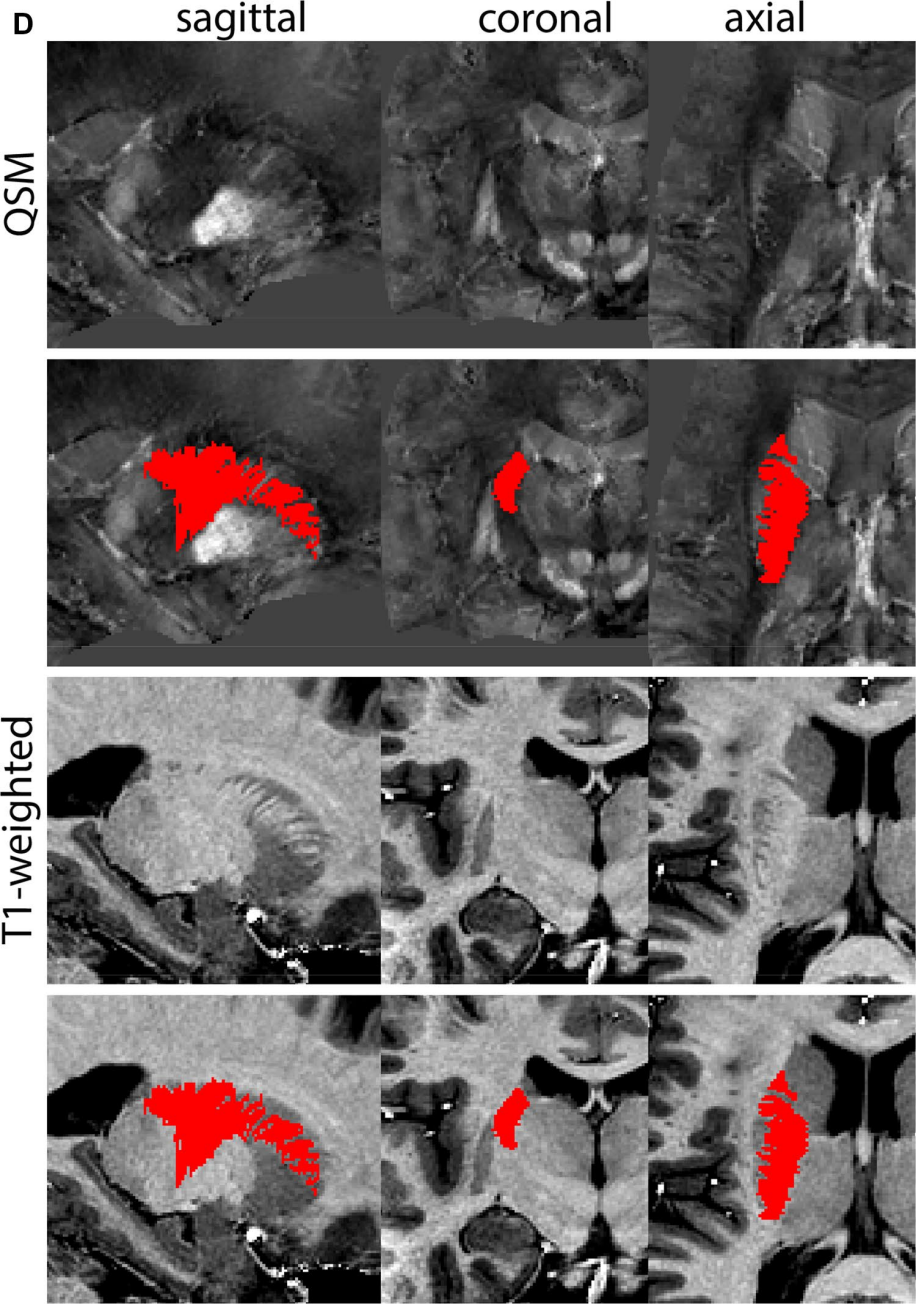
**a** The internal capsule (ic) is a white matter structure that separates the caudate nucleus from the dorsal striatum, carrying from and to cerebral cortex. Note the limited contrast in the T1-weighted scans between ic and the Globus Pallidus, and the additional contrast provided by the QSM contrast. **b** The internal capsule (ic) is a white matter structure that separates the caudate nucleus from the dorsal striatum. Note the clear appearance of striatal islands that lead to a jagged appearance of the ic. **c** The internal capsule (ic) is a white matter structure that separates the caudate nucleus from the dorsal striatum, carrying from and to cerebral cortex. **d** The internal capsule (ic) is a white matter structure that separates the caudate nucleus from the dorsal striatum, carrying from and to cerebral cortex

It is important to note that the anterior thalamic radiation and the eml of the Tha cannot be readily distinguished and are included in the parcellations. Striatal islands are often small, causing partial voluming effects that could potentially affect the delineation of the ic, and are likely to be influenced by interindividual differences in movement artifacts. Figure 16 shows illustrations of the ic parcellations at various anatomical levels.

### Inferior and superior colliculus

**Fig. 17:**
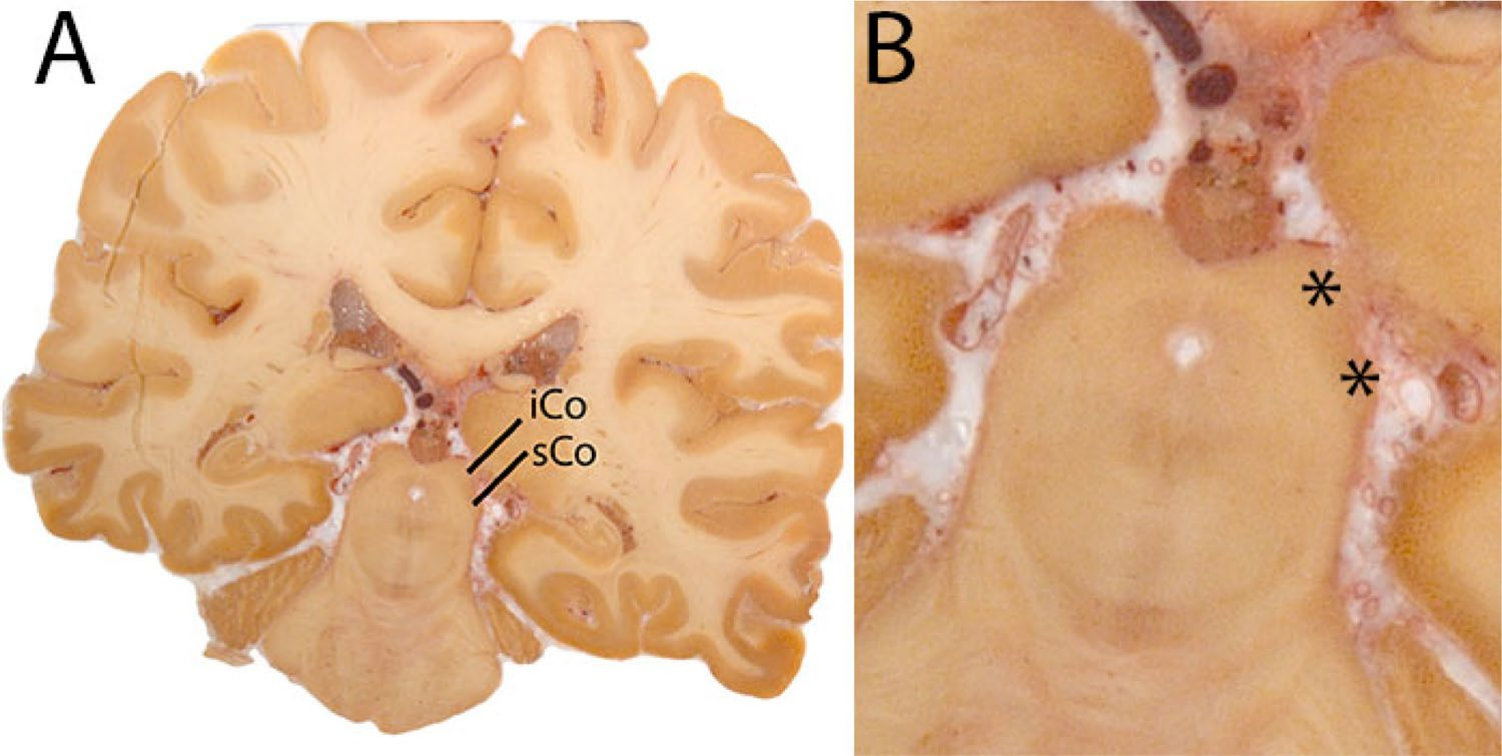
**A** The superior and inferior colliculus (sCO and iCO) in the midbrain and border the CSF which facilitates delineations. **B** Magnification, asterisks indicate the structrures of interest

The inferior colliculus (iCo) is an oval-shaped grey matter mound located in the caudal half of the mesencephalic tectum. The superior colliculus (sCo) is a gray matter mound located in the rostral half of the mesencephalic tectum (Fig. 17).

#### Parcellation

The iCo and sCo are delineated on the R2* map or T2*-weighted image. In the R2* map for the sCo and iCo are hyperintense. In T2* weighted images contrasts are inverted. The iCo is located near the tectum of the midbrain, caudal to the sCo on the dorsal aspect of the mesencephalon, lateral—in its dorsal aspect- to the periaqueductal gray (PAG), and ventrally to the cuneiform nucleus. The iCo is laterally bordered by the ventricular system. The sCo is visible in the midbrain, rostral to the iCo, lateral to the PAG and bounded laterally by the cerebrospinal fluid (CSF). Both structures appear on T_2_* maps as a hypointense structure and a hyperintense structure on R2* maps. Both structures are visible on T2* maps; however, the CSF contrast is better on the R2* maps.

**Fig. 18:**
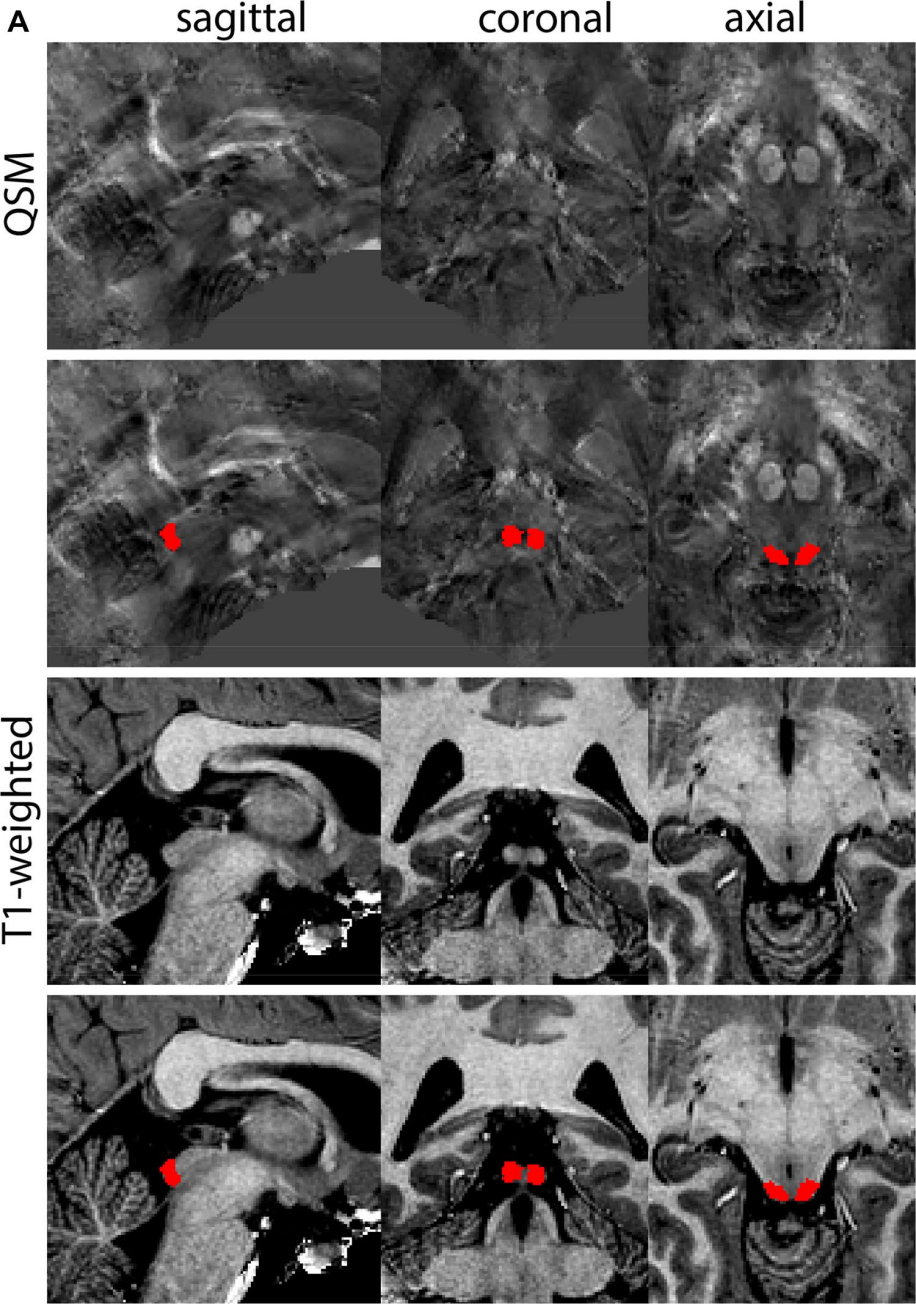

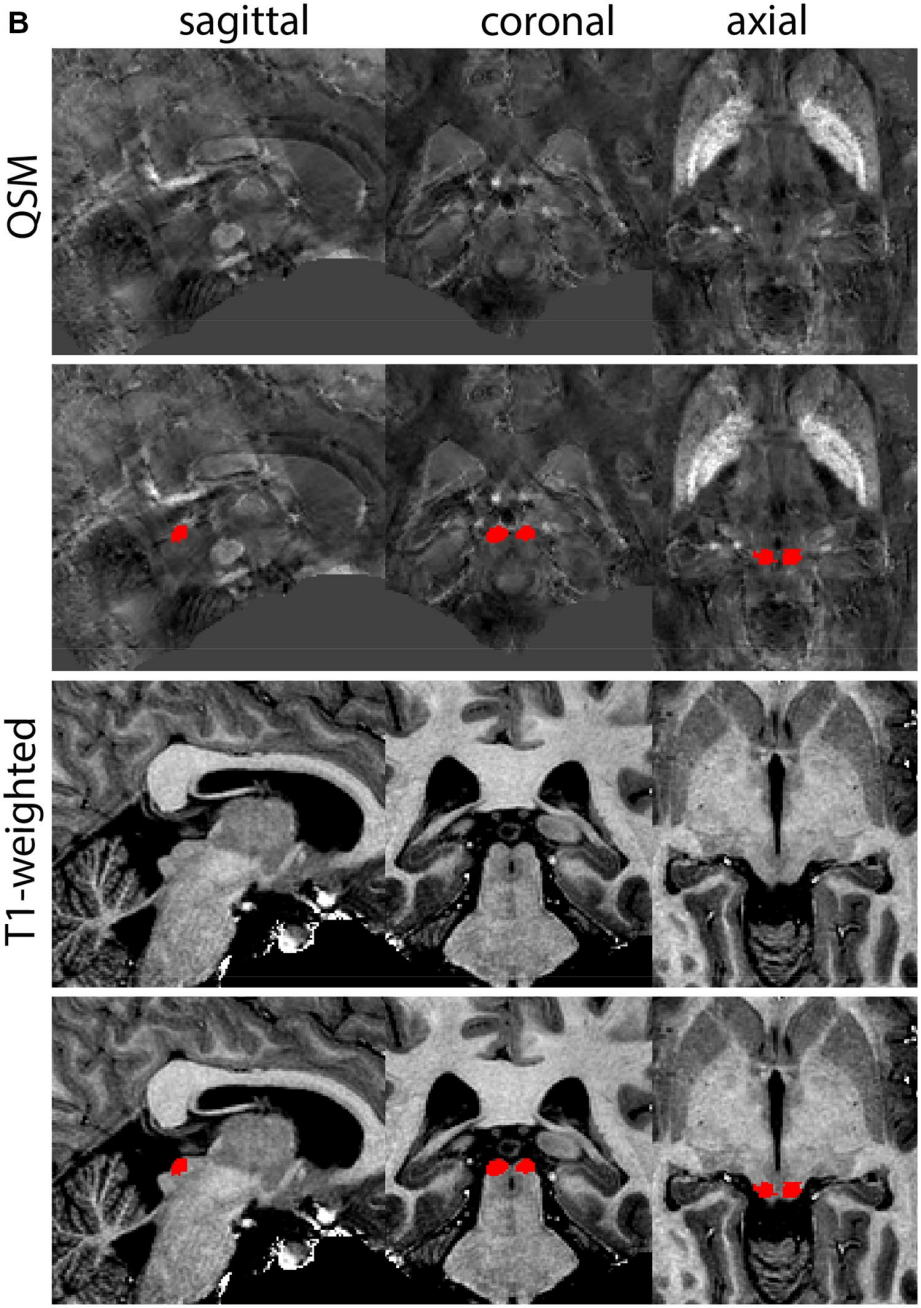
**a** The inferior colliculus can easily be located due to the characteristic location at the mesencephalic tectum. **b** The superior colliculus can easily be located due to the characteristic location at the mesencephalic tectum above the inferior colliculus (sagittal view)

To start, the center of the RN is identified, and the cursor is placed there. At this point, the sCo and iCo will appear on the sagittal image at the roof of the mesencephalic tectum. The structures are small, and well defined; therefore, no additional information on arbitrary decisions is provided. The delineation is driven by shape consistency. The cursor is then moved to the center of the sCo in the sagittal image, subsequently performing the delineation in the axial plane. First move in a dorsal direction, complete the delineation in the ventral direction. Shape consistency should be checked in the coronal as well as the sagittal plane. The iCo is delineated in the same manner. In Fig. 18 delineations of the sCo and iCo are illustrated at various anatomical levels.

### The habenular complex

**Fig. 19:**
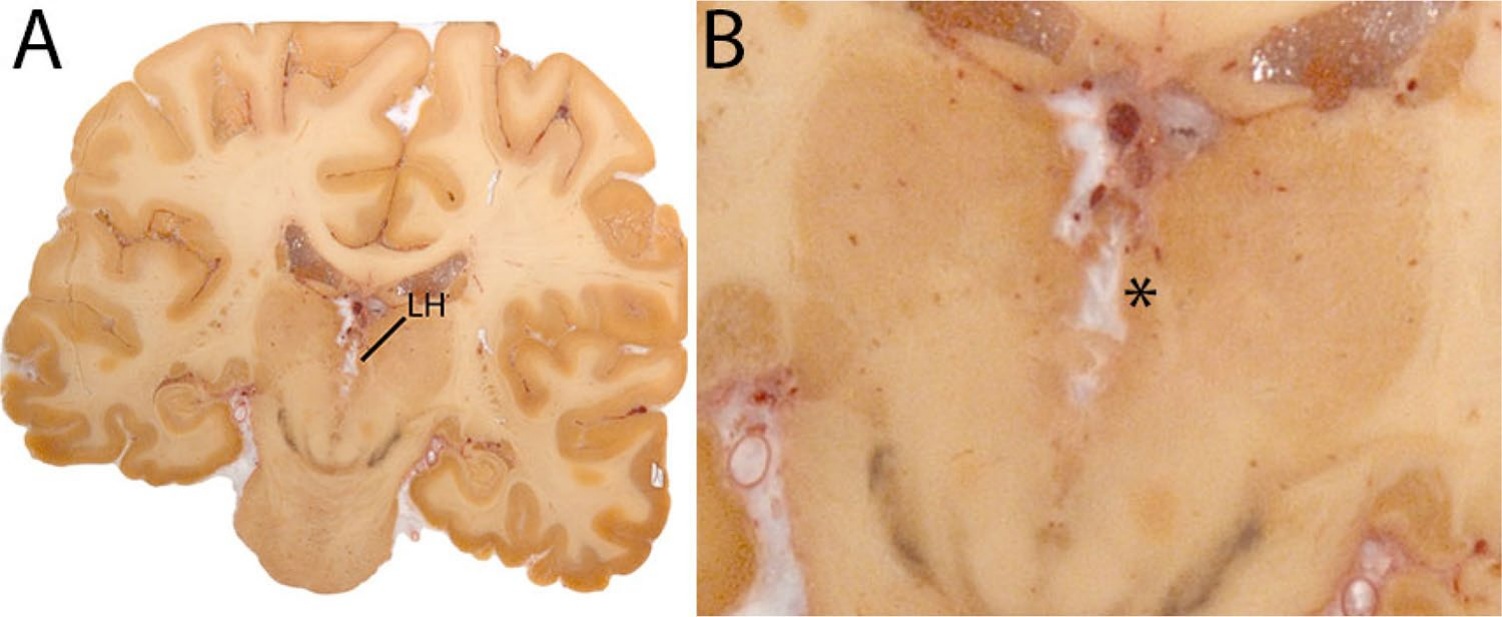
**A** The Habenula (LH) is a small structure medial from the Tha, and comprises the Medial and Lateral Habenula. **B** Magnificaiton, note the protrusion into the 3V

The Habenular complex is a small anatomical structure located adjacent to the 3V, medial to the Tha. The region of interest parcellated includes both the lateral and medial habenula since the spatial resolution available in in vivo scans is insufficient to distinguish between them (Fig. 19).

#### Parcellation

T1-weighted images are used and allow to exploit the relatively high amount of white matter plexuses contained in the habenula and increase its contrast with surrounding structures. The parcellations are started in the middle of the habenular complex, first performing the parcellations in the anterior direction, followed by parcellation of the caudal extent of the habenula. After the coronal parcellation, the results are confirmed using the axial plane. Partial voluming effects are usually observed medially, where the habenular complex borders the 3V. These voxels are included in the parcellations.

The rostral border of the parcellation is set where hyperintense voxels belonging to the tissue of the stria medullaris (sm) are first seen bulging into the 3V as the 3V begins to narrow. The sm is a white matter structure located dorsolaterally and connecting to the habenula. The ventromedial border is not always discernable, as both the sm and habenula appear hyperintense on T1-weigthed contrasts. Therefore, a small part of the sm will most likely be included in the parcellation. The most rostral slices included will contain some sm. The voxels containing white matter of are considered part of the habenula if: They are located at the medial tip of the sm, and they bulge into the 3V where it narrows and immediately borders the 3V. Voxels bordering the ventricles that are located further dorsolateral are considered to belong to the sm.

**Fig. 20:**
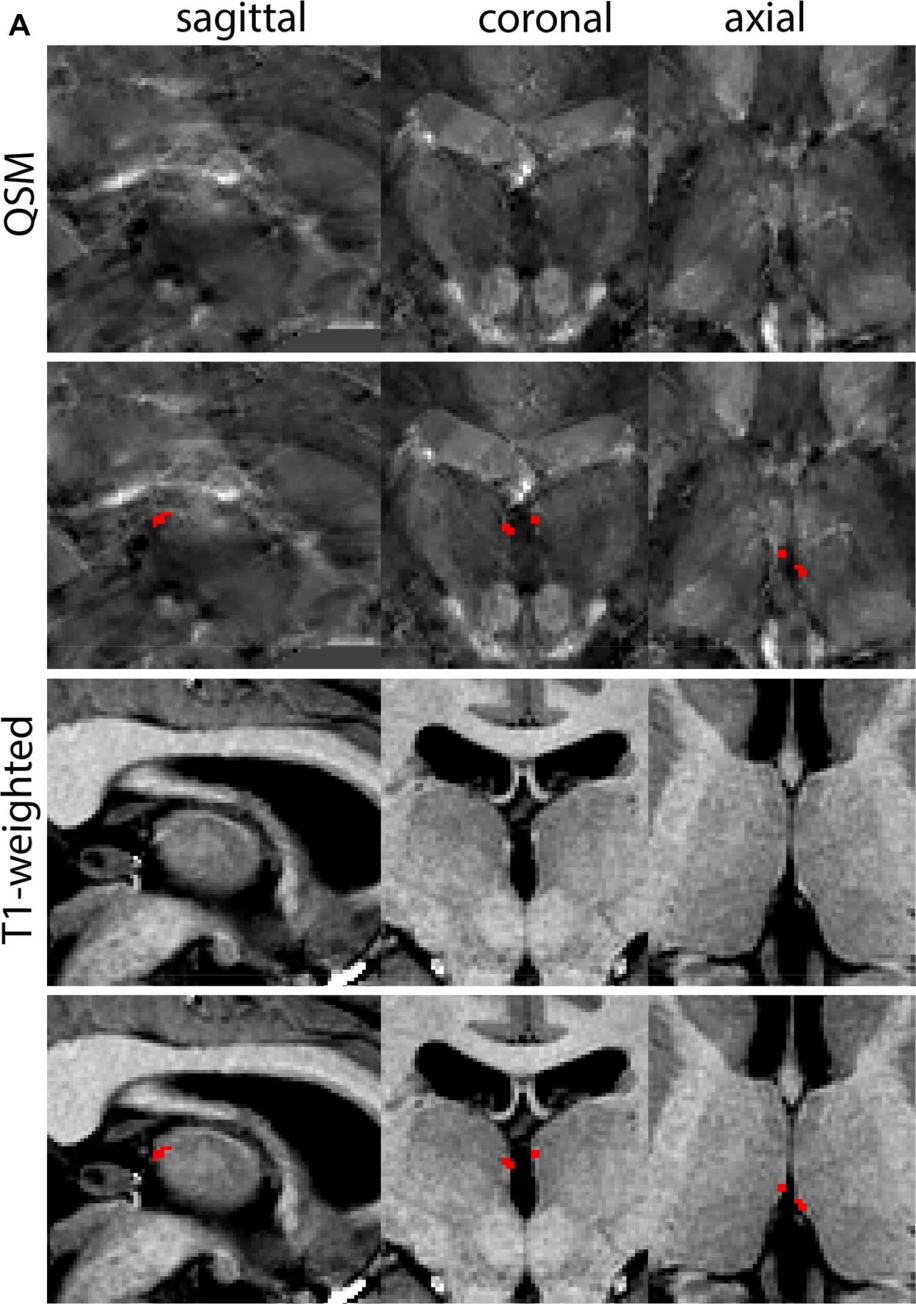

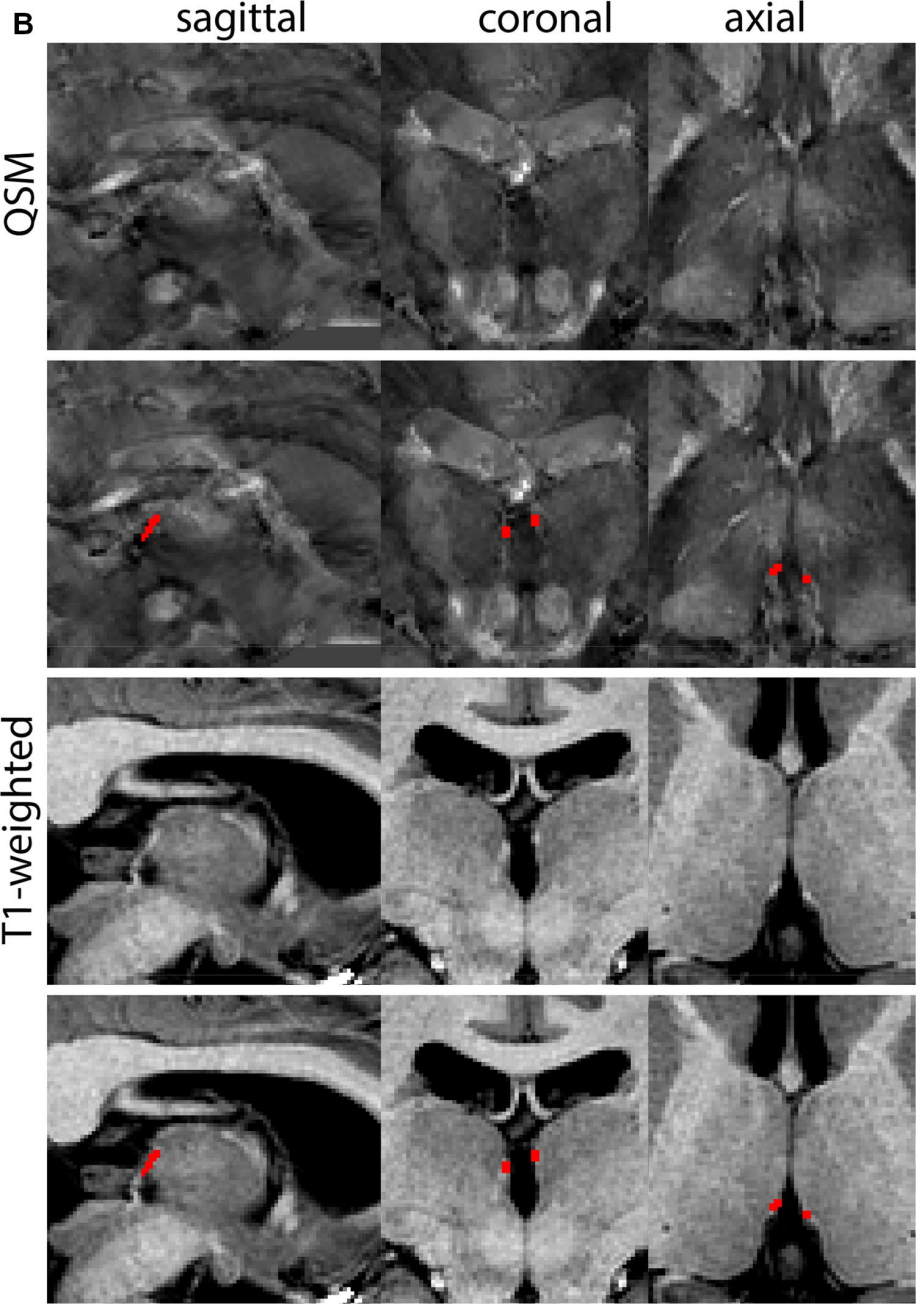
**a** The habenular complex is visible as a hyperintense structure on T1-weigthed images. Note the modest size of the structure. **b** The habenular complex is visible as a hyperintense structure on T1-weigthed images. Note the slightly elongated shape in the sagittal view

The parcellation results should appear as a single continuous structure. A gap at the rostral extent, where parcellated hyperintense voxels bulging into the 3V disappear in a few of the more posterior slices before appearing again, indicates that parcellations have started too rostral. The borders of the habenula include the CSF of the 3V medially and Tha laterally. The ventral border includes either the posterior commissure or habenular commissure (depending on visibility) at caudal levels, or the Tha at more rostral levels. In central slices, the habenula can appear as a triangular structure, whereas in others it will appear elongated and have continuous borders with the sm and fasciculus retroflexus (fr). The sm and fr are continuous with the habenula and both contain high levels of white matter plexuses making it is difficult to separate these structures based on T1-weighted image contrasts and anatomical landmarks; therefore, they are included in the mask if they are continuous in the coronal view. The transverse view can assist in the separation of the habenula from the fr. The sm and fr are only included within the segmentation when they appear continuous with the habenula within the coronal view and cannot be separated. In Fig. 20, delineation results of the Habenular complex are illustrated.

### Periaqueductal gray

**Fig. 21:**
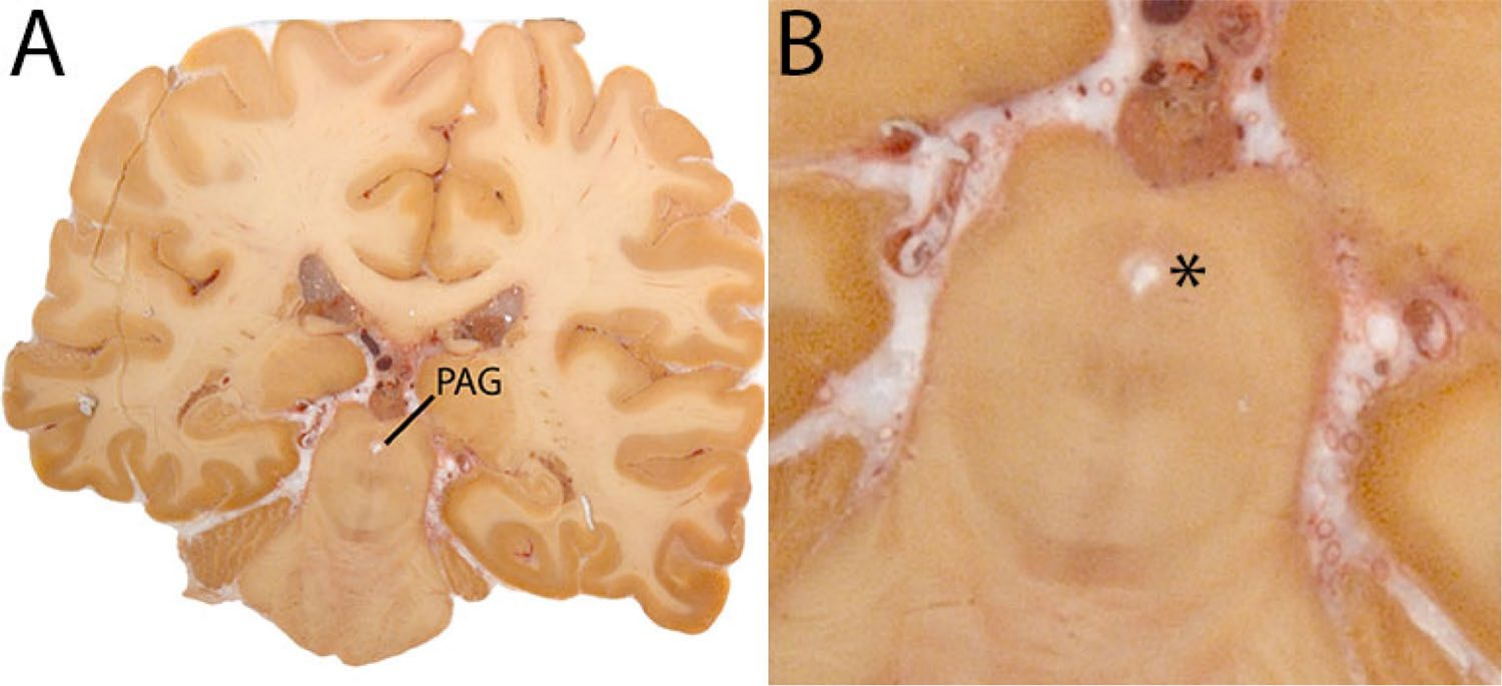
**A** The Periaqueductal and periventricular grey (PAG) form a grey matter column which lines the midbrain aqueduct. **B** Magnification, asterisk indicates the PAG

The PAG appears as a column of gray matter surrounding the aqueduct of Silvius (midbrain aqueduct). The PAG is a dense cell region. The rostral border of the PAG is determined to be at the level of the superior border of the midbrain. The inferior border is marked at the start of the superior medullary velum. The border with the Raphe nuclei cannot be discerned on available MRI contrasts, which is in line with descriptions of the neurons of the Dorsal Raphe nucleus extending into the PAG. As a result, delineations include both PAG and part of the Raphe nuclei. Additionally, the periventricular gray (PVG) extending to the level of the fourth ventricle (4V) is included (Fig. 21).

#### Parcellation

**Fig. 22:**
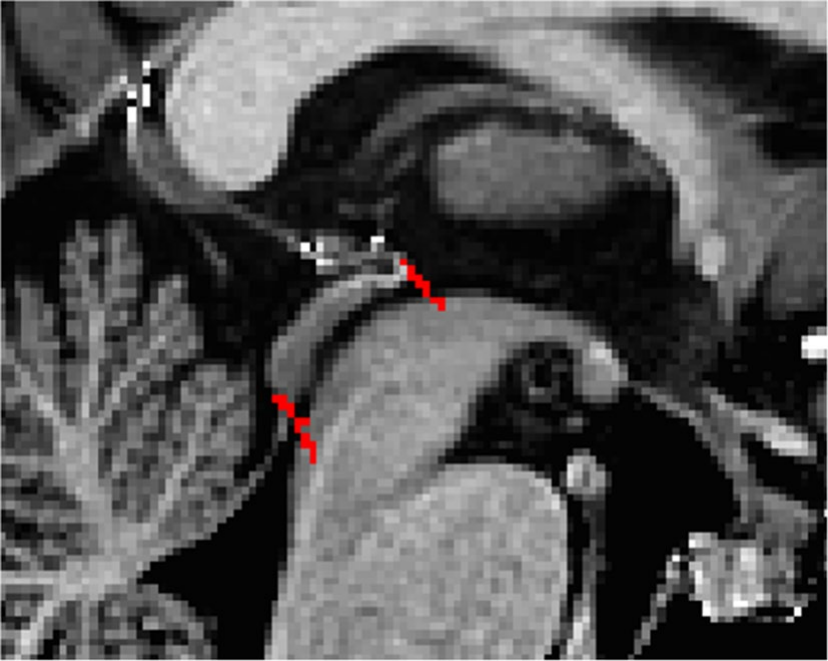
Starting point for the parcellations. Red marks indicate the upper and lower border of the midbrain aqueduct in the sagittal plane

T1-weighted contrasts are used for parcellations. At midsagittal levels the midbrain aqueduct is visible. The Superior medullary velum is identified and used for anatomical reference. The borders of the aqueduct are indicated for anatomical reference (Fig. 22).

**Fig. 23:**
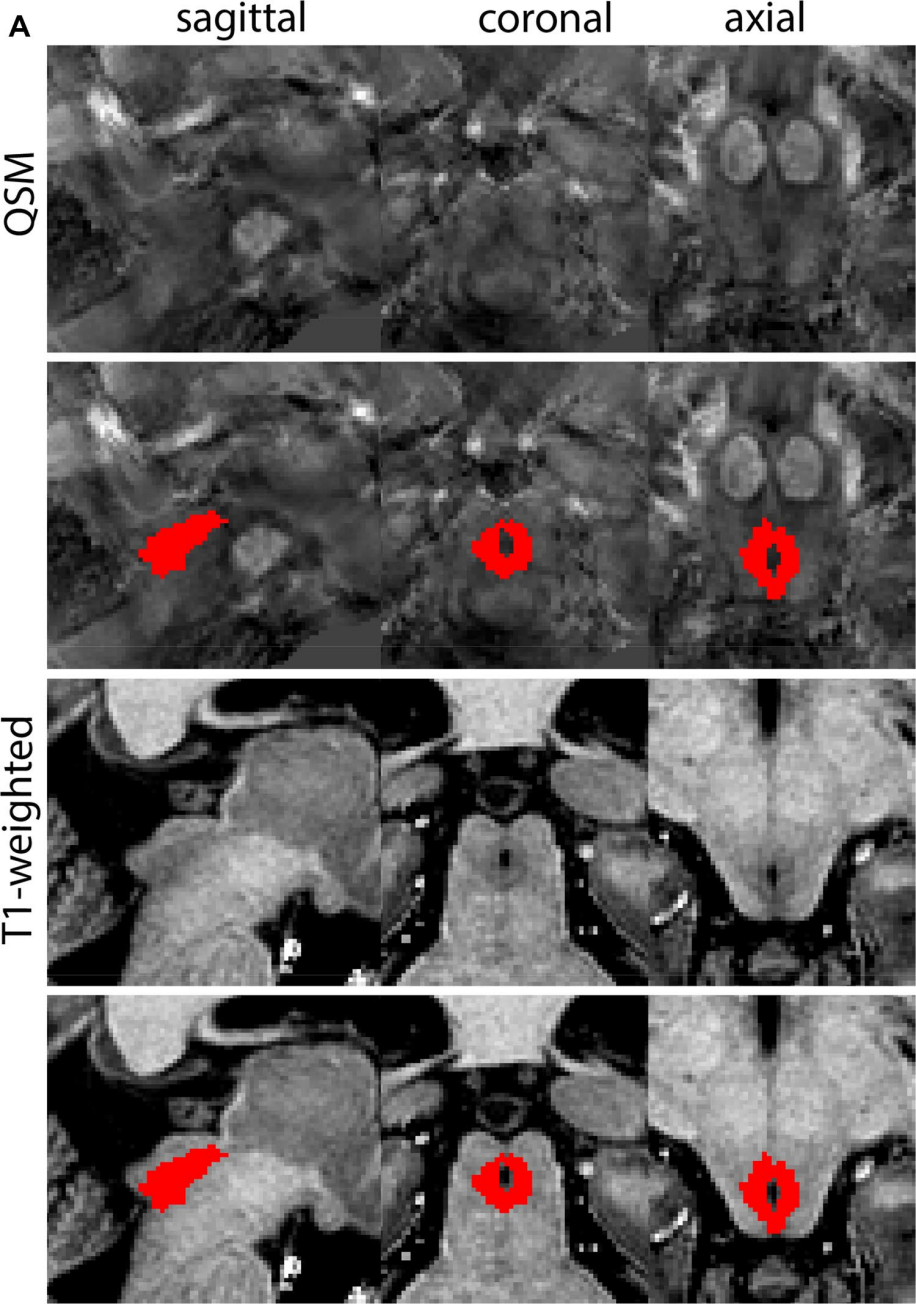

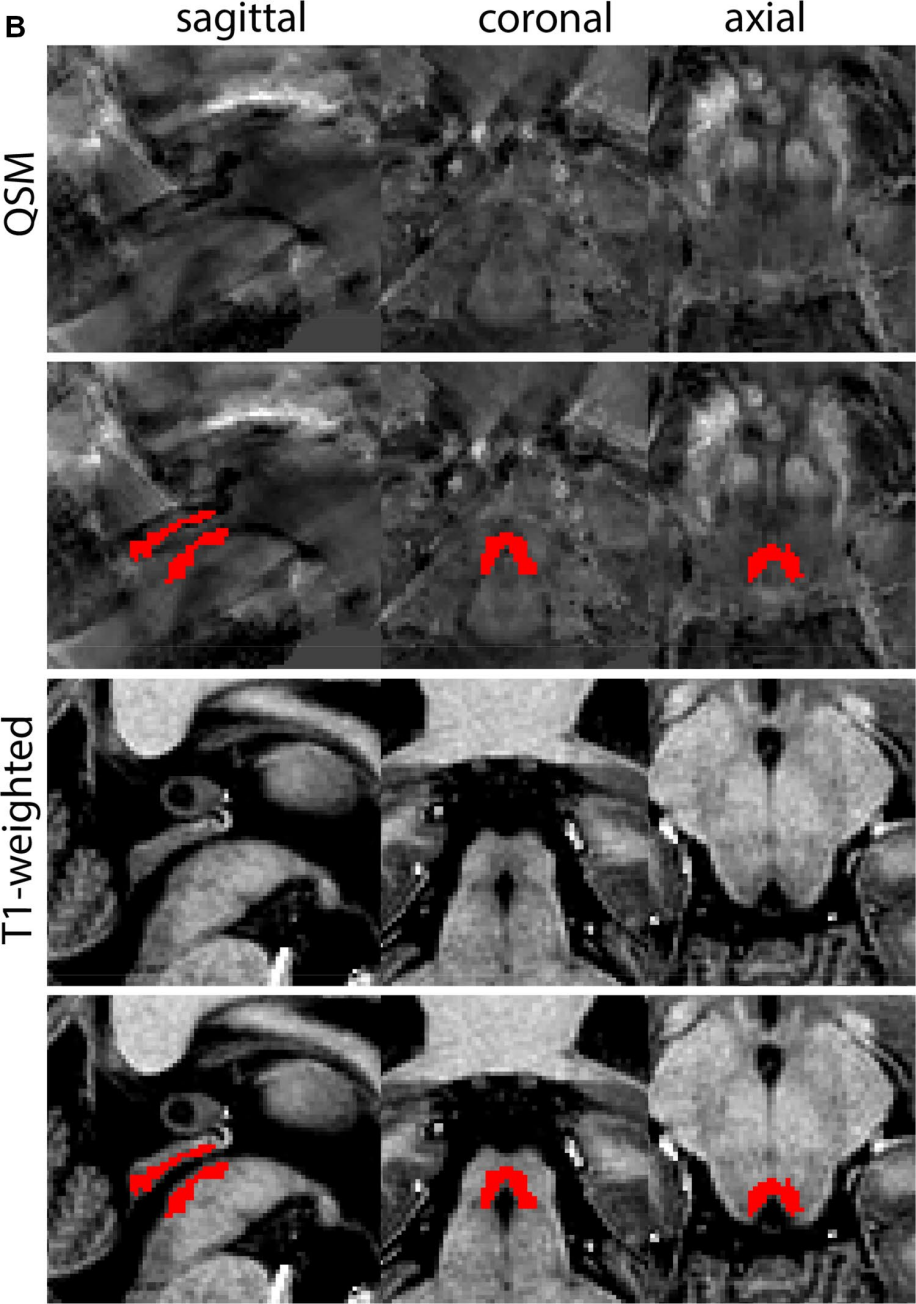

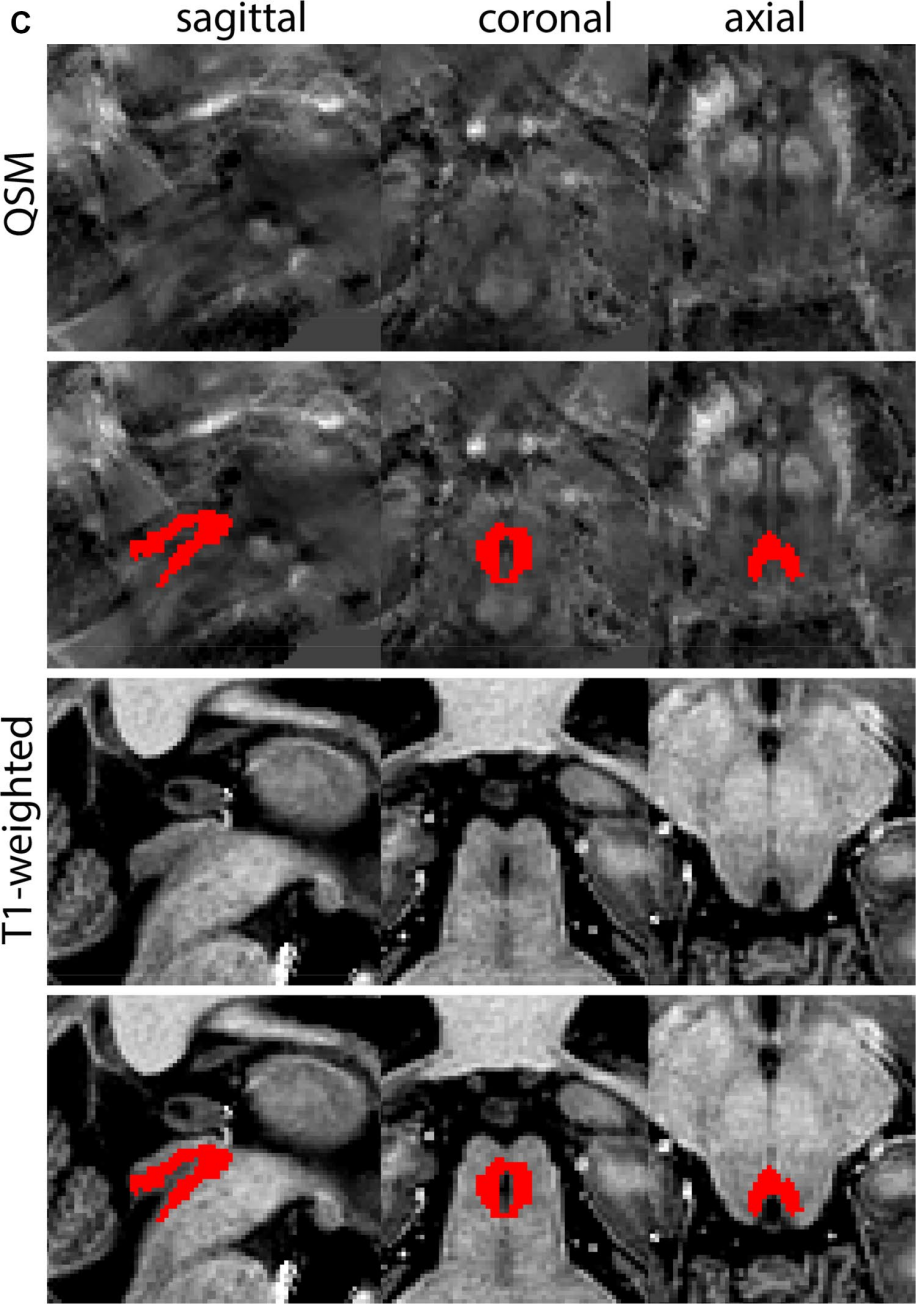
**a** The periaqueductal and periventricular grey surround the midbrain aqueduct. Note the visibility of the borders on the T1-weigthed images on which it appears as a hypointense structure. **b** The periaqueductal and periventricular grey surround the midbrain aqueduct. Note the apparent division in two structures in the sagittal plane, which results from the cross-cut of the structure. **c** The periaqueductal and periventricular grey surround the midbrain aqueduct. Note the partial division in the sagittal plane, which results from the cross-cut of the structure

Parcellations are then started at a central level in the PAG in the axial plane. Delineations are first performed slice by slice moving in superior direction, separating the left from the right hemisphere guided by the aqueduct and the symmetrical appearance of the structure. Delineations are completed when the aqueduct dissolves in the 3V. The sagittal and coronal view are used to check for consistency. Delineation is continued from the starting point down to include the PVG. Parcellations are illustrated at various anatomical levels in Fig. 23.

### Pedunculopontine nucleus

**Fig. 24:**
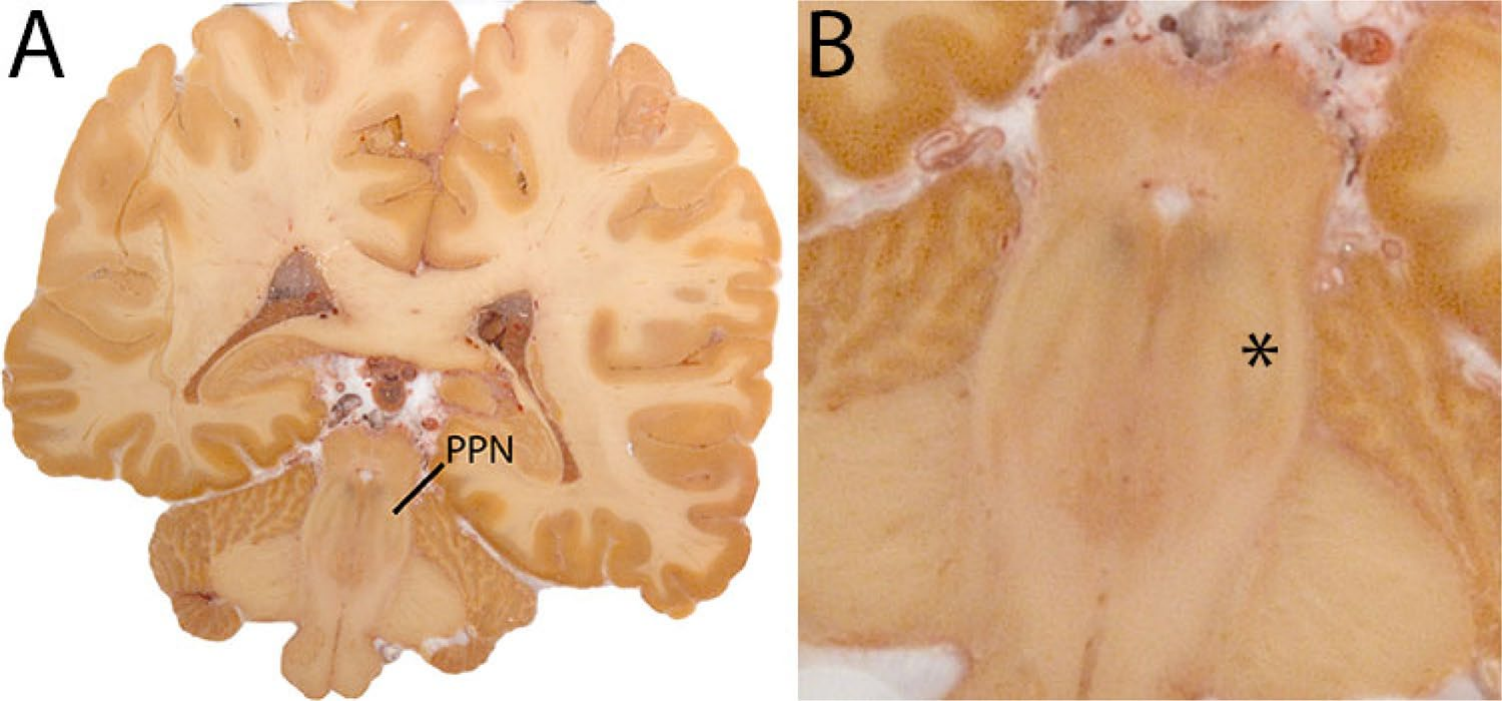
**A** The Pedunculopontine nucleus indicates at a central level. **B** Magnification, asterisk indicates the PPN

The Pedunculopontine nucleus (PPN) is located in the pons at the level of the decussation of the superior cerebellar peduncles near the inferior colliculus, the trochlear nucleus and the intercollicular area. Laterally, the PPN is bounded by fibers of the medial lemniscus, the spinothalamic tract, and the lateral lemniscus. The medioventral border of the rostral PPN is adjacent to the rubrobulbar/spinal tract and the dorsomedial aspect of the SN. The mediodorsal aspect of the PPN is bordered by the medial longitudinal fasciculus and the trochlear nucleus. Dorsally, the PPN contacts the cuneiform nucleus and is close to the mesencephalic nucleus of the trigeminal nerve, the midbrain aqueduct, and PAG. The most caudal pole of the PPN is adjacent to the locus coeruleus. The PPN is bordered medially by the decussation of the superior cerebellar peduncle and the central tegmental tract (Fig. 24).

#### Parcellation

**Fig. 25:**
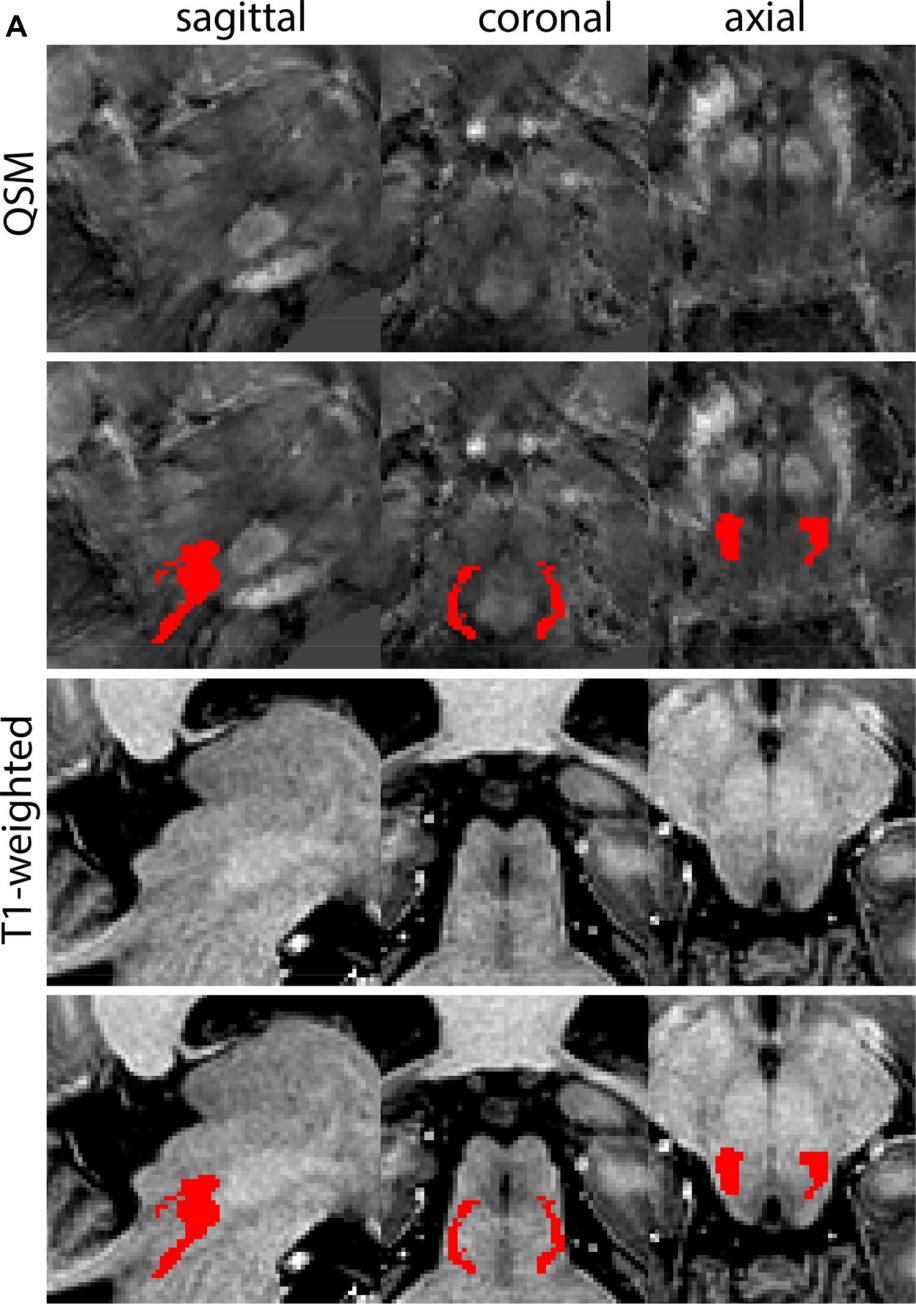

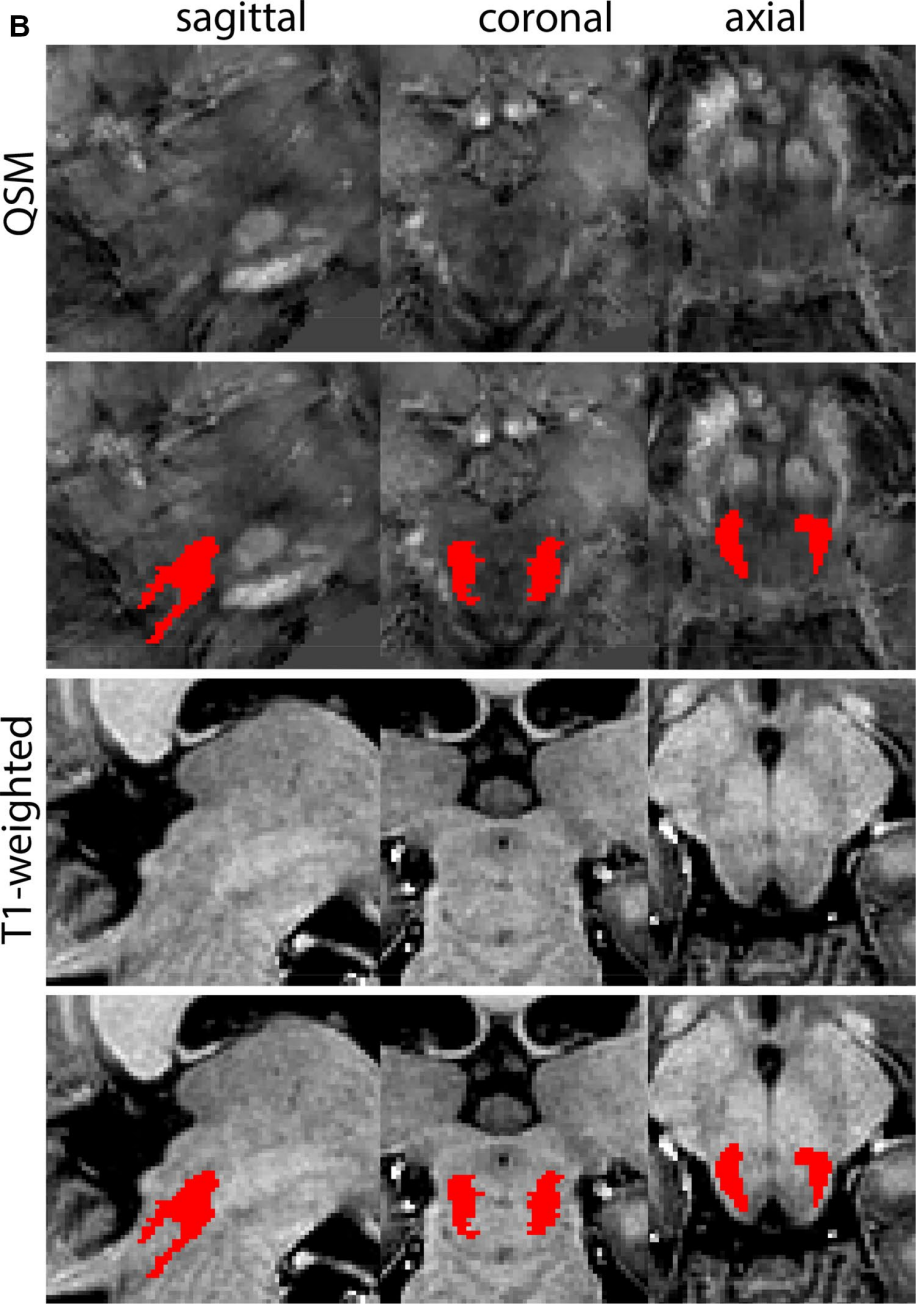

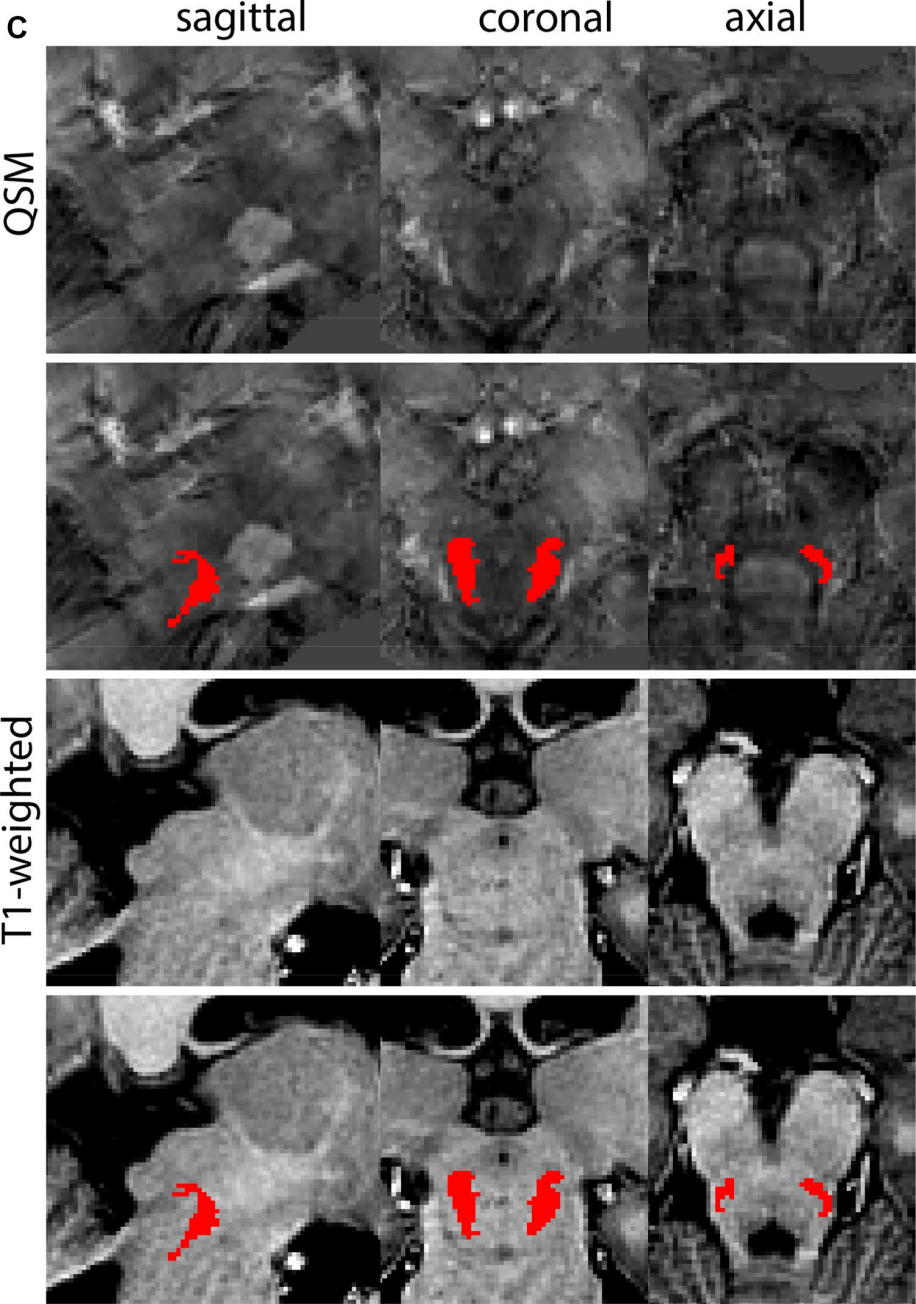
**a** The pedunculopontine nucleus (PPN) is a grey matter structure located in a white matter rich area. As a result it presents as a relatively hypointense structure on T1-weighted images. **b** The pedunculopontine nucleus (PPN) is a grey matter structure located in a white matter rich area. As a result it presents as a relatively hypointense structure on T1-weighted images. Note the appearance of the PPN in the sagittal plane at this level. **c** The pedunculopontine nucleus is a grey matter structure located in a white matter rich area. As a result it presents as a relatively hypointense structure on T1-weighted images. Note the difference in the sagittal appearance with (**b**)

Parcellations are performed on the T1 map. The PPN is bordered by WM tracts. The surroundings of the PPN will be hypointense, whereas the PPN itself will be relatively hyperintense. The parcellation is started at the metencephalic-pontine area. The PPN is sickle shaped in the middle of the nucleus on the axial view. Delineations are first performed in the axial plane moving in the superior from there. After completion in this direction, parcellations are continued in caudal direction until completed. Parcellations are illustrated at various anatomical levels in Fig. 25.

### Subcallosal cingulate gyrus

**Fig. 26:**
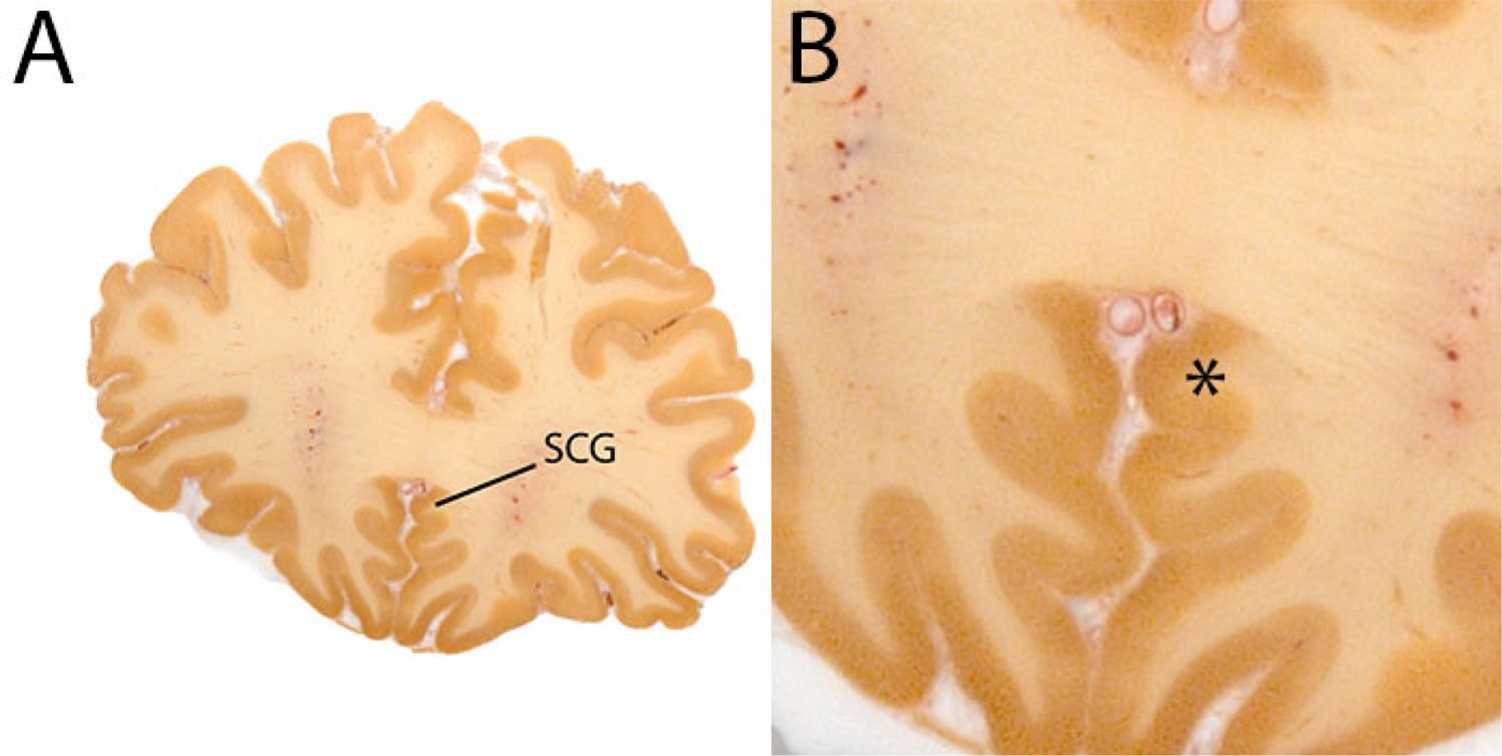
**A** The subcallosal cingulate gyrus (SCG) is located under the corpus callosum. **B** Magnification, asterisk indicates the SCG

The subcallosal cingulate gyrus (SCG) is part of the cingulum and includes Brodmann area 25 and parts of 24 and 32 (Hamani et al. 2011). As the name indicates it is located ventral to the corpus callosum. This structure is of particular interest in view of its potential role as a DBS target in the treatment of Major Depression. In DBS the WM of the subcallosal ACC is target. The protocol by McCormick et al. (2006) was used as the basis for the description below (Fig. 26).

#### Parcellation

Move to the midsagittal position on the T1map. Identify the most rostral tip of the corpus callosum (CC). The rostral border of the SCG is defined in the coronal plane. The parcellations do not extend beyond the anterior commissure, and voxels in coronal slices anterior to the genu of the CC should not be included.

The location of the start of the putamen is used to determine the location of the posterior boundary of the SCG in the coronal plane. This border is located at the level that the putamen can first be discerned. This border can appear in different coronal slices for the left and right hemisphere.

Locate the SCG under the CC. Note that in a subset of participants, a secondary, smaller gyrus can be present, whereas others will only have a single gyrus. Use the continuity with the rostral ACC as a reference if needed. Delineate the WM in the subcallosal ACC. The lateral WM borders are defined arbitrarily by drawing a line between the subcallosal cingulate gyrus and the subcallosal gyrus.

**Fig. 27:**
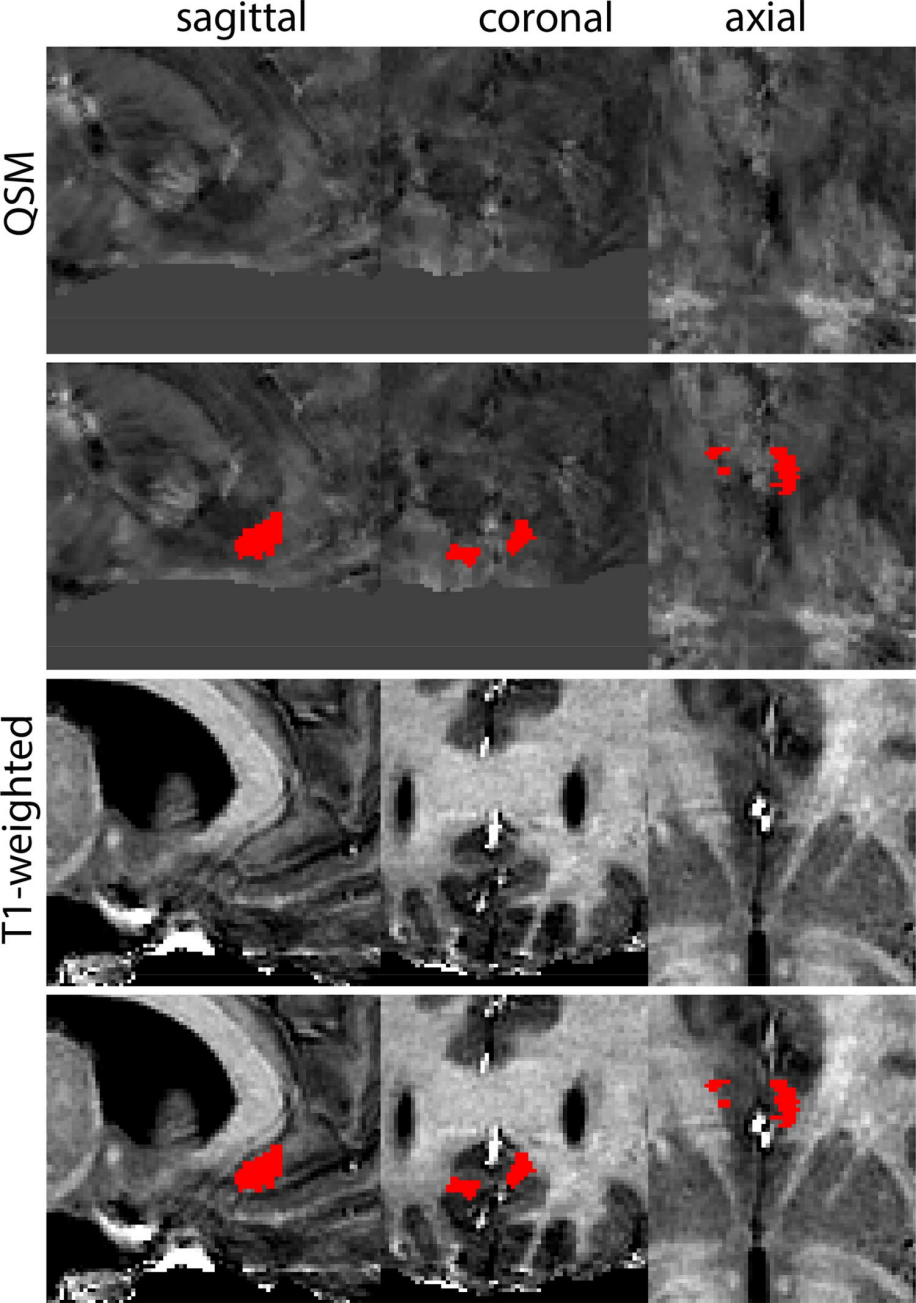
The Subcallosal Cingulate Gyrus (SCG) mask only contains an outline of the WM. Note the frontal border of the structure in the sagittal, which is defined by the corpus callosum

Continue parcellation in the coronal plane, first in the anterior and then posterior direction until the borders are reached. Check the different views to ensure consistency, and finish the parcellation. Parcellations are illustrated in Fig. 27.

### Striatum

**Fig. 28:**
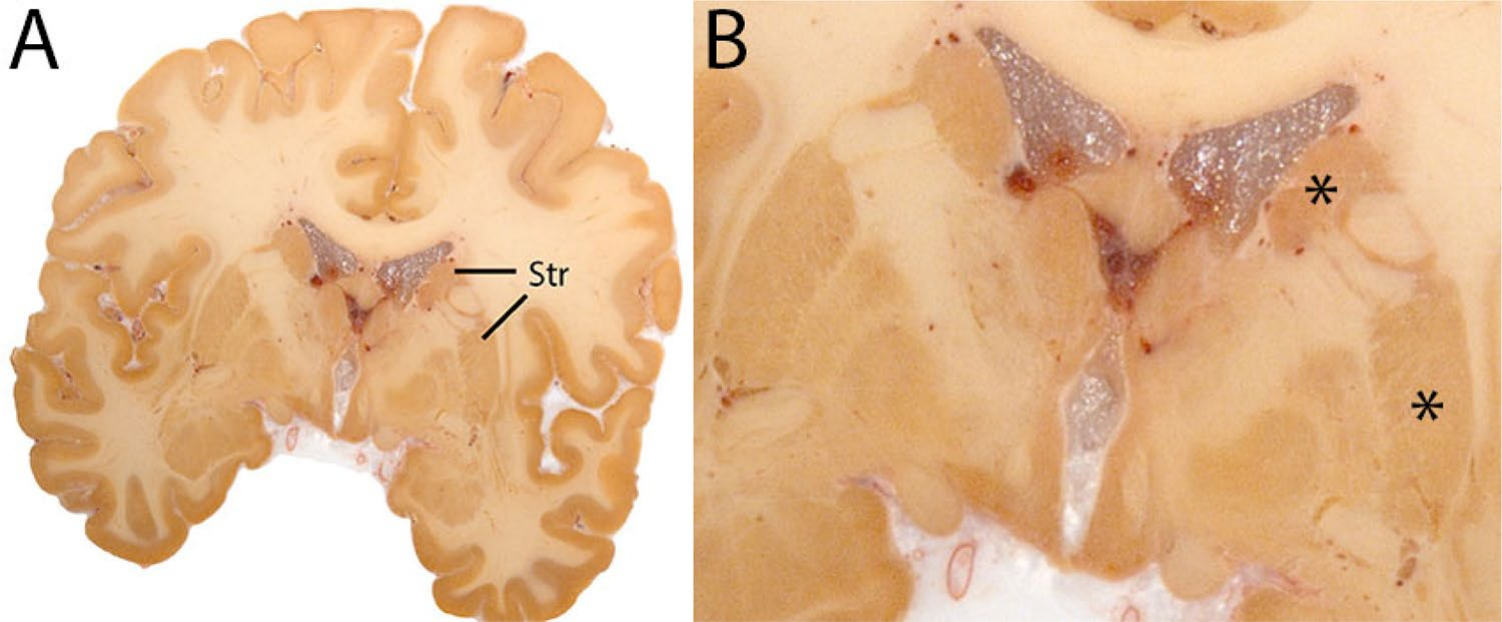
**A** The striatum (Str) includes the caudate (top), putamen (bottom) and Nucleus Accumbens (visible at more rostral levels). The borders between these structures cannot be visualized using MRI and the striatum is therefore parcellated as a single structure. **B** Magnification, asterisks indicate caudate and putamen

The Str is the largest grey matter structure in the basal ganglia. The caudate nucleus, the putamen and the nucleus accumbens, together form a continuous structure and are, therefore, parcellated together as the Str. The caudate nucleus is bordered medially by the lateral ventricles. The Str is found lateral to the GPe and the ic crosses through the Str separating the caudate and putamen in the coronal view (Fig. 28).

#### Parcellation

The most rostral part of the Str is identified by the appearance of the caudate nucleus adjacent to the lateral ventricle in the coronal plane on the T1-weigthed contrast. Medial borders are defined by the lateral ventricle. Lateral borders are clearly visible on both the T1-weighted and the QSM contrast. The caudate nucleus is delineated and can be traced in the coronal plane moving in posterior direction. In coronal sections, the *ic* will traverse the Str and separate the caudate nucleus from the putamen as the Str increases in size. Moving posterior, the GP will appear. The T1-weighted contrast can be used to identify the internal capsule, and the GP is clearly visible on the QSM contrast. Following the Str in caudal direction, the anterior commissure will become visible on the T1-weighted contrast.

**Fig. 29:**
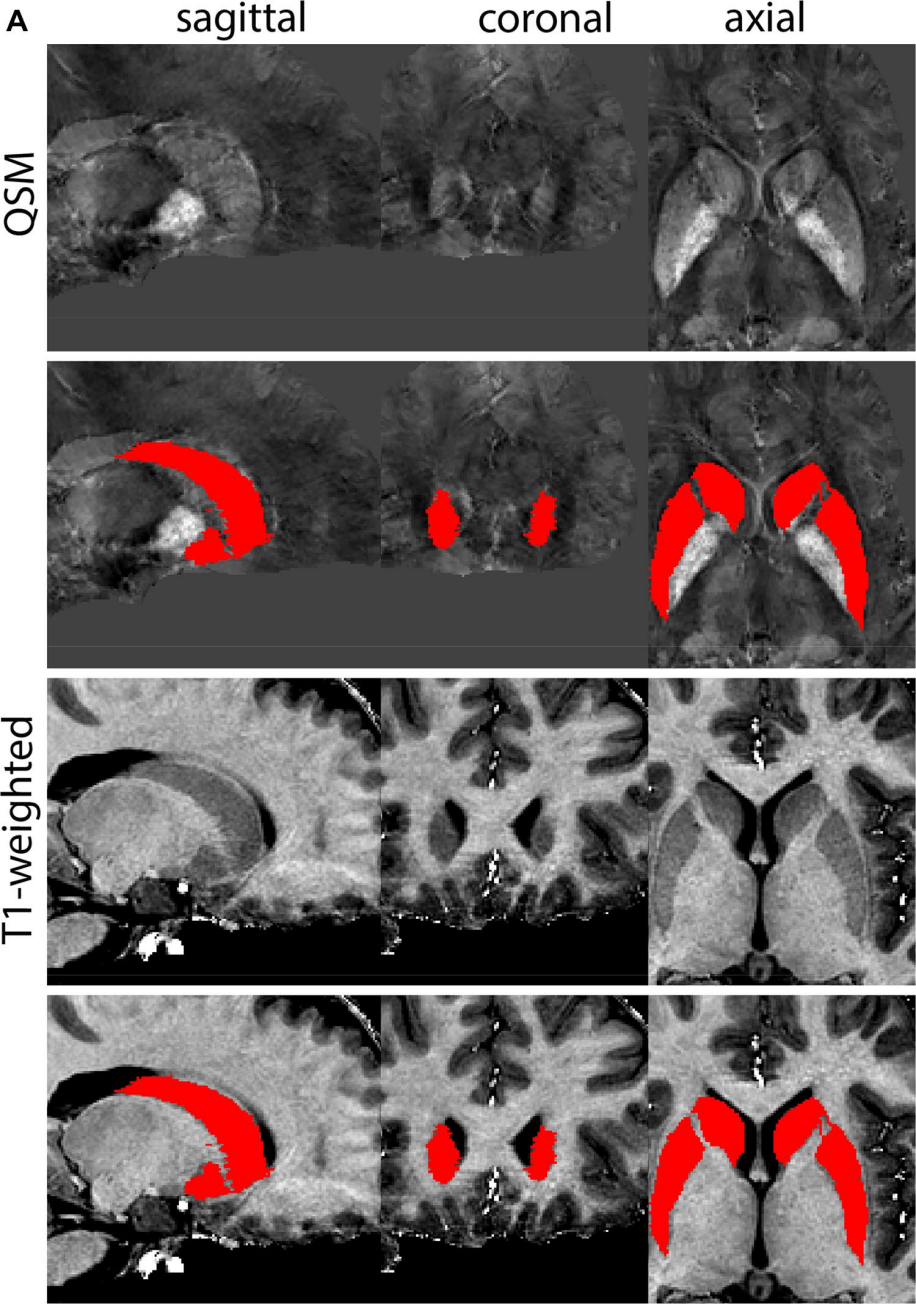

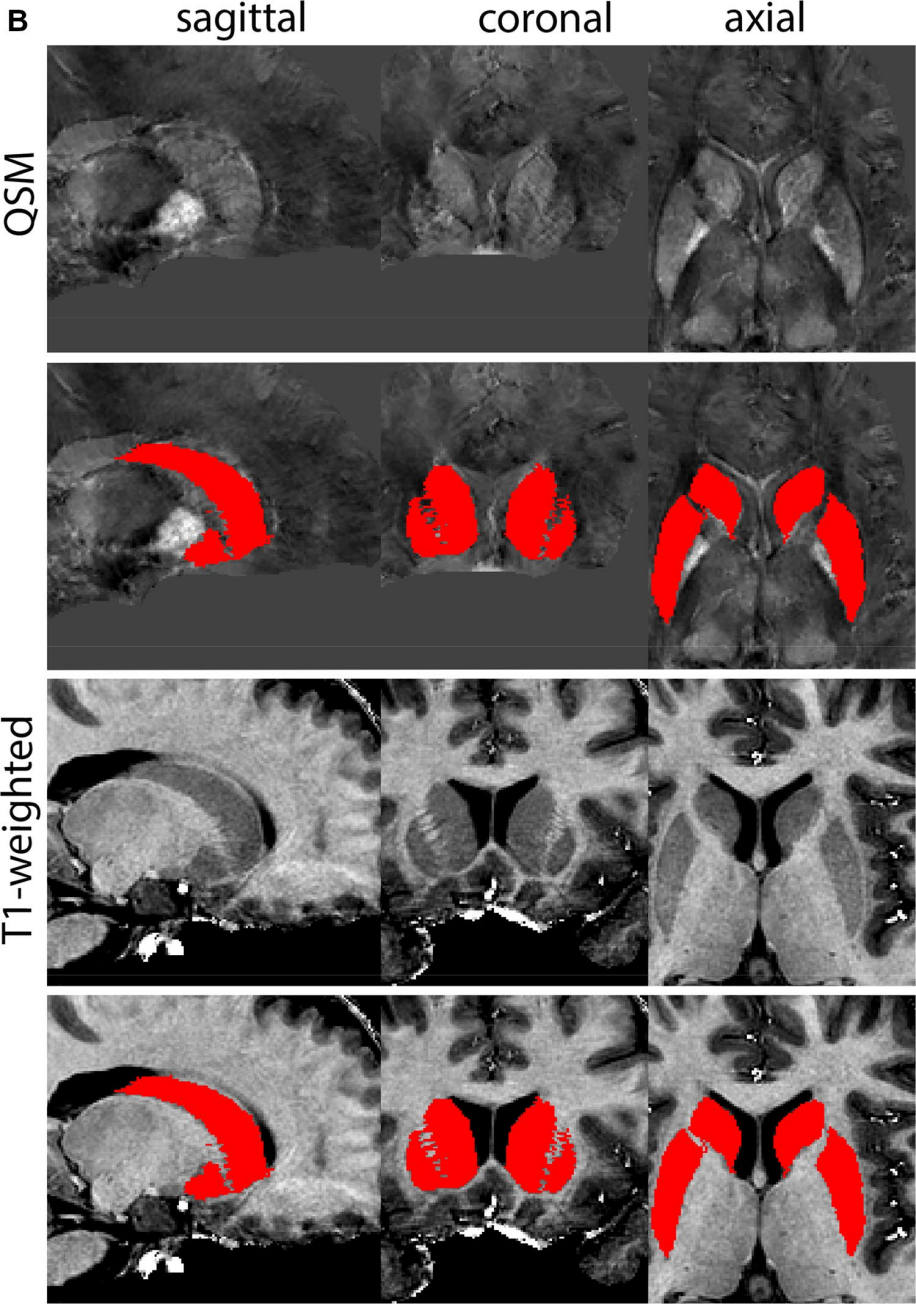

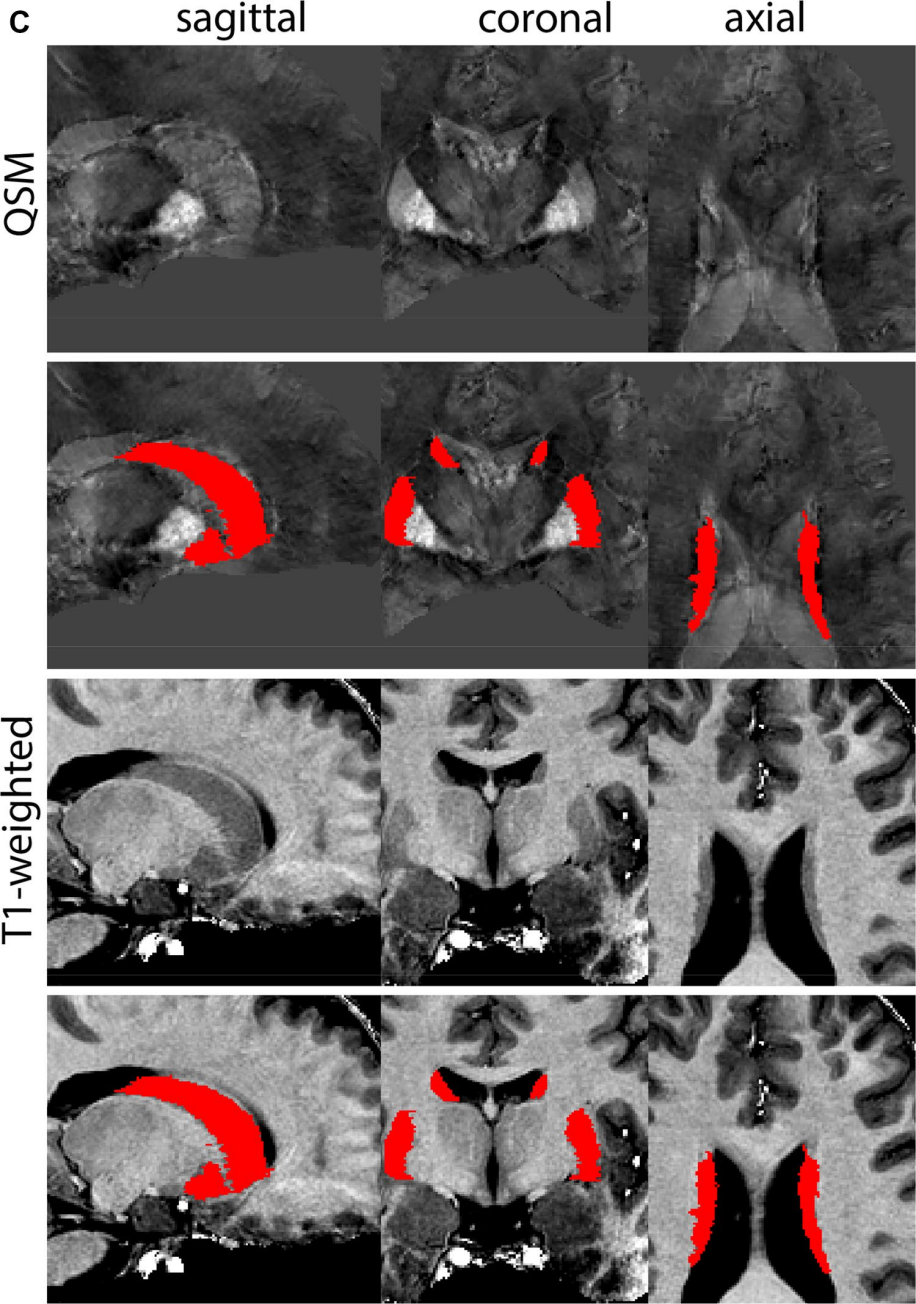

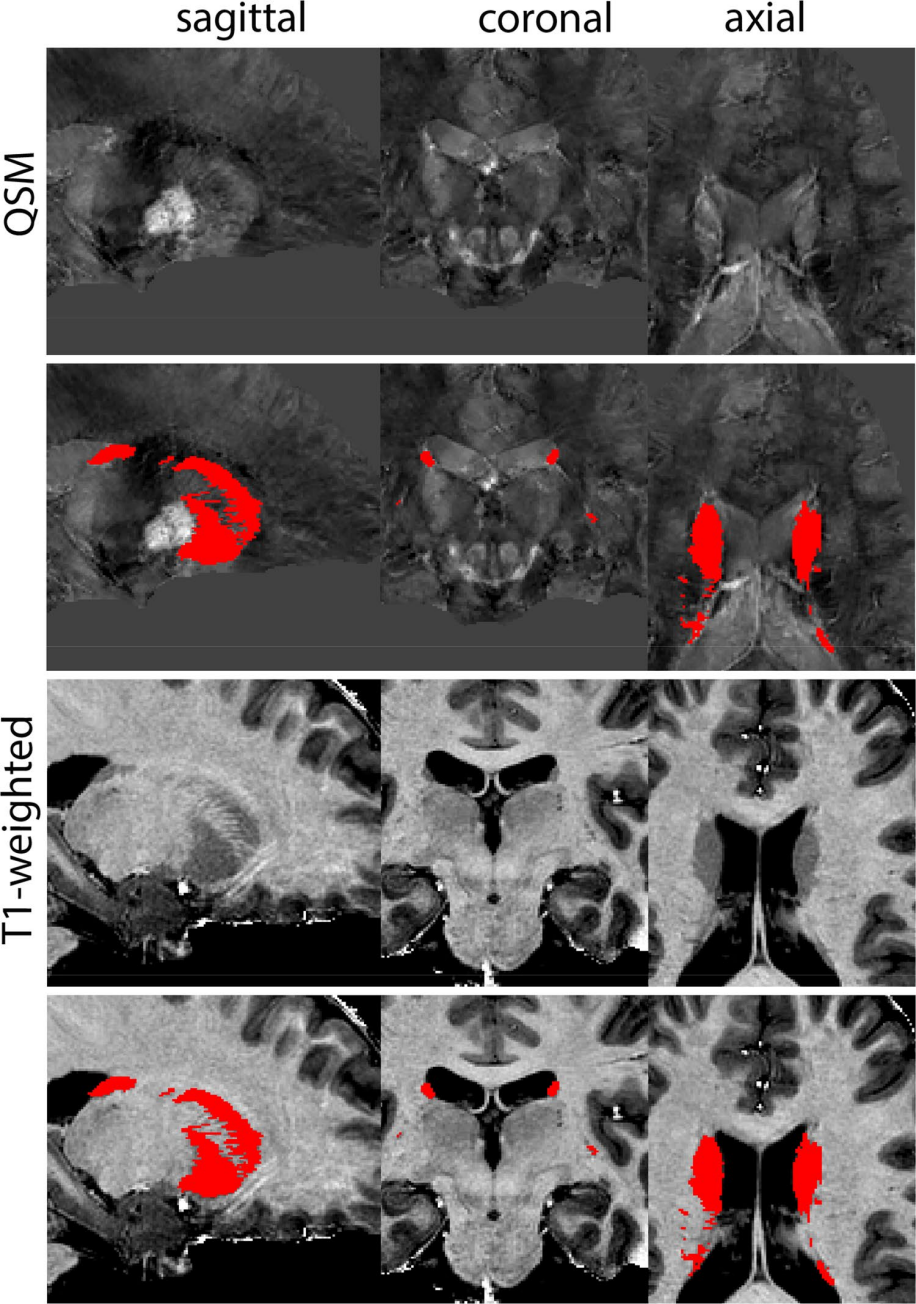
**a** The striatum (Str) at rostral levels shows the caudate nucleus. Note the clear visibility on T1-weighted contrasts. **b** The striatum (Str) at somewhat more caudal levels shows the caudate nucleus and the putamen. Note the clear striatal islands in the ic. **c** The striatum (Str) at even more caudal levels shows STR shifting in lateral direction at the level of the (hypo-) thalamus. **d** The striatum (Str) becomes patchy and disappears in the coronal plane

The border to bed nucleus of the stria terminalis cannot easily be identified based on the MR contrast. The inferomedial border of the Str is, therefore, placed at the tip of the internal capsule, providing an anatomical landmark for the delineation. Continuing in more posterior direction, the putamen moves further in lateral direction. At the most caudal extent of the Str, the putamen will form islands, and the caudate nucleus continues to decrease in size until they disappear. Parcellations are illustrated at various anatomical levels in Fig. 29.

### Thalamus

**Fig. 30:**
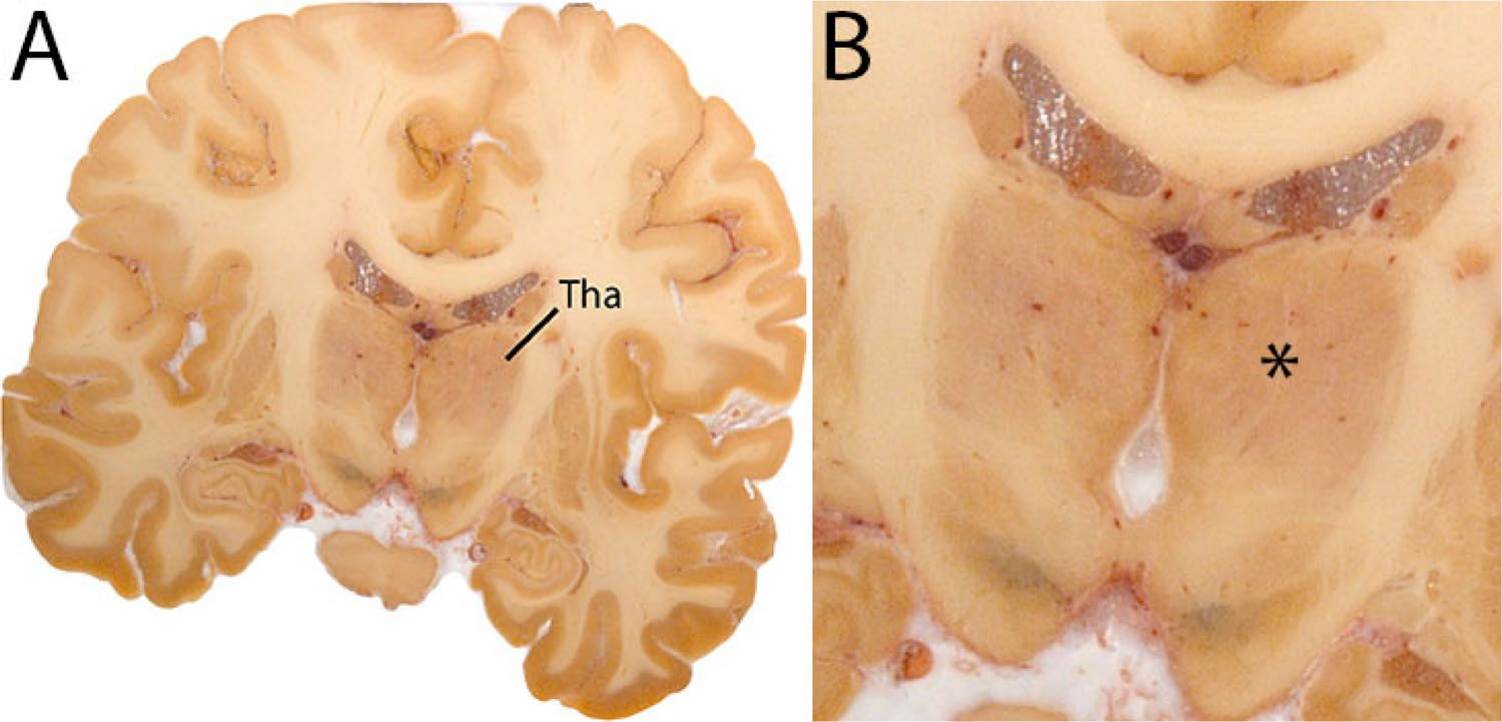
**A** The thalamus (Tha) shown here at the level of the SN and STN consists of many individual nuclei, which are bordered medially by the 3V and laterally by the white matter of the external medullary lamina. The thalamus is parcellated as a single structure. **B** Magnification, asterisk indicates Tha. Note the internal lamina visible in this image

The Tha forms a main relay station for the integration of sensory and motor signals consists of more than 20 individual nuclei. The Tha is a largely ovoid structure, which is traversed by thalamic lamina that provide additional anatomical contrast. In our protocol, the metathalamus [lateral and medial geniculate bodies (LGN and MGN)] is included in a single thalamic parcellation (Fig. 30).

#### Parcellation

Identify the location of the thalamic pulvinar nucleus in the T1-weighted images. In coronal slides it can be recognized by its characteristic oval shape located between the crus fornix, dorsally and the superior cistern ventromedially. The pineal gland is located mid-sagittal superior to the colliculi. Particularly moving in the rostral direction, the pulvinar becomes easier to distinguish. Moving in this direction, the fornix will be less helpful to define the dorsal border of the Tha. The dorsal border is clearly defined in the sagittal view, which is, therefore, used for delineations.

**Fig. 31:**
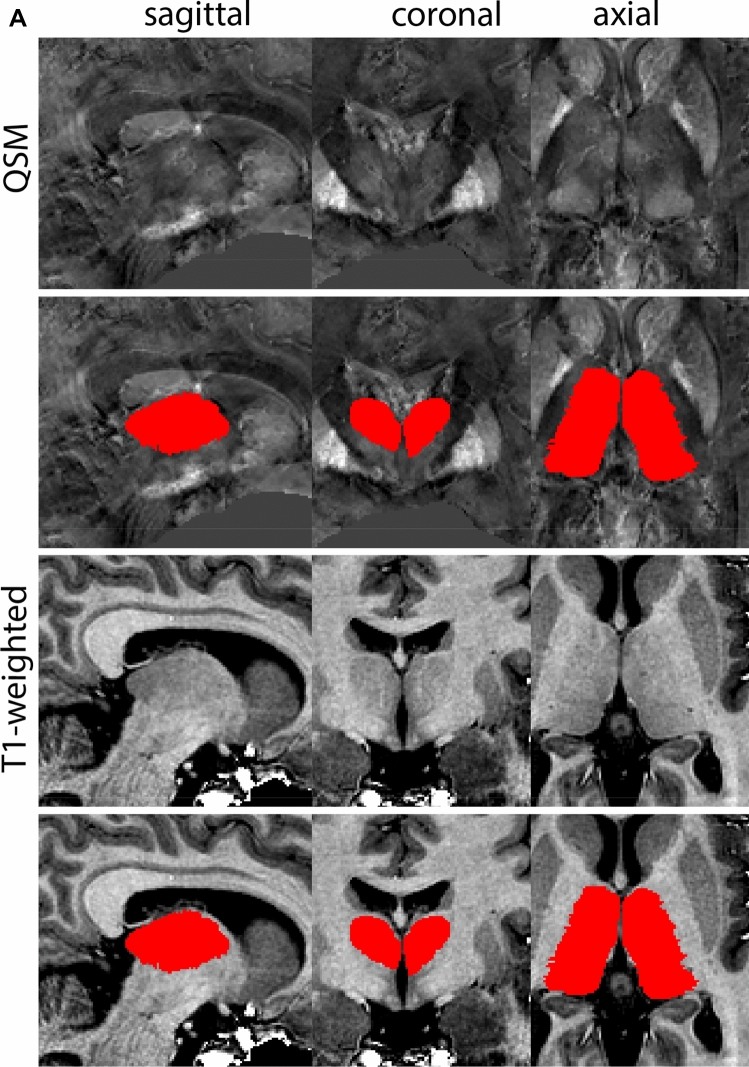

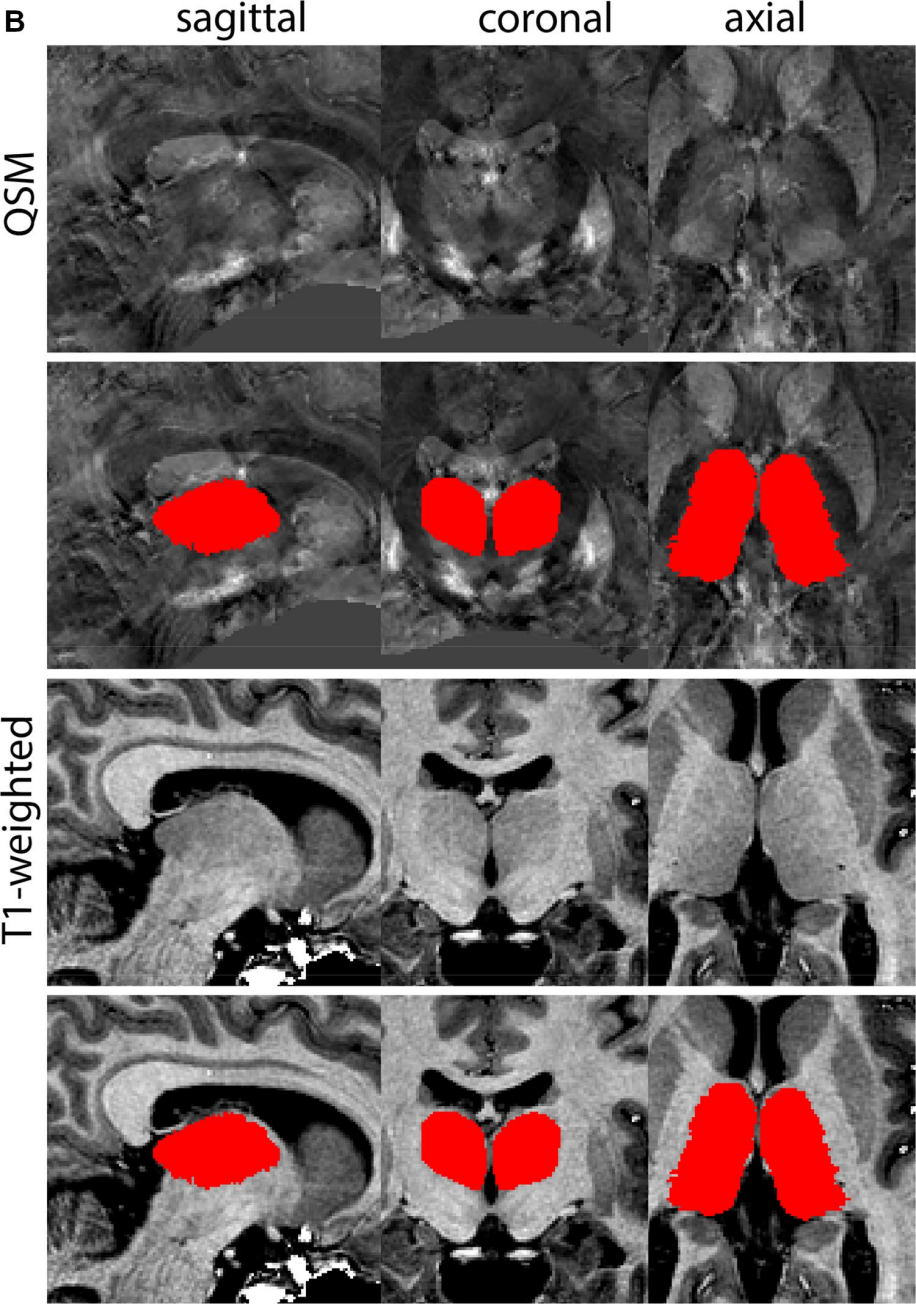

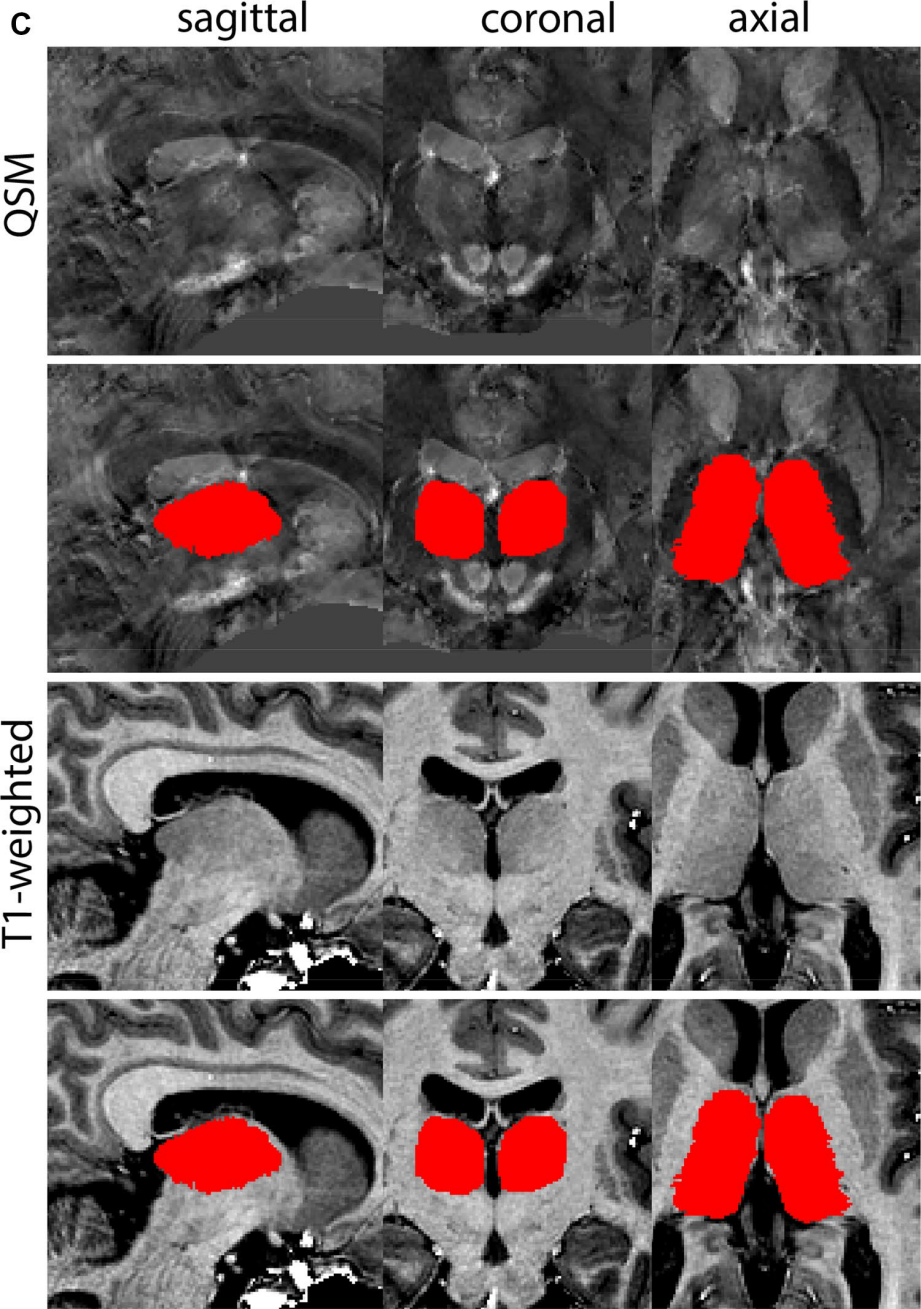

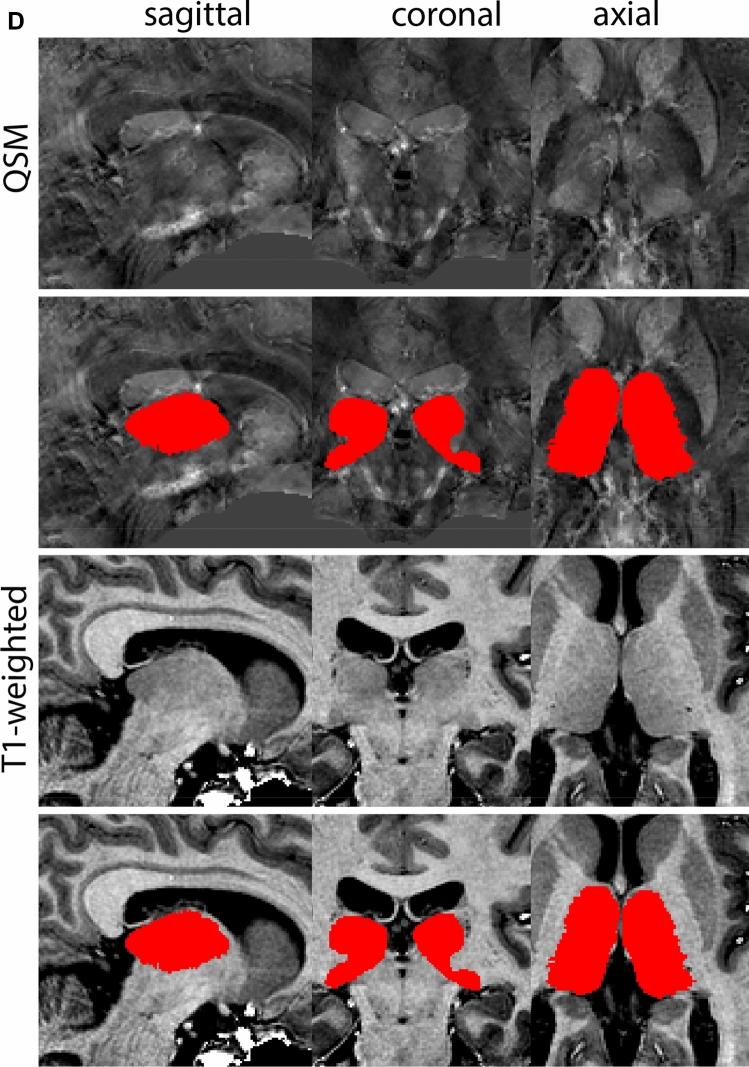

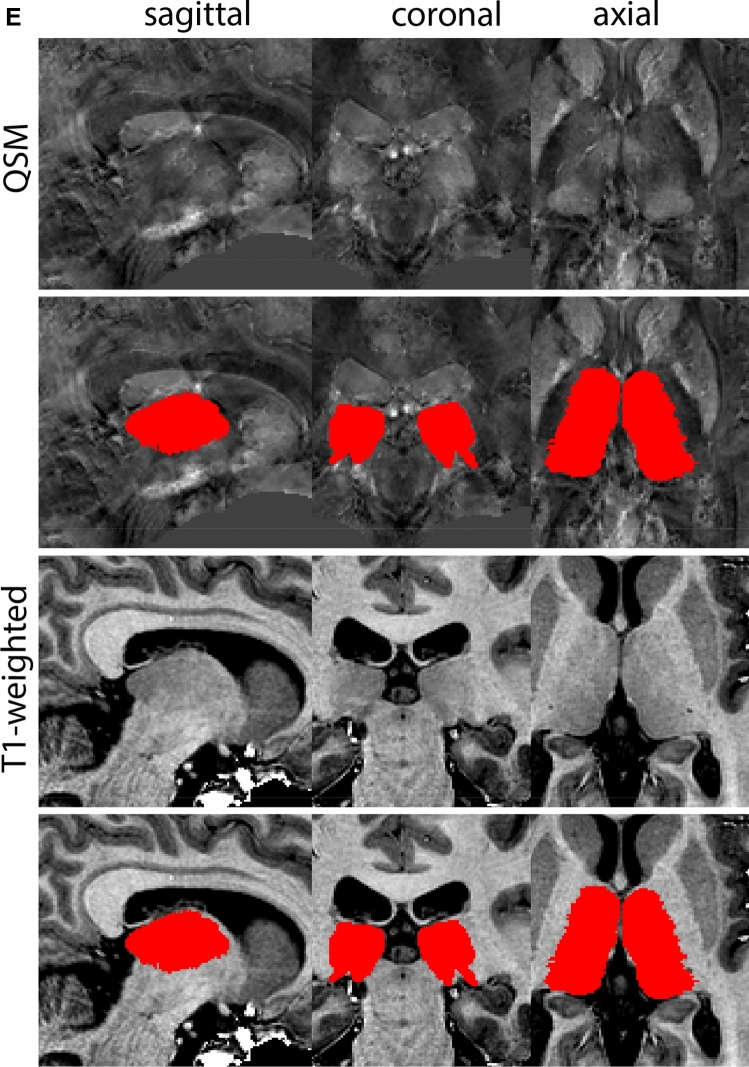

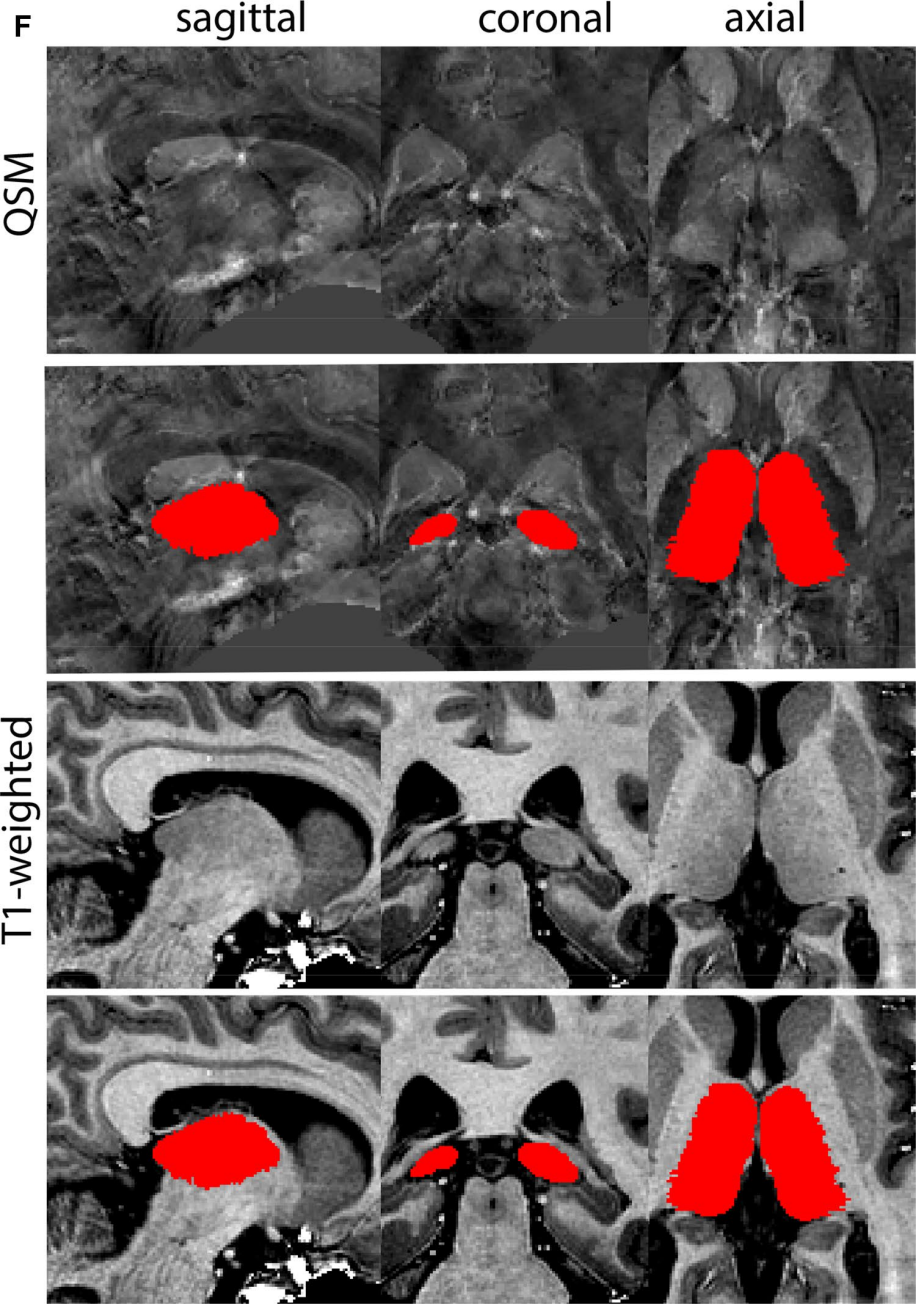
**a** The thalamus (Tha) is a large rounded ovoid structure in the diencephalon Note the location dorsal to the hypothalamic sulcus. **b** The thalamus (Tha) is a large rounded ovoid structure in the diencephalon. Note the clear visibility of the SN and S at these levels in the QSM contrast **c** The thalamus (Tha) is a large rounded ovoid structure in the diencephalon. Note the clear visibility of the SN, STN, and RN at these levels in the QSM contrast. **d** The thalamus (Tha) is a large rounded ovoid structure in the diencephalon. Note the appearance of the geniculate bodies. **e** The thalamus (Tha) is a large rounded ovoid structure in the diencephalon. Note the medial and lateral geniculate bodies, which protrude from the main structure in the coronal plane. **f** The thalamus (Tha) is a large rounded ovoid structure in the diencephalon. At caudal levels the characteristic shape of the pulvinar nucleus is clearly visible

The ventricular border continues to form an important landmark in the delineation of the Tha. The Tha will change shape in anterior direction. As the Tha connects to the midbrain, delineation of the medio-ventral border will become more challenging. The brachium of the superior colliculus (the white matter arm connecting the superior colliculus to the LGN) will briefly disrupt this border. Avoid inclusion of the brachium. Note that the portion of the superior brachium that passes between the pulvinar and the MGN is included in this delineation. As the LGN and the MGN appear, the shape becomes more irregular. Include the LGN and MGN and exclude the parcellation of surrounding white matter tracts. Make sure the habenula is not included in the parcellations. Moving anterior, the Tha is bordered by the lateral dorsally and the 3V medially. The LGN will become more separated by the cerebral peduncle in coronal views (approximately at the level of the central red nucleus). After disappearance of the geniculate bodies, the Tha will show an ovoid shape in the coronal view. At these levels, the external medullary layer will form the lateral border to the Tha. Moving further rostral, the Tha will start decreasing in size. The border between the Tha and hypothalamus is difficult to discern. The parcellation is driven by shape consistency, and the delineations should not extend below the hypothalamic sulcus. The sagittal plane can be used for guidance to determine the rostral border of the Tha. Parcellations are illustrated at various anatomical levels in Fig. 31.

### Ventricular system

The ventricular system is parcellated including the lateral ventricles (LV), as well as 3V and 4V. Additionally, their connecting aqueducts are included.

**Fig. 32:**
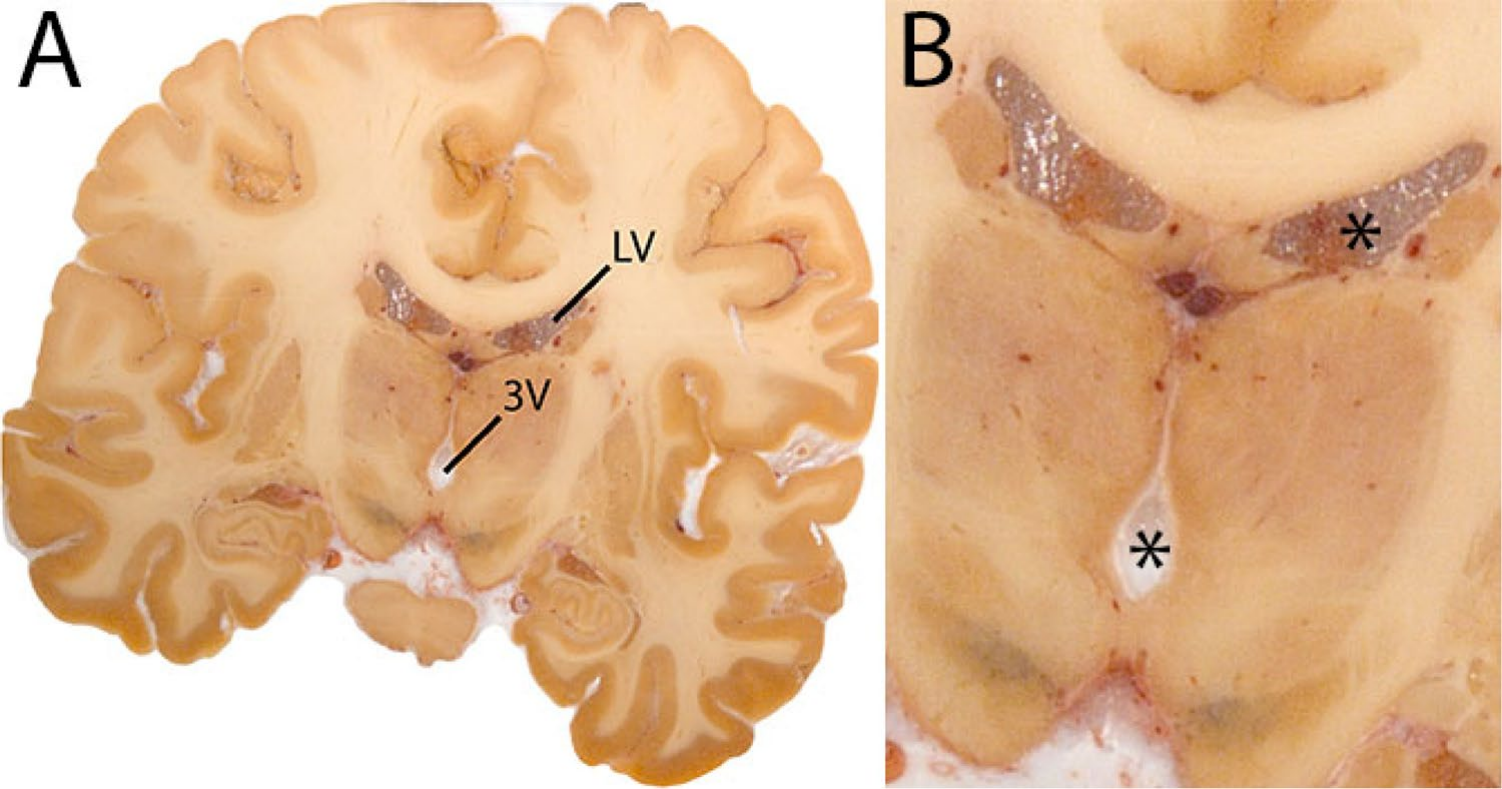
**A** The ventricular system is divided in the lateral ventricles (LV), 3V and 4V. **B** Magnification, asterisks indicate the LV and 3V

In the LV we distinguish the anterior (frontal) horns, body, atrium, posterior (occipital) horns, and inferior (temporal) horns. Two separate delineations are made for the lateral ventricles, one for each hemisphere. Delineation of the LV is followed by delineation of the 3V and the 4V. 3V and 4V are midline structures, and not delineated separate for each hemisphere (Fig. 32).

#### Parcellation

T1-weighted contrasts are used for delineations of the ventricular system, which will appear hypointense. Parcellations are first performed for the LV. Start parcellations in the frontal horns using the coronal plane. Start parcellations first moving in posterior direction. Use the axial and sagittal plane to check for consistency. Note that the ventricles may appear asymmetric. This can in part be explained by slight differences in angulation as a result of the participants’ orientation in the scanner.

### Anterior (frontal horns)

Begin segmenting the start of the frontal horn defined by a cluster of at least three voxels, which are significantly distinct in signal intensity (hypointense) compared to the surrounding white matter. The delineations can be guided almost completely by the contrast, which allows clear visualization of the ventricular borders with the corpus callosum and the caudate nucleus. Moving caudally ventricular borders are clear, except for the level of the septum pellucidum where partial voluming effects can substantially affect the visibility of the septum. If needed the location of the septum pellucidum is deduced using anatomical landmarks. Midsagittal, the left and right LV are separated through an arbitrary vertical line. Moving posterior, the frontal horns extend until the formation of the interventricular foramen. The connection to the 3V is used to demarcate the end of the frontal horns, and the start of the bodies of the LV.

### The body and atrium of the LV

The choroid plexus is located at the floor of the body of the lateral ventricles. Voxels of the choroid plexus are hyperintense compared to CSF. The choroid plexus is included in the parcellation of the ventricles. The body of the LV broadens posteriorly where it becomes continuous with the atrium. More caudally, the ventricles border the crus of the fx. The fx should not be included in the parcellations. The ventricular atrium is clearly visible at the level where the body, occipital horn, and temporal horn converge. The atrium can be recognized as a triangular shaped cavity (the trigone of the LV). The atrium connects to the posterior (occipital) horn of the LV. The occipital horn can present as two separate parts that curve around the occipital gyri. The posterior horn curves down around the thalamic pulvinar nucleus. In the coronal view the structure may sometimes appear discontinuous. Continuity is checked in the sagittal and axial planes and should be present.

The cursor is then moved to the positions where the temporal horn merges with the body of the LV. In the coronal view, locate the position where the horns merge into the body at the atrium and then segment in the anterior direction from this point. At more rostral levels the amygdala will appear. The temporal horns will soon disappear at these levels. Since the temporal horns can be difficult to discern in the coronal plane, the sagittal view is used to correct and complete the delineation of this part of the LV. The temporal horns will appear contrasted against surrounding white matter as a horizontal streak of hypointense voxels. The temporal horns will extend and curve around the hippocampus more centrally. The choroid plexus that forms the floor of the LV extends into the 3 V through the interventricular foramen. The presence of the choroid plexus, combined with the interventricular foramen, can interfere with the visualization of the border with the Tha/stria terminalis.

### The third ventricle (3V)

The 3V is located at the midline of the brain. The choroid plexus of the LV will extend into the 3V through the interventricular foramen. Include choroid plexus present in the 3V in the delineation as part of the ventricle. The first coronal slice considered part of the 3V is where the optic recess connects to form the hypothalamus. Note that the anterior commissure can cross the 3V thalamic adhesion and divide the 3V into two separate parts in the coronal view. Make sure the pineal body is excluded from the segmentation. Continue delineations in the coronal plane, following the slices in a posterior direction. The last slice of the 3V is where the 3V becomes detached from the quadrigeminal cistern.

### The fourth ventricle (4V) and cerebral aqueduct

**Fig. 33:**
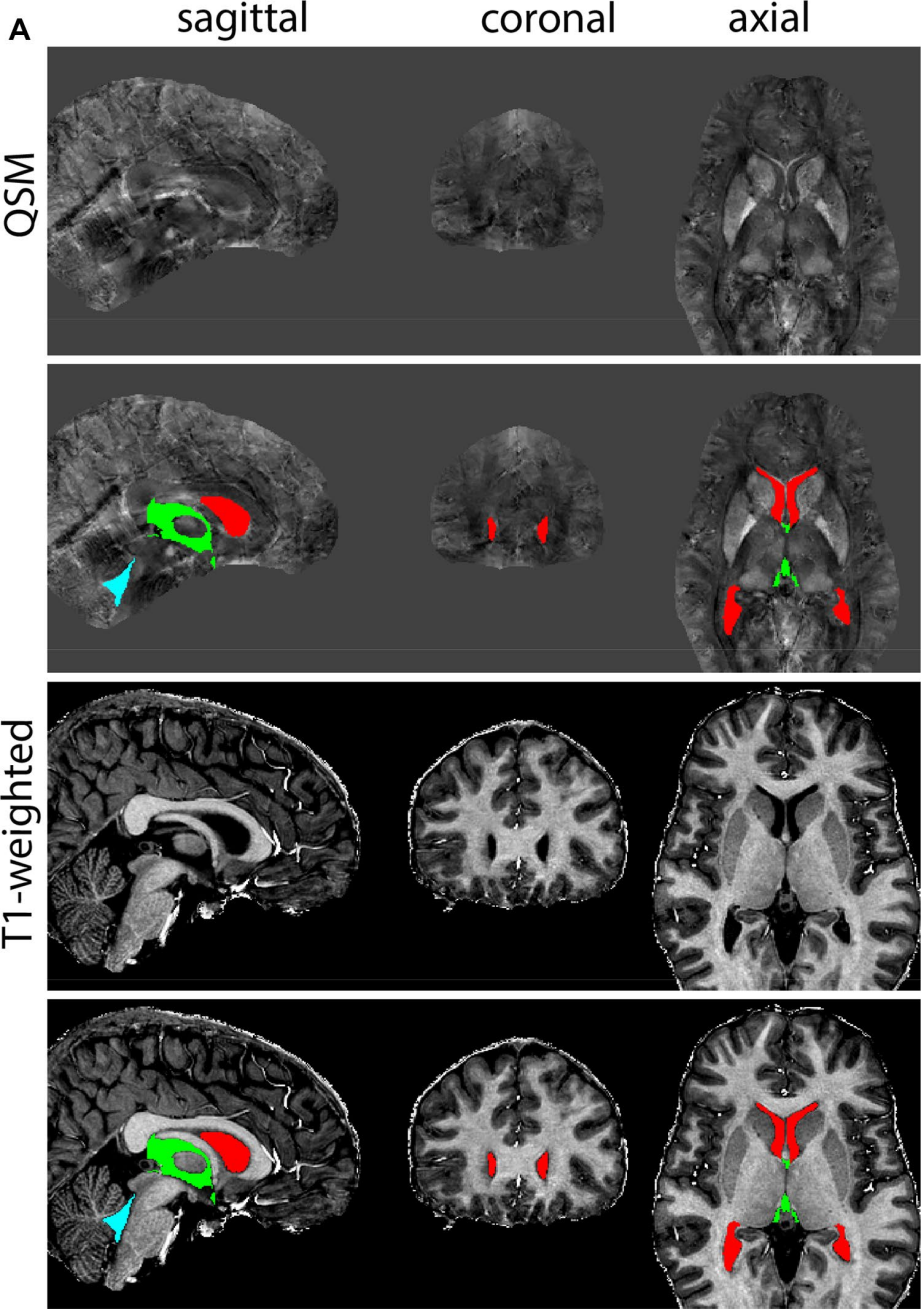

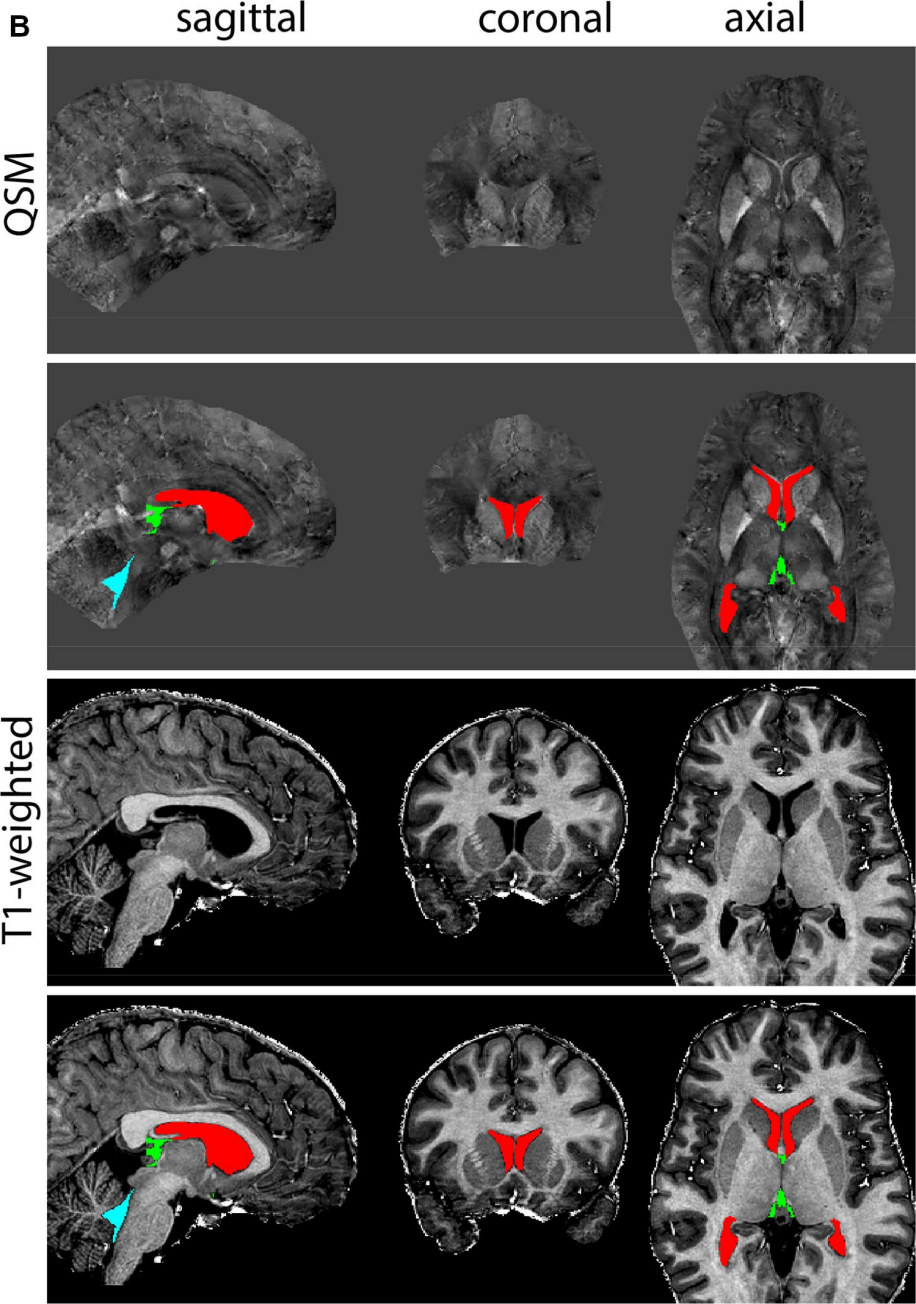

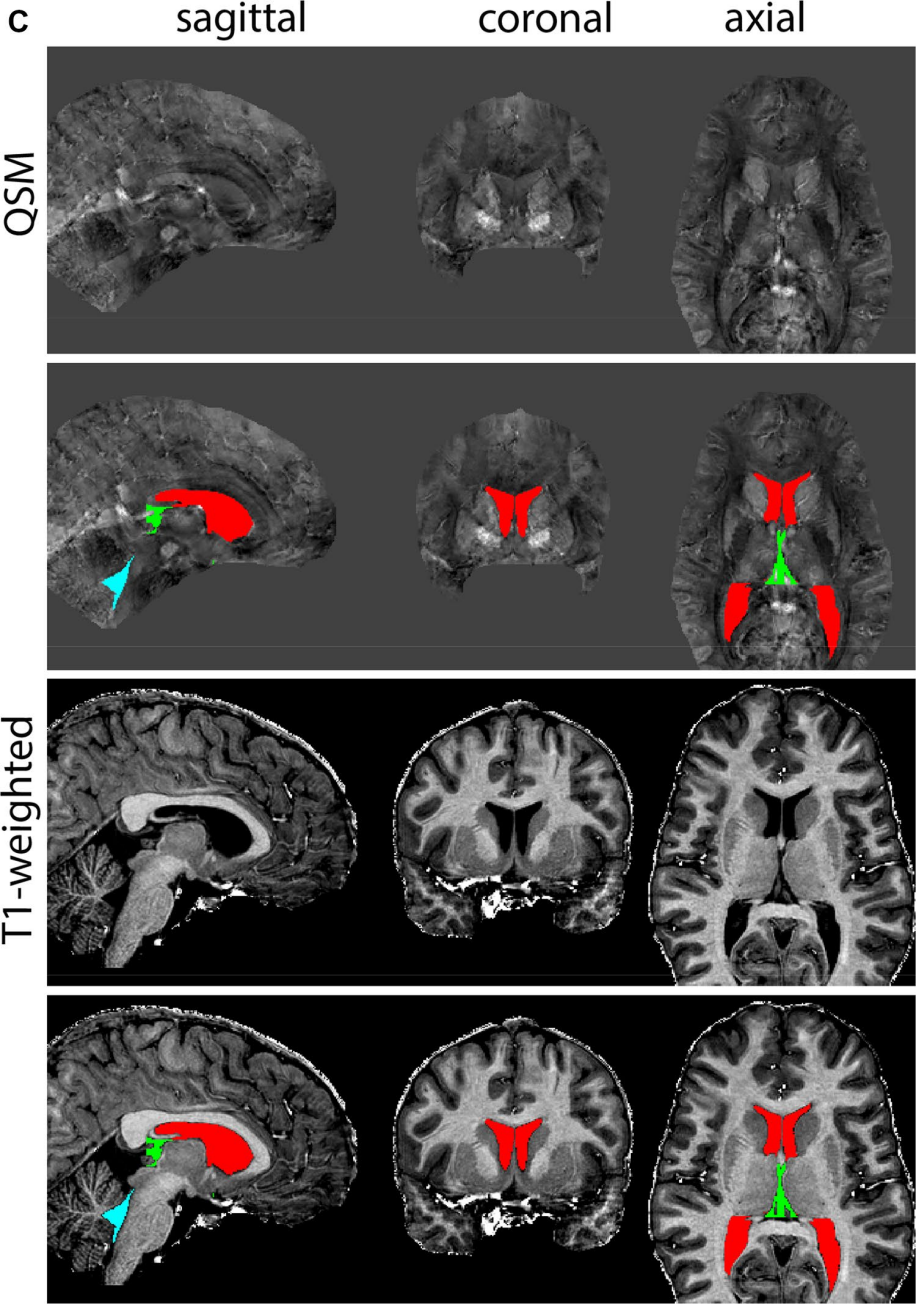

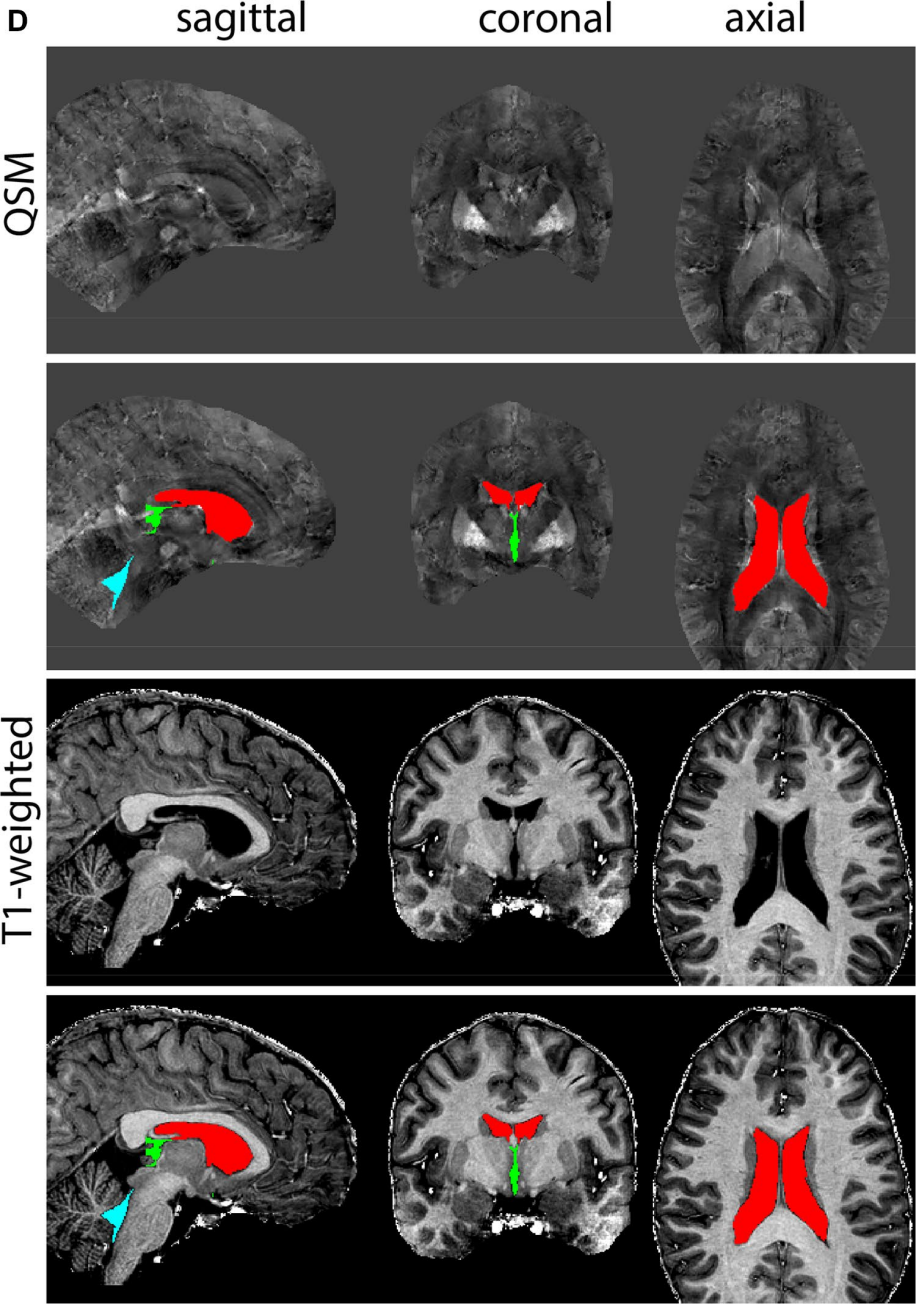

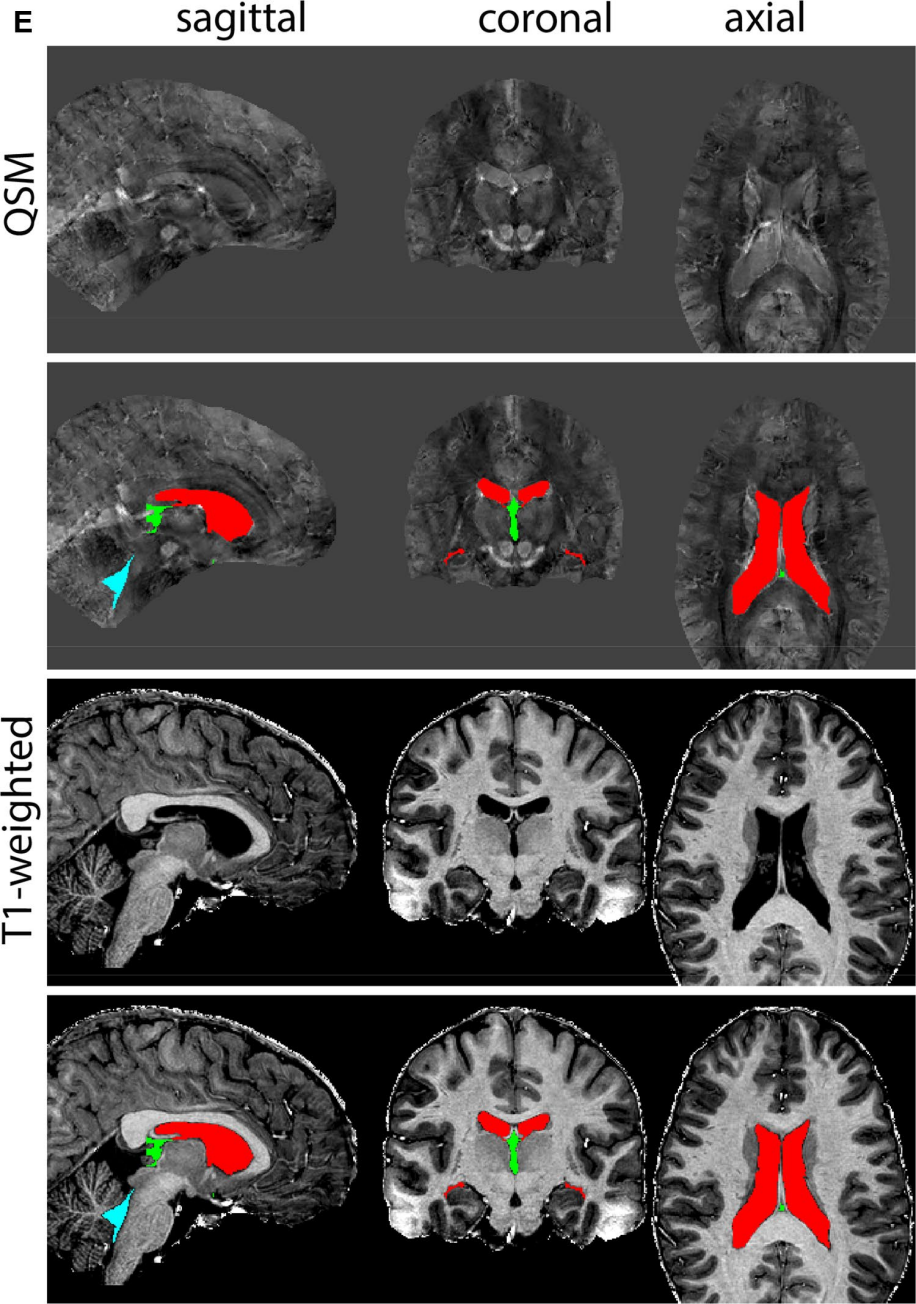

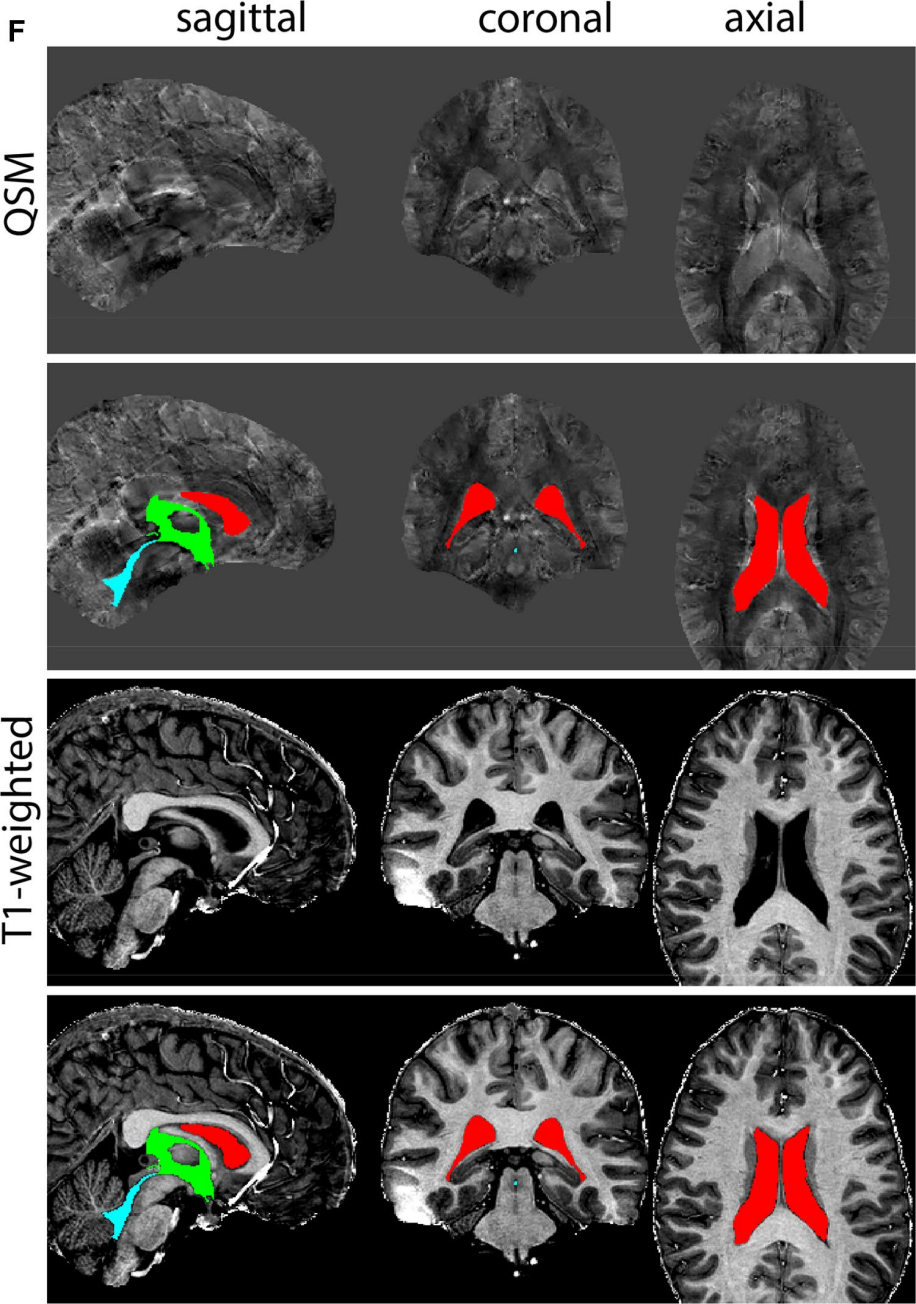

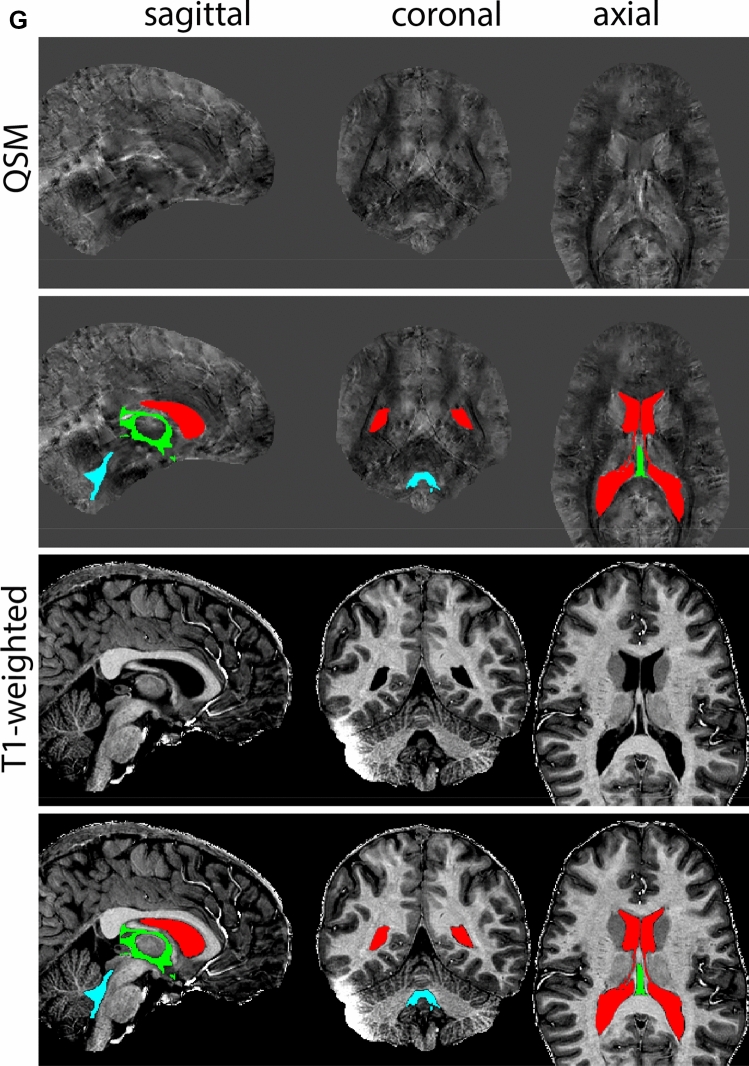
**a** The ventricular system parcellation includes the connecting aqueducts between the ventricles. Note the clear visibility on T1-weighted contrasts. LV is shown in red, 3V in green and 4V in turquois. **b** The ventricular system parcellation includes the connecting aqueducts between the ventricles. Note the clear visibility on T1-weighted contrasts. LV is shown in red, 3V in green and 4V in turquois. **c** The ventricular system parcellation includes the connecting aqueducts between the ventricles. Note the coronal level, anterior to the attachment of the optic chiasm to the mediobasal hypothalamus to form the 3V. LV is shown in red, 3V in green and 4V in turquois. **d** The ventricular system parcellation includes the connecting aqueducts between the ventricles. Note the coronal level, where the 3V and LV connect. LV is shown in red, 3V in green and 4V in turquois. **e** The ventricular system parcellation includes the connecting aqueducts between the ventricles. Note the coronal level, where the temporal horn of the LV is barely visible. LV is shown in red, 3V in green and 4V in turquois. **f** The ventricular system parcellation includes the connecting aqueducts between the ventricles. Note the shape of the LV in the coronal level. LV is shown in red, 3V in green and 4V in turquois. **g** The ventricular system parcellation includes the connecting aqueducts between the ventricles. Note the shape of the LV in the coronal level towards their most caudal extent. LV is shown in red, 3V in green and 4V in turquois

The 4V is best segmented in the sagittal view in which it is easiest to discern. The parcellations are reviewed in the coronal plane. Start parcellations at a central level of the 4V, and move in a lateral direction. At its first appearance, the 4V can be discerned by the CSF contrast appearing within the cerebellum. In levels that are close to the midsagittal line, the cerebral aqueduct connecting the 3V and 4V will be visible. In more lateral slices, the cerebral aqueduct will not be present. The 4V is continuous with the cisterna cerebellomedullaris. The border between the cisterna and the 4V is defined by drawing a line at the border of the pons and medulla oblongata. Voxels on this line are excluded, and voxels above this line are included in the parcellations of 4V. The same procedure is followed with other bordering cisterns. Parcellations of the ventricular system are illustrated at various anatomical levels in Fig. 33.

### Ventral tegmental area

**Fig. 34:**
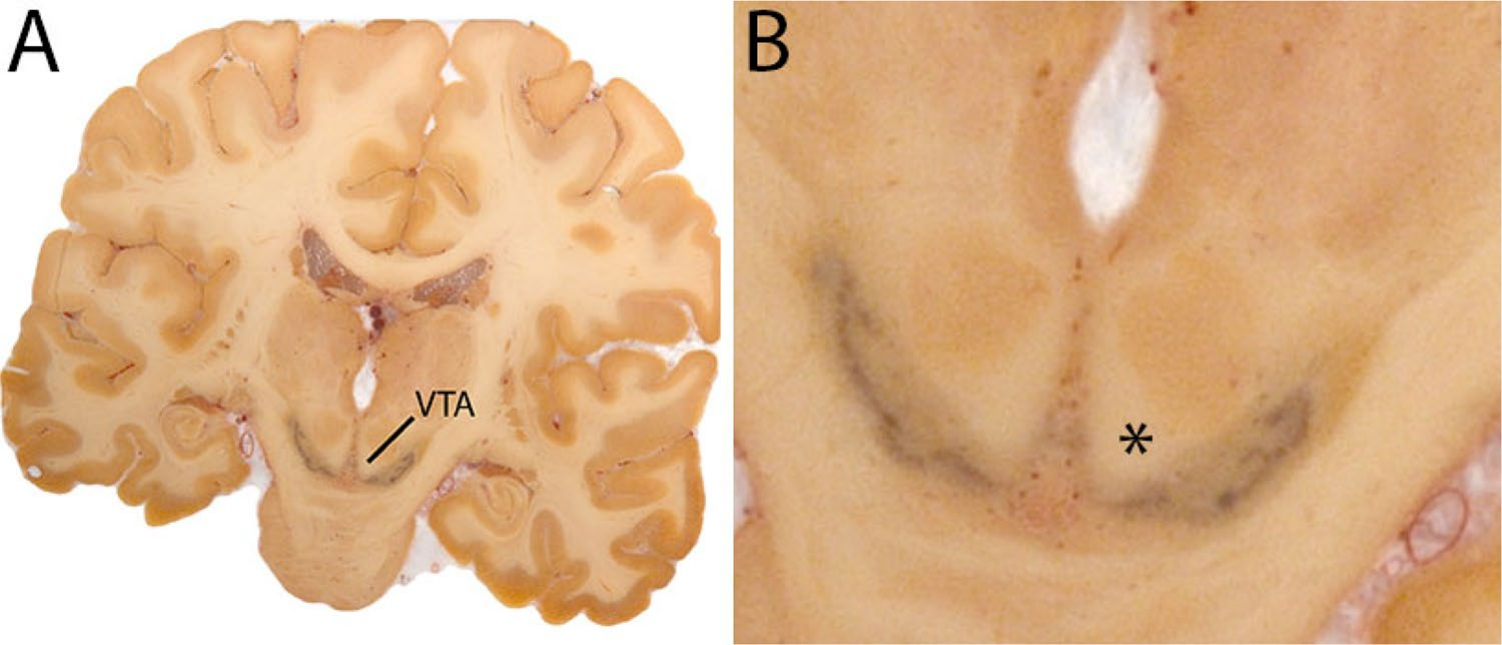
**A** The ventral tegmental area (VTA) has poorly defined anatomical borders, and is mainly delineated based on anatomical landmarks including the RN and SN. **B** Magnification, VTA is indicated by the asterisk. Note the clear appearance of the RN and SN which are used as landmarks

The ventral tegmental area (VTA) consists of a number of individually distinct, but anatomically poorly defined structures. Due to the poorly defined anatomical border delineations are strongly dependent on anatomical landmarks. The VTA is shaped around the RN. The parabrachial pigmented and paranigral nucleus located at the lateral extent of the VTA form the main dopaminergic substructures of the VTA (Fig. 34).

#### Parcellation

**Fig. 35:**
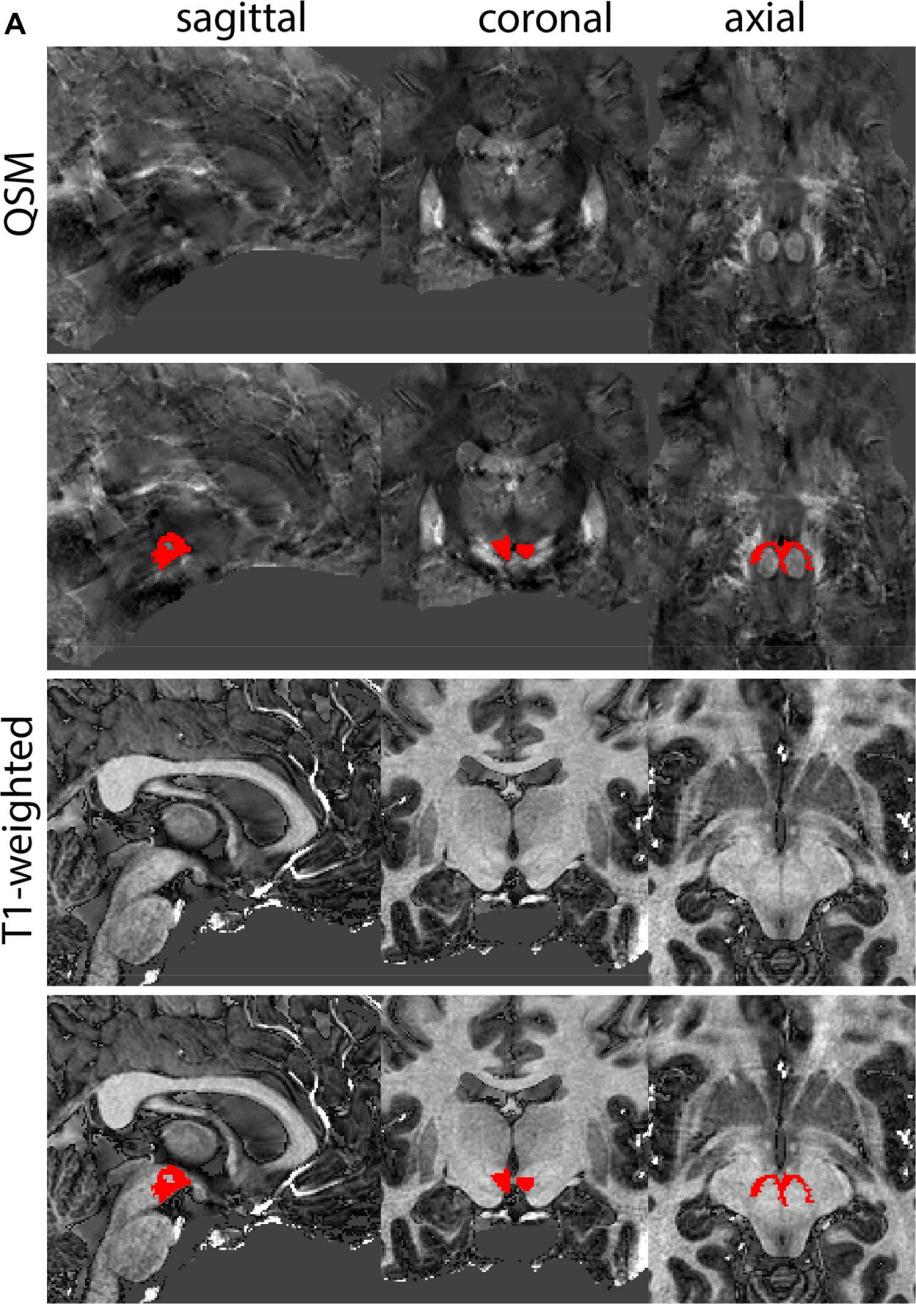

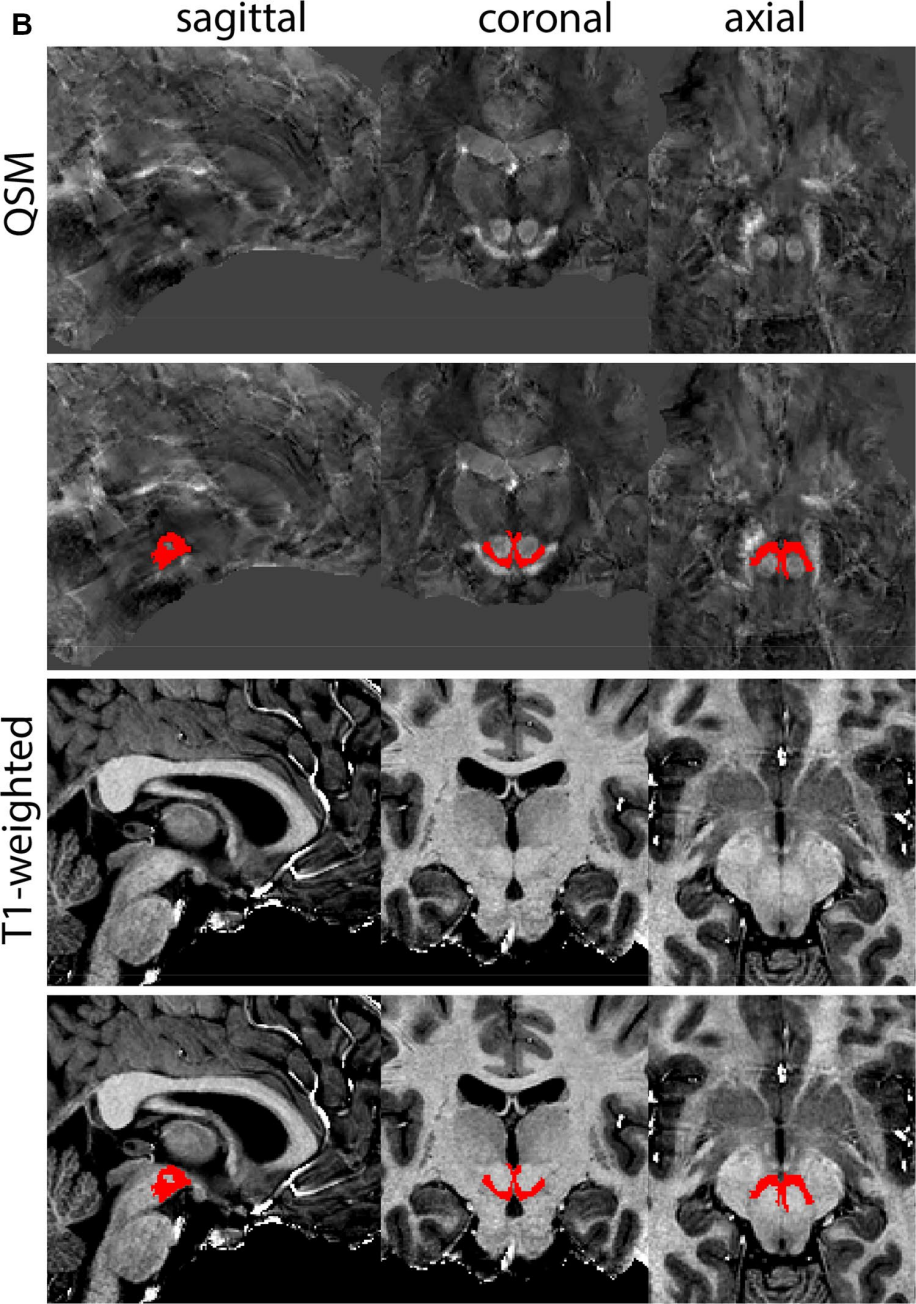

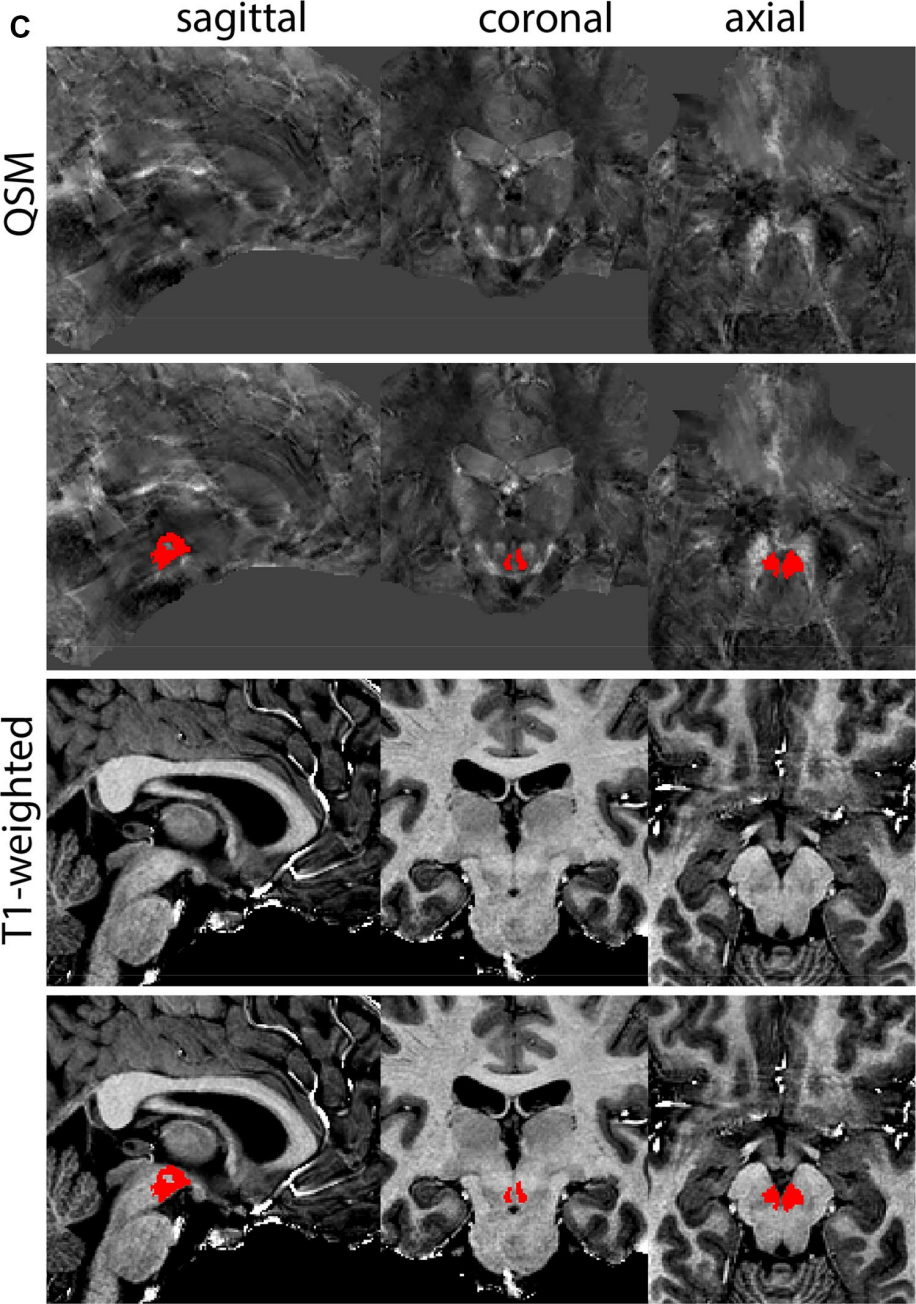
**a** The ventral tegmental area (VTA) curves around the RN, which is clearly visible in the axial plane in QSM contrasts at this level. **b** The ventral tegmental area (VTA) curves around the RN, which is clearly visible in the coronal and axial plane in QSM contrasts at this level. **c** The ventral tegmental area (VTA) at a more caudal coronal level

The VTA can be parcellated based on T1-weighted and T2*-weighted images. QSM images can be used for verification of the location of the SN, RN and STN. We would like to note that for ours we made use of a PCA-contrast as described by Trutti et al. (2021). First locate the mamillary bodies (MB) in each available view on the T1-weighted contrast. In sagittal view, move from the MB in posterior direction along the tract that connects the MB to the midbrain, until the peak curvature. Parcellations are started at this point and are performed in the axial plane. The VTA is located between RN (dorsally), SN (ventrolaterally), the cerebral aqueduct (medially) and the fourth ventricle (ventromedially). Parcellate using the dorsal border of the SN to demarcate the lateral VTA border. Do not include bordering fiber tracts in the parcellations. Continue parcellations further first in the dorsal followed by the ventral direction. The lateral VTA does not extend dorsally to the same level as the medial VTA. The main part of the structure is parcellated as the center between the RN, SN, and ventricular system. Make sure using the sagittal view that the parcellations do not extend into the CSF. At more ventral levels the lateral VTA decreases in size. Parcellate the VTA in ventral direction until the disappearance of the RN, and if feasible further to the point where the SN borders the CSF in coronal view. At more caudal levels the lateral VTA disappears. Parcellations are illustrated at various anatomical levels in Fig. 35.

## Results

Manual parcellations were performed by two individual raters for each structure on ten individual participants. Structures crossing the midline were not separated into observations for left and right hemispheres. In Table 1 Dice similarity coefficients and dilated Dice coefficients are presented.

## Discussion

The human subcortex comprises a wealth of individual brain structures that are only sparsely mapped and included in commonly used brain atlases for MRI (Alkemade et al. 2013). Here, we present delineation protocols of 21 of these structures (Table 1), together with our results supporting the replicability of the masks when using these protocols. The illustrations presented in the protocols are not exhaustive. We have incorporated slice by slice information through the sharing of three individual datasets, which provides a more informative alternative as it shows the parcellations in the coronal, axial, and sagittal planes (see data availability). Decisions to include or exclude voxels vary between slices when following a slice by slice procedure as our protocols describe. This can result in a jagged appearance of individual structure. Smoothing of these edges would require additional arbitrary decisions, and are unlikely to substantially influence the parcellation results other than their jagged appearance, nor the use of the parcellations in further analyses. We therefore decided not to smooth the parcellation results.

With these descriptions, we share a methodology that can be reused by other research groups for manual parcellation purposes, including validation of automated delineation procedures in comparable as well as different MRI contrasts. We confirmed that these protocols were intelligible for both trained and less experienced anatomists, which performed the parcellations through calculation of Dice similarity, and dilated Dice similarity coefficients. The typical reported range of a good intra-rater reliability is higher than 0.75 as reflected by the Dice similarity coefficient (Dice and Dice 1945). We observed that structures with a limited diameter in any direction and/or delineated based on landmarks, such as the VTA, SCG and the PPN, tended to have somewhat lower Dice similarity coefficients as compared to those with borders defined based on image contrast, as well as larger structures. For such structures the Dice similarity coefficient may result in an underestimation of the interrater agreement. The dilated Dice score is a potentially more representative measure for the agreement between raters regardless of size, as the smaller structures can reach high levels of overlap (Bazin et al. 2016, 2020; Trutti et al. 2021). The dilated Dice score (0.82–1.00, Table 1), which allows a single voxel of uncertainty indicates that the observed variation results mainly from differences in border detection, and not structural differences in the parcellation approaches of the individual raters. In some cases, borders between two structures may be difficult to detect, in part due to partial voluming effects. This may result in the attribution of some voxels to more than one individual structure. The use of conjunct masks reduces this problem.

We would like to emphasize that we developed and validated these protocols on data of the AHEAD, which is a 7T anatomical dataset, acquired with submillimeter resolution (Alkemade et al. 2020a). The question, therefore, rises to what extent the presented protocols can be reused for clinical datasets. We have previously published on the parcellation of the GPi/e, SN, RN and STN in ten participants of the AHEAD, which were also scanned in a 3T scanner using a clinical scan and an optimized scan. The results confirmed reliable parcellation in optimized scans with a 1-mm isotropic voxel resolution. However, manual parcellations did not yield an anatomically plausible shape of the STN using the clinical scan. This was attributed to the use anisotropic voxels (Mulder et al. 2019; Isaacs et al. 2020). With the approval of 7T systems for clinical practice, the use of UHF submillimeter imaging for the identification of DBS targets in the clinic now comes within reach.

The parcellation protocols presented, in some cases, led to the inclusion of (parts of) adjacent structures, or the omission of part of the structure of interest. These arbitrary choices were included in the parcellation protocols for practical reasons and in most cases were guided by a lack of anatomical contrast, which hampered a more precise border determination. Ongoing developments in MRI hardware and scan acquisition protocols are expected to improve anatomical contrast even further, which may warrant updating of the presented descriptions accordingly.

Automated anatomical labeling procedures provide the promise of reliable identification and parcellation of structures of interest, although anatomical validation is required to confirm the correct identification of the anatomy (Poldrack 2007). The presented parcellation procedures have formed the basis for the training and validation of the MASSP algorithm (Bazin et al. 2020) and thus can be reused by researchers aiming to operationalize the use of MASSP on different MRI datasets, allowing them to apply the same level of rigor as we did in the validation of the algorithm for use on the 7T MP2RAGEME contrasts as described by Caan et al. (2019). With adaptions these protocols are expected to be of value as well for their use on different MRI contrasts due to the general knowledge they provide on the shape and size as well as the spatial orientation of and between the structures in the descriptions. The development of automated anatomical labeling procedures represents a growing field of research. These efforts are invaluable for the analyses of large neuroimaging datasets such as the human connectome project (Van Essen et al. 2012; Glasser et al. 2016), which in view of the laborious nature of manual parcellation does not lend itself for manual assessments of individual neuroanatomy. Automated procedures are, therefore, indispensable and require reproducible validation strategies to ensure the quality of automated labeling procedures performed in datasets with slightly different contrast characteristics.

A major challenge is to capture the substantial anatomical variability that is observed between participants. Variability occurs not only in shape, size, and location, but also in iron content, and MRI characteristics as a result of aging and disease (Draganski et al. 2011; Fjell et al. 2013; Keuken et al. 2013, 2017; Andersen et al. 2014; Yeatman et al. 2014; Alkemade et al. 2017; Zhang et al. 2018; Herting et al. 2018; Hill et al. 2018; Turner 2019). It is possible that this variability contributes to differences in parcellation results obtained using both manual and automated parcellation procedures. Validation of both the automated and manual parcellation procedures is, therefore, warranted when applying existing methods on participant cohorts that differ in scan acquisition parameters, and/or aging and disease status. This validation can be achieved by determining Dice similarity coefficients and dilated Dice coefficients between manual and automated parcellation results.

Comparison of anatomical variation can broaden our understanding the normal and aberrant brain anatomy, including healthy aging as well as the development of atrophy as a result of neurodegenerative disease. In general, high-quality training data will contribute to a high performance of the algorithm implemented for automated parcellation. The use of standardized manual parcellation protocols will, therefore, contribute to the performance of automated parcellation procedures.

## Data Availability

Datasets referenced in the manuscript can be downloaded freely and without restrictions via https://doi.org/10.21942/uva.14216504.

## Abbreviations

3D: 3-Dimensional
3V: Third ventricle
4V: Fourth ventricle
AHEAD: Amsterdam ultra-high field adult lifespan database
Amg: Amygdala
Cl: Claustrum
DBS: Deep brain stimulation
EC: Entorhinal cortex
ES: Entorhinal sulcus
fr: Fasciculus retroflexus
fx: Fornix
GP: Globus pallidus
GPe: Globus pallidus, external segment
GPi: Globus pallidus, internal segment
ic: Internal capsule
iCo: Inferior colliculus
LH: Lateral habenula
lml: Lateral medullary lamina
LV: Lateral ventricle
MB: Mamillary bodies
mml: Medial medullary lamina
MRI: Magnetic resonance imaging
ot: Optic tract
PAC: Periamygaloid cortex
PAG: Periaqueductal grey
PVG: Periventricular grey
QSM: Quantitative susceptibility mapping
RN: Red nucleus
SCG: Subcallosal cingulate gyrus
sCo: Superior colliculus
sm: Stria medullaris
SN: Substantia nigra
SNp: Substantia nigra, compact part
SNR: Substantia nigra, reticular part
STN: Subthalamic nucleus
Str: Striatum
T: Tesla
Tha: Thalamus
VTA: Ventral tegmental area
WM: White matter
